# Supplementary material for: Functional editing of the OTC locus by targeted integration with phenotype correction and restoration of endogenous expression patterns
Source: Mol Ther. 2026 Jul 9;34(9):5426–43. doi: 10.1016/j.ymthe.2026.06.044 (PMC13555538; doi:10.1016/j.ymthe.2026.06.044)
Supplement: Supplementary file 1 [file mmc1.pdf]

YMTHE, Volume *34*

Supplemental Information

Functional editing of the OTC locus by targeted integration with phenotype correction and restoration of endogenous expression patterns

Samantha L. Ginn, Fatemeh Doroudian, Sharntie Christina, Otilia P.Y. Chan, Caitlin W. Lucas, Erhua Zhu, Sile F. Yang, Beena Devanapalli, Anne H. Klein, Suzanne Scott, Joseph Vitale, Sharon C. Cunningham, Sophia H.Y. Liao, Marti Cabanes-Creus, Leszek Lisowski, and Ian E. Alexander

Table S1: *In silico* sgRNA selection using Benchling (mouse targeting)

	Sequence (5'to 3')	PAM	Specificity score	Efficiency score
sgRNA1	ACTGCCCATAGAGAAACAAGA	CTGAAT	87.7	35.3
sgRNA2	GCCAATTCTCTGTAGGCACCG	GGGGGT	76.9	47.4
sgRNA3	GAATACAATCCTGGGGCCAAT	GAGAAT	45.5	45.7

Table S2. Statistical analysis of Figure 2C (quantification of transcripts from the *Otc* promoter)

Endogenous transcripts

Tukey's multiple comparisons test	Mean diff.	95.00% CI of diff.	Below threshold?	Summary	Adjusted P Value
2:1 vs. 1:2	-22.65	-35.56 to -9.735	Yes	***	0.0001
2:1 vs. 1:1	-17.79	-30.71 to -4.879	Yes	**	0.0026
2:1 vs. 1:0.5	-8.614	-21.53 to 4.299	No	ns	0.3712
2:1 vs. 0.5:0.5	-37.33	-50.25 to -24.42	Yes	****	<0.0001
2:1 vs. Donor	-80.75	-93.67 to -67.84	Yes	****	<0.0001
2:1 vs. Cas9	-80.65	-93.57 to -67.74	Yes	****	<0.0001
1:2 vs. 1:1	4.856	-8.057 to 17.77	No	ns	0.8910
1:2 vs. 1:0.5	14.03	1.121 to 26.95	Yes	*	0.0265
1:2 vs. 0.5:0.5	-14.68	-27.60 to -1.771	Yes	*	0.0181
1:2 vs. Donor	-58.10	-71.02 to -45.19	Yes	****	<0.0001
1:2 vs. Cas9	-58.00	-70.92 to -45.09	Yes	****	<0.0001
1:1 vs. 1:0.5	9.178	-3.735 to 22.09	No	ns	0.3003
1:1 vs. 0.5:0.5	-19.54	-32.45 to -6.627	Yes	***	0.0008
1:1 vs. Donor	-62.96	-75.87 to -50.05	Yes	****	<0.0001
1:1 vs. Cas9	-62.86	-75.77 to -49.95	Yes	****	<0.0001
1:0.5 vs. 0.5:0.5	-28.72	-41.63 to -15.80	Yes	****	<0.0001
1:0.5 vs. Donor	-72.14	-85.05 to -59.22	Yes	****	<0.0001
1:0.5 vs. Cas9	-72.04	-84.95 to -59.12	Yes	****	<0.0001
0.5:0.5 vs. Donor	-43.42	-56.33 to -30.51	Yes	****	<0.0001
0.5:0.5 vs. Cas9	-43.32	-56.23 to -30.41	Yes	****	<0.0001
Donor vs. Cas9	0.1000	-12.81 to 13.01	No	ns	>0.9999

Test details	Mean 1	Mean 2	Mean diff.	SE of diff.	n1	n2	q	DF
2:1 vs. 1:2	19.20	41.85	-22.65	4.071	5	5	7.868	28
2:1 vs. 1:1	19.20	37.00	-17.79	4.071	5	5	6.181	28
2:1 vs. 1:0.5	19.20	27.82	-8.614	4.071	5	5	2.992	28
2:1 vs. 0.5:0.5	19.20	56.54	-37.33	4.071	5	5	12.97	28
2:1 vs. Donor	19.20	99.96	-80.75	4.071	5	5	28.05	28
2:1 vs. Cas9	19.20	99.86	-80.65	4.071	5	5	28.02	28
1:2 vs. 1:1	41.85	37.00	4.856	4.071	5	5	1.687	28
1:2 vs. 1:0.5	41.85	27.82	14.03	4.071	5	5	4.875	28
1:2 vs. 0.5:0.5	41.85	56.54	-14.68	4.071	5	5	5.101	28
1:2 vs. Donor	41.85	99.96	-58.10	4.071	5	5	20.19	28
1:2 vs. Cas9	41.85	99.86	-58.00	4.071	5	5	20.15	28
1:1 vs. 1:0.5	37.00	27.82	9.178	4.071	5	5	3.188	28
1:1 vs. 0.5:0.5	37.00	56.54	-19.54	4.071	5	5	6.788	28
1:1 vs. Donor	37.00	99.96	-62.96	4.071	5	5	21.87	28
1:1 vs. Cas9	37.00	99.86	-62.86	4.071	5	5	21.84	28
1:0.5 vs. 0.5:0.5	27.82	56.54	-28.72	4.071	5	5	9.977	28
1:0.5 vs. Donor	27.82	99.96	-72.14	4.071	5	5	25.06	28
1:0.5 vs. Cas9	27.82	99.86	-72.04	4.071	5	5	25.03	28
0.5:0.5 vs. Donor	56.54	99.96	-43.42	4.071	5	5	15.08	28
0.5:0.5 vs. Cas9	56.54	99.86	-43.32	4.071	5	5	15.05	28
Donor vs. Cas9	99.96	99.86	0.1000	4.071	5	5	0.03474	28

Number of families	1
Number of comparisons per family	21
Alpha	0.05

Hybrid transcripts

Tukey's multiple comparisons test	Mean diff.	95.00% CI of diff.	Below threshold?	Summary	Adjusted P Value
2:1 vs. 1:2	22.65	9.735 to 35.56	Yes	***	0.0001
2:1 vs. 1:1	17.79	4.879 to 30.71	Yes	**	0.0026
2:1 vs. 1:0.5	8.614	-4.299 to 21.53	No	ns	0.3712
2:1 vs. 0.5:0.5	37.33	24.42 to 50.25	Yes	****	<0.0001
2:1 vs. Donor	80.75	67.84 to 93.67	Yes	****	<0.0001
2:1 vs. Cas9	80.65	67.74 to 93.57	Yes	****	<0.0001
1:2 vs. 1:1	-4.856	-17.77 to 8.057	No	ns	0.8910
1:2 vs. 1:0.5	-14.03	-26.95 to -1.121	Yes	*	0.0265
1:2 vs. 0.5:0.5	14.68	1.771 to 27.60	Yes	*	0.0181
1:2 vs. Donor	58.10	45.19 to 71.02	Yes	****	<0.0001
1:2 vs. Cas9	58.00	45.09 to 70.92	Yes	****	<0.0001
1:1 vs. 1:0.5	-9.178	-22.09 to 3.735	No	ns	0.3003
1:1 vs. 0.5:0.5	19.54	6.627 to 32.45	Yes	***	0.0008
1:1 vs. Donor	62.96	50.05 to 75.87	Yes	****	<0.0001
1:1 vs. Cas9	62.86	49.95 to 75.77	Yes	****	<0.0001
1:0.5 vs. 0.5:0.5	28.72	15.80 to 41.63	Yes	****	<0.0001
1:0.5 vs. Donor	72.14	59.22 to 85.05	Yes	****	<0.0001
1:0.5 vs. Cas9	72.04	59.12 to 84.95	Yes	****	<0.0001
0.5:0.5 vs. Donor	43.42	30.51 to 56.33	Yes	****	<0.0001
0.5:0.5 vs. Cas9	43.32	30.41 to 56.23	Yes	****	<0.0001
Donor vs. Cas9	-0.1000	-13.01 to 12.81	No	ns	>0.9999

Test details	Mean 1	Mean 2	Mean diff.	SE of diff.	n1	n2	q	DF
2:1 vs. 1:2	80.80	58.15	22.65	4.071	5	5	7.868	28
2:1 vs. 1:1	80.80	63.00	17.79	4.071	5	5	6.181	28
2:1 vs. 1:0.5	80.80	72.18	8.614	4.071	5	5	2.992	28
2:1 vs. 0.5:0.5	80.80	43.46	37.33	4.071	5	5	12.97	28
2:1 vs. Donor	80.80	0.044	80.75	4.071	5	5	28.05	28
2:1 vs. Cas9	80.80	0.144	80.65	4.071	5	5	28.02	28
1:2 vs. 1:1	58.15	63.00	-4.856	4.071	5	5	1.687	28
1:2 vs. 1:0.5	58.15	72.18	-14.03	4.071	5	5	4.875	28
1:2 vs. 0.5:0.5	58.15	43.46	14.68	4.071	5	5	5.101	28
1:2 vs. Donor	58.15	0.044	58.10	4.071	5	5	20.19	28
1:2 vs. Cas9	58.15	0.144	58.00	4.071	5	5	20.15	28
1:1 vs. 1:0.5	63.00	72.18	-9.178	4.071	5	5	3.188	28
1:1 vs. 0.5:0.5	63.00	43.46	19.54	4.071	5	5	6.788	28
1:1 vs. Donor	63.00	0.044	62.96	4.071	5	5	21.87	28
1:1 vs. Cas9	63.00	0.144	62.86	4.071	5	5	21.84	28
1:0.5 vs. 0.5:0.5	72.18	43.46	28.72	4.071	5	5	9.977	28
1:0.5 vs. Donor	72.18	0.044	72.14	4.071	5	5	25.06	28
1:0.5 vs. Cas9	72.18	0.144	72.04	4.071	5	5	25.03	28
0.5:0.5 vs. Donor	43.46	0.044	43.42	4.071	5	5	15.08	28
0.5:0.5 vs. Cas9	43.46	0.144	43.32	4.071	5	5	15.05	28
Donor vs. Cas9	0.044	0.144	-0.1000	4.071	5	5	0.03474	28

Number of families	1
Number of comparisons per family	21
Alpha	0.05

Table S3. Statistical analysis of Figure 4F (whole liver Otc activity)

Tukey's multiple comparisons test	Mean diff.	95.00% CI of diff.	Below threshold?	Summary	Adjusted P Value
Spfash vs. Wild-type	-108.5	-128.8 to -88.26	Yes	****	<0.0001
Spfash vs. Cas9 only	-1.222	-21.49 to 19.04	No	ns	0.9998
Spfash vs. Donor only	-0.5983	-20.86 to 19.67	No	ns	>0.9999
Spfash vs. Dual	-6.989	-26.52 to 12.54	No	ns	0.8306
Wild-type vs. Cas9 only	107.3	87.04 to 127.6	Yes	****	<0.0001
Wild-type vs. Donor only	107.9	87.66 to 128.2	Yes	****	<0.0001
Wild-type vs. Dual	101.5	82.01 to 121.1	Yes	****	<0.0001
Cas9 only vs. Donor only	0.6233	-19.64 to 20.89	No	ns	>0.9999
Cas9 only vs. Dual	-5.768	-25.29 to 13.76	No	ns	0.9070
Donor only vs. Dual	-6.391	-25.92 to 13.14	No	ns	0.8710

Test details	Mean 1	Mean 2	Mean diff.	SE of diff.	n1	n2
Spf^{ash} vs. Wild-type	4.755	113.3	-108.5	6.920	6	6
Spf^{ash} vs. Cas9 only	4.755	5.977	-1.222	6.920	6	6
Spf^{ash} vs. Donor only	4.755	5.353	-0.5983	6.920	6	6
Spf^{ash} vs. Dual	4.755	11.74	-6.989	6.668	6	7
Wild-type vs. Cas9 only	113.3	5.977	107.3	6.920	6	6
Wild-type vs. Donor only	113.3	5.353	107.9	6.920	6	6
Wild-type vs. Dual	113.3	11.74	101.5	6.668	6	7
Cas9 only vs. Donor only	5.977	5.353	0.6233	6.920	6	6
Cas9 only vs. Dual	5.977	11.74	-5.768	6.668	6	7
Donor only vs. Dual	5.353	11.74	-6.391	6.668	6	7

Number of families	1
Number of comparisons per family	10
Alpha	0.05

Table S4: *In silico* sgRNA selection using Benchling (human targeting)

	Sequence (5'to 3')	PAM	Specificity score	Efficiency score
sgRNA1	CCTAAGCTACATAAAAATTAG	AAGAGT	43.8	43.6
sgRNA2	AAGTGAAGACTACTTAGACAG	TAGAGT	46.5	87.2
sgRNA3	TCTCAAATTCCTTGAAACAGA	GGGAGT	48.9	42.8

Table S5. Experimental details for studies using engrafted FRG mice

ID	Donor cells*	Cell number injected (million)	Capsid	Treatment	Human albumin level at time of injection (mg/mL)	Human albumin level at time of harvest (mg/mL)	Relevent Figure
Screening sgRNA efficiency in wild-type primary human hepatocytes							
253	#HUM4198	0.68	NP59	Cas9 only	0.3	0.4	Figure 5C
258	#HUM4198	0.68			1.7	1.5	
618	#HUM4198	0.52			0.2	0.6	
634	#HUM4198	0.52			0.4	1.5	
646	#HUM4198	0.52			0.4	0.7	
Evaluation of EGFP-containing donor vectors in wild-type primary human hepatocytes							
87	#HUM181141	0.44	NP59	Donor only (EGFP-no IRES)	0.9	1.2	Figure 5D-F
156	#HUM181141	0.44			0.5	1.4	
382	#HUM181141	0.56			0.4	2.0	
550	#HUM182411	0.48			1.2	3.5	
60	#HUM4198	0.8		Dual (EGFP-no IRES)	2.0	9.3	
61	#HUM4198	0.8			1.1	4.2	
191	#HUM181141	0.45			0.2	1.0	
208	#HUM181141	0.45			0.4	3.9	
606	#HUM181141	0.54			0.6	3.4	
620	#HUM181141	0.54			0.8	1.4	
501	Auxilary liver	0.54		Donor only (EMCV-EGFP)	0.5	0.6	
503	Auxilary liver	0.54			0.2	0.2	
504	Auxilary liver	0.54			0.5	1.5	
505	Auxilary liver	0.54			0.9	0.5	
167	#HUM4198	0.6		Dual (EMCV-EGFP)	0.4	3.1	
165	#HUM181141	0.45			0.6	2.5	
211	#HUM181141	0.45			0.2	1.7	
605	#HUM181141	0.54			0.9	2.2	
673	#HUM181141	0.8			0.6	2.8	
74	#HUM181141	0.44		Donor only (eIF4G-EGFP)	0.4	1.3	
83	#HUM181141	0.44			0.5	1.2	
289	Auxilary liver	0.9			0.7	2.3	
305	Auxilary liver	0.9			0.4	1.9	
166	#HUM181141	0.45		Dual (eIF4G-EGFP)	0.2	1.1	
202	#HUM181141	0.45			0.5	1.5	
355	#HUM182411	0.6			0.5	1.6	
477	#HUM4198	0.45			0.7	3.5	
699	#HUM181141	0.8			0.4	0.9	
Correction of OTC-deficient patient hepatocytes							
292	OTC patient (male) Exon 2 mutation: c.140 A>T, p. Asn47Ile	0.82	N/A	Untreated	0.2	2.6	Figure 7
527		0.82			0.2	2.9	
532		0.73			0.6	2.7	
555		0.82			0.3	3.4	
591		0.82			0.1	0.8	
500		0.8	SYD-12	Cas9 only	0.9	3.6	
506		0.8			0.5	2.7	
518		0.8			0.4	2.5	
557		0.82			0.3	2.0	
589		0.82			0.4	2.6	
482		0.82		Donor only (eIF4G-hOTC)	0.3	1.2	
515		0.82			0.5	1.1	
537		0.82			1.1	2.3	
575		0.82			0.6	1.9	
580		0.82			1.3	5.7	
582		0.82			1.2	3.4	
246		0.65		Dual (eIF4G-hOTC)	0.6	0.7	
253		0.65			1.0	1.5	
480		0.82			1.0	1.3	
481		0.82			1.2	1.4	
524		0.82			0.9	1.5	
536		0.82			0.5	0.4	
556		0.82			0.8	1.3	
581		0.82			0.4	0.6	

#HUM4198, #HUM181141 and #HUM182411 are wild-type primary human hepatocytes obtained from Lonza. Wild-type cells were also obtained from The Children's Hospital at Westmead om an auxilary liver. OTC-deficient primary human hepatocytes were obtained from a patient undergoing liver transplantation at The Children's Hospital at Westmead. All donor cells are male.

Table S6. Summary of reads at the Cas-9 cleavage site of the murine *Otc* locus

Sequencing strategy*	read_id	classification	Host contig	mapping_start	mapping_end	overlap_start	overlap_end	mapq	identity	indel_count	indel_details	class assigned
WGS	058c688c-955a-4069-b00b-3eec154046c5	single_junction	chrX	10114775	10121772	10121747	10121772	60	0.985	0	NA	ITR only
WGS	1a4dca7d-5567-4805-a24f-a5a14a92c85a	indel	chrX	10109608	10122616	10121747	10121847	60	0.9574	3	del:3@10121793-10121795;del:1@10121797-10121797;del:1@10121800-10121800	indel
WGS	28fca91a-3b61-4175-bb77-815501900b8a	single_junction	chrX	10118172	10121806	10121747	10121806	60	0.9819	1	del:4@10121793-10121796	ITR only
WGS	2a78a26d-3454-48a6-b674-1568702b6bbd	double_junction	chrX	10118589	10128165	10121747	10121847	60	0.9816	2	ins:1@10121798;ins:93@10121800	ITR only
WGS	2c213eb0-8409-4588-902d-199f53b6d6ba	indel	chrX	10118498	10128726	10121747	10121847	60	0.9886	1	del:3@10121797-10121799	indel
WGS	2f5d323a-a813-4f42-8308-de8f103b18df	single_junction	chrX	10118537	10121798	10121747	10121798	60	0.993	0	NA	Cas9 only
WGS	3c92c559-63fc-48bd-9470-64779fe12156	indel	chrX	10121457	10126023	10121747	10121847	60	0.9969	1	del:2@10121795-10121796	indel
WGS	4252cdc3-51a2-47b7-a180-f7b774077ac5	unmodified	chrX	10114668	10136273	10121747	10121847	60	0.9759	0	NA	unmodified
WGS	44ac8e95-9204-4b24-83cb-1f70ed923c3d	unmodified	chrX	10119200	10135941	10121747	10121847	60	0.9936	0	NA	unmodified
WGS	66c626e8-eb6d-4155-bdd2-8ae42d3cc84c	unmodified	chrX	10121195	10131411	10121747	10121847	60	0.986	0	NA	unmodified
WGS	82a0084c-07a4-47de-9c64-a6c58b178236	single_junction	chrX	10120255	10121798	10121747	10121798	60	0.9916	0	NA	Cas9 only
WGS	9ce04493-5da0-4d02-8a2b-adf502452acd	unmodified	chrX	10117706	10124073	10121747	10121847	60	0.9874	0	NA	unmodified
WGS	b9f2da27-2137-4bf3-8dec-b7d6c70404b2	single_junction	chrX	10119693	10121783	10121747	10121783	60	0.9852	0	NA	HITI event
WGS	c51a40aa-7bc7-412f-b119-5b6bb995fcb4	single_junction	chrX	10118819	10121797	10121747	10121797	60	0.9826	0	NA	concatemer
WGS	c8ec8b11-5189-41be-9f50-99289c0a5853	unmodified	chrX	10121669	10122049	10121747	10121847	60	0.9869	0	NA	unmodified
WGS	c9d3ba09-c89b-4594-adee-751355f934a0	single_junction	chrX	10121845	10122469	10121845	10121847	60	0.9968	0	NA	ITR only
WGS	da8b0b5b-4115-4fc2-bbe7-aabc5469d144	single_junction	chrX	10121799	10126030	10121799	10121847	60	0.9854	0	NA	concatemer
WGS	ec94f8f7-daef-4889-864d-9937c3deb117	other	chrX	10121838	10127149	10121838	10121847	60	0.9617	0	NA	downstream of cut site, not included in analysis
AS	022cfe96-73a0-4faa-8519-5c6aab1a658a	unmodified	chrX	10051107	10123312	10121747	10121847	60	0.9777	0	NA	unmodified
AS	0bae4216-eb6f-4e0e-89ce-4a517621ec04	double_junction	chrX	10099710	10129649	10121747	10121847	60	0.7733	1	ins:408@10121797	concatemer
AS	23d4f159-5694-4fcd-985e-898a463a41bc	indel	chrX	10121755	10146410	10121755	10121847	60	0.9834	1	ins:1@10121798	indel
AS	26f1df09-933a-4665-aa09-51f7016b03c2	unmodified	chrX	10114923	10147152	10121747	10121847	60	0.9636	0	NA	unmodified
AS	3a9c63cb-b83d-458c-b25a-66bc65a64856	single_junction	chrX	10097065	10121797	10121747	10121797	60	0.9743	0	NA	concatemer
AS	455c8907-cacd-48a6-8b94-d984dcbf9cd5	single_junction	chrX	10121798	10136889	10121798	10121847	60	0.9652	0	NA	ITR only
AS	535b21db-652d-4b99-b36b-0aab3a251e7b	unmodified	chrX	10120898	10129853	10121747	10121847	60	0.9837	0	NA	unmodified
AS	982a0e59-da14-4afc-ab37-01c709ba602e	indel	chrX	10120447	10122777	10121747	10121847	60	0.9854	2	del:1@10121793-10121793;del:1@10121797-10121797	indel
AS	9bd12fde-db60-4036-9717-62fb3fb878a7	indel	chrX	10117760	10123606	10121747	10121847	60	0.9867	2	del:1@10121793-10121793;ins:1@10121800	indel
AS	a447265f-4e53-424b-afec-97afee86baef	single_junction	chrX	10121797	10126255	10121797	10121847	60	0.9605	0	NA	concatemer
AS	b9bc71fb-0f60-41ce-891e-741e33f592de	single_junction	chrX	10121800	10122076	10121800	10121847	60	0.9819	0	NA	cut site not detected, not included in analysis
AS	d6991770-8b9c-4ec6-a7c9-691f5109a0a4	single_junction	chrX	10121798	10159968	10121798	10121847	60	0.9766	0	NA	HITI event
AS	f3a77775-c158-4fd9-a202-b9ff83aaabe0	unmodified	chrX	10121797	10126255	10121797	10121847	60	0.975	0	NA	unmodified
AS	f6e6a744-ce1a-4ba9-8263-debb97bc1844	indel	chrX	10105026	10134004	10121747	10121847	60	0.993	1	del:5@10121792-10121796	indel
AS	f8609b57-d5ea-4755-bda2-238e4823d9e5	indel	chrX	10121115	10128075	10121747	10121847	60	0.9843	1	del:1@10121793-10121793	indel

*WGS, whole genome sequencing; AS, WGS with adaptive sampling

Table S7. Primers used in this study

A

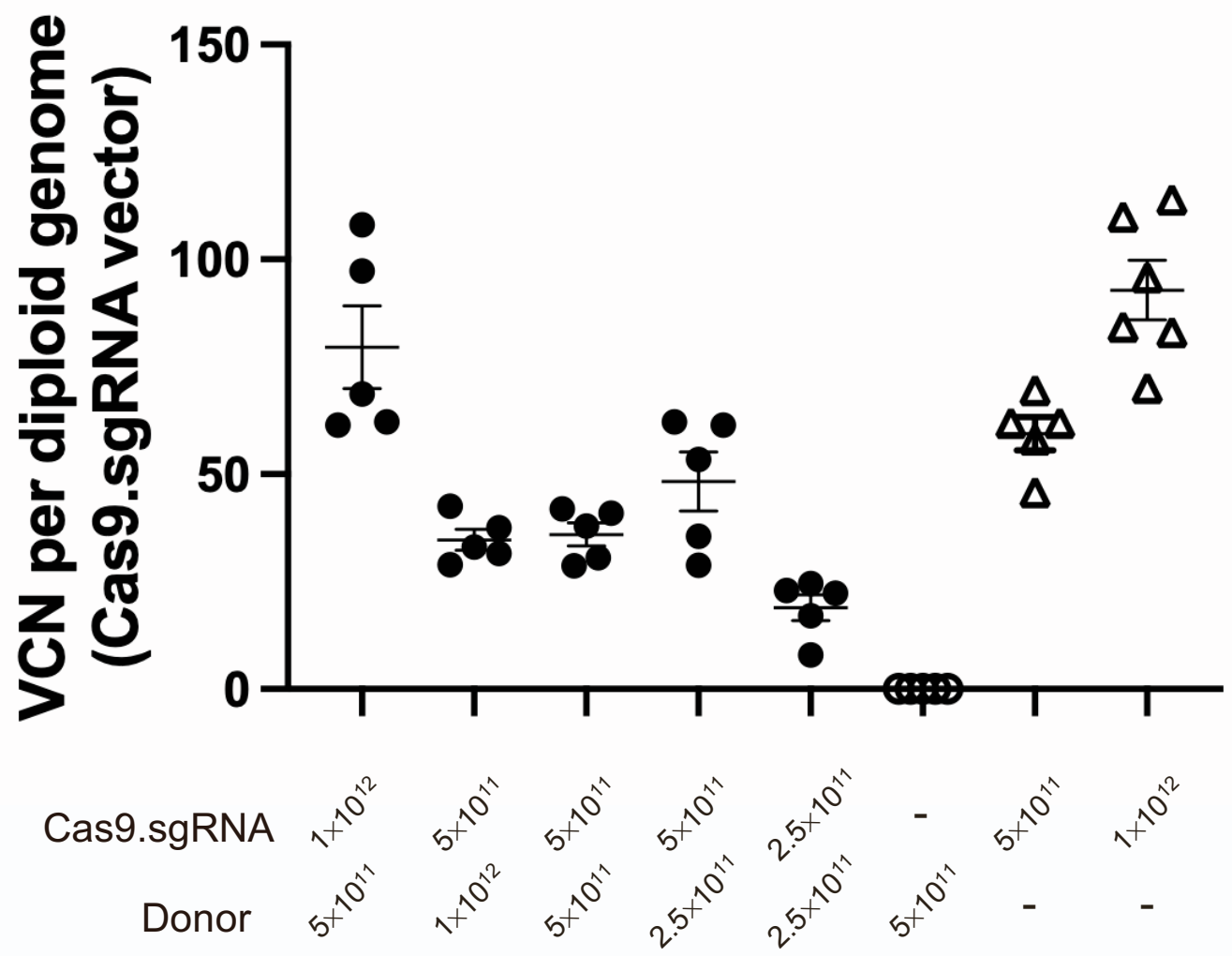

B

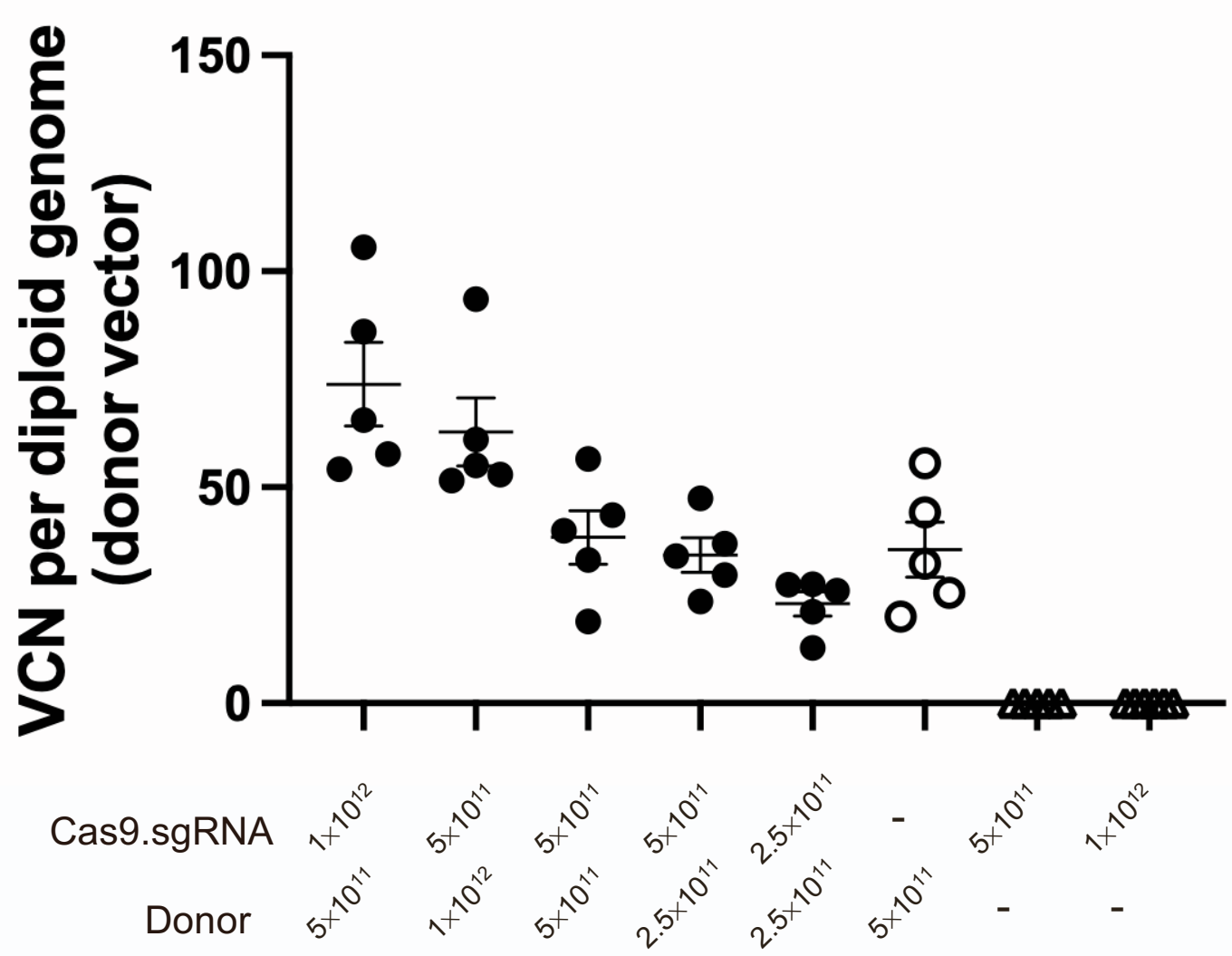

Figure S1.
Quantification of vector copy number (VCN) following dual AAV delivery in C57Bl6 mice. Vector copy number of (A) the SaCas9 and (B) EGFP transgenes per diploid genome in genomic DNA isolated from whole liver were determined by digital PCR (n=5 per treatment group, mean ± SEM are indicated). Doses of the SaCas9.sgRNA and EGFP-containing donor vectors are shown below each graph.

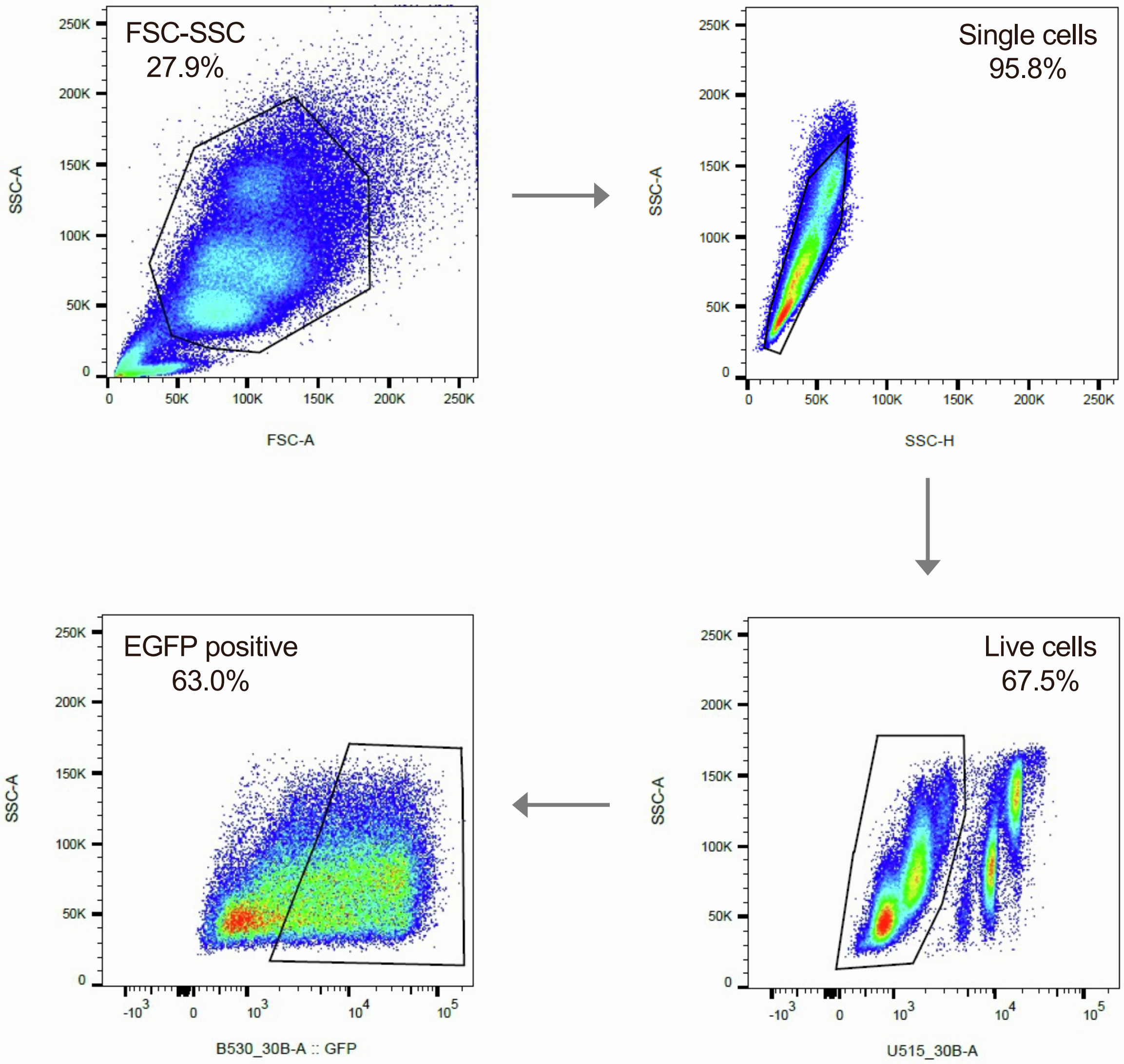

Figure S2.
Analysis of EGFP expression in the murine liver. Representative FACS profiles with gating strategy showing the percentage of EGFP positive hepatocytes 4 weeks after dual AAV delivery with 5×10^{11} vg of each vector. FSC, forward scatter; SSC, side scatter.

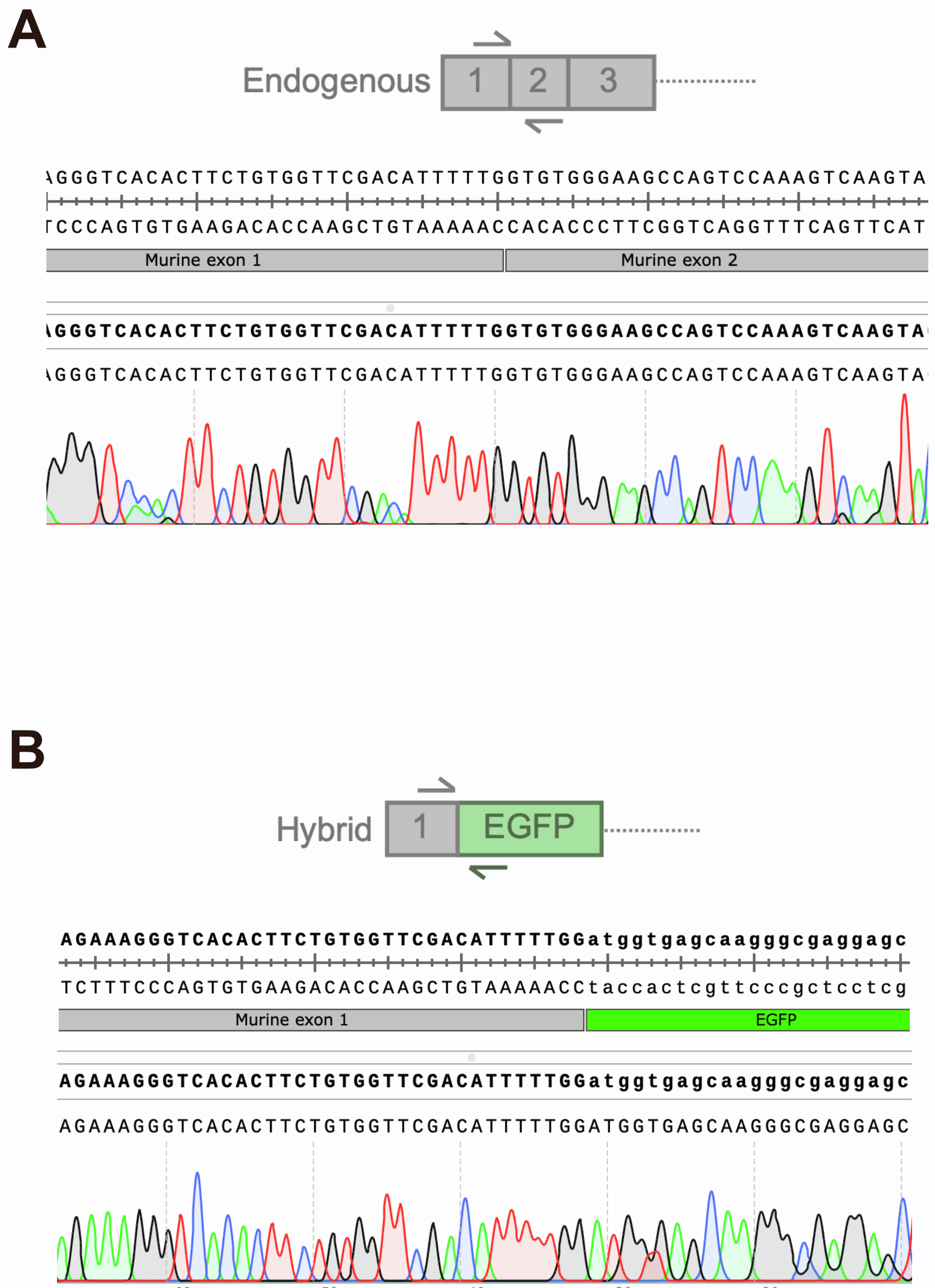

Figure S3.
Identification of transcripts originating from the *Otc* promoter. (A) endogenous transcripts and (B) hybrid transcripts between murine *Otc* exon 1 and EGFP. Junctions were confirmed in cDNA by using PCR primers that span the predicted junction followed by Sanger sequencing of the amplicons.

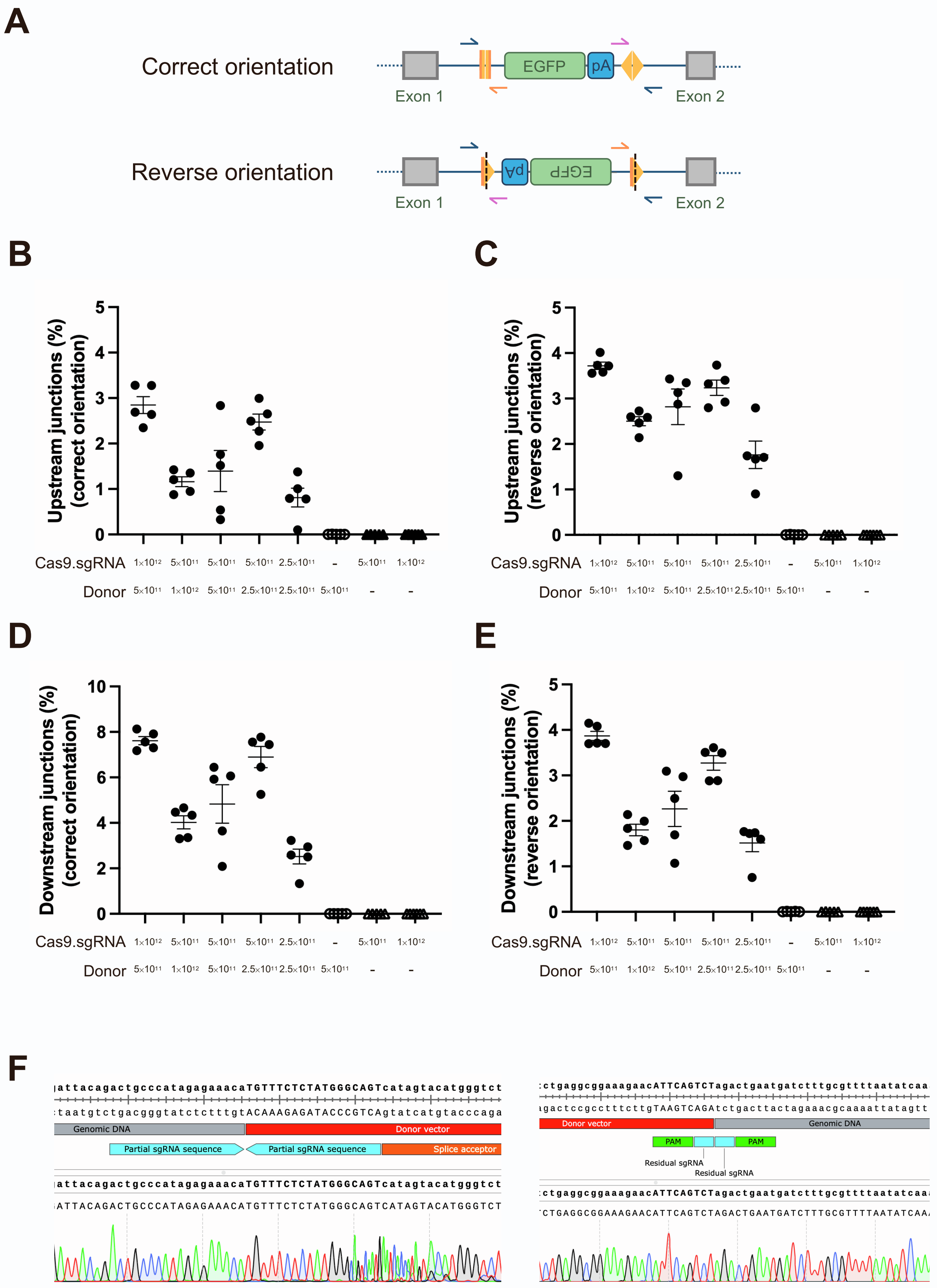
A
Correct orientation
EGFP
pA
Exon 1
Exon 2
Reverse orientation
EGFP
pA
Exon 1
Exon 2
B
Upstream junctions (%)
(correct orientation)
0
1
2
3
4
5
Cas9.sgRNA 1×10^12 5×10^11 5×10^11 5×10^11 2.5×10^11 - 5×10^11 1×10^12
Donor 5×10^11 1×10^12 5×10^11 2.5×10^11 2.5×10^11 5×10^11 - -
C
Upstream junctions (%)
(reverse orientation)
0
1
2
3
4
5
Cas9.sgRNA 1×10^12 5×10^11 5×10^11 5×10^11 2.5×10^11 - 5×10^11 1×10^12
Donor 5×10^11 1×10^12 5×10^11 2.5×10^11 2.5×10^11 5×10^11 - -
D
Downstream junctions (%)
(correct orientation)
0
2
4
6
8
10
Cas9.sgRNA 1×10^12 5×10^11 5×10^11 5×10^11 2.5×10^11 - 5×10^11 1×10^12
Donor 5×10^11 1×10^12 5×10^11 2.5×10^11 2.5×10^11 5×10^11 - -
E
Downstream junctions (%)
(reverse orientation)
0
1
2
3
4
5
Cas9.sgRNA 1×10^12 5×10^11 5×10^11 5×10^11 2.5×10^11 - 5×10^11 1×10^12
Donor 5×10^11 1×10^12 5×10^11 2.5×10^11 2.5×10^11 5×10^11 - -
F
attacagactgcccatagagaaacaTGTTTCTCTATGGGCAGTcatagtacatgggtct
taatgtctgacgggtatctctttgtACAAAGAGATACCCGTCAgtatcatgtacccaga
Genomic DNA
Donor vector
Partial sgRNA sequence
Partial sgRNA sequence
Splice acceptor
attacagactgcccatagagaaacaTGTTTCTCTATGGGCAGTcatagtacatgggtct
ATTACAGACTGCCCATAGAGAAACATGTTTCTCTATGGGCAGTCATAGTACATGGGTCT
ctgaggcggaaagaacATTCAGTCTagactgaatgatctttgcgttttaatatcaa
gactccgcctttcttgTAAGTCAGAtctgacttactagaaacgcaaaattatagtt
Donor vector
Genomic DNA
PAM
PAM
Residual sgRNA
Residual sgRNA
ctgaggcggaaagaacATTCAGTCTagactgaatgatctttgcgttttaatatcaa
CTGAGGCGGAAAGAACATTCAGTCTAGACTGAATGATCTTTGCGTTTTAATATCAA

Figure S4.

Quantification of precise HITI junctions between the murine genome and integrated EGFP donor. (A) Diagrammatic representation of predicted upstream and downstream integration events with locations of primers indicated. pA, bovine growth hormone polyadenylation signal. Yellow arrow heads indicate the location of the sgRNA sequence and orange rectangles the protospacer adjacent motif (PAM). (B-E) Genomic DNA was isolated from whole liver 4 weeks after vector delivery and predicted junctions into the target locus were quantified by digital PCR (n=5 per treatment group, mean ± SEM are indicated). (F) The identify of upstream and downstream junctions were confirmed by PCR using primers that span the predicted junction followed by Sanger sequencing of the amplicons.

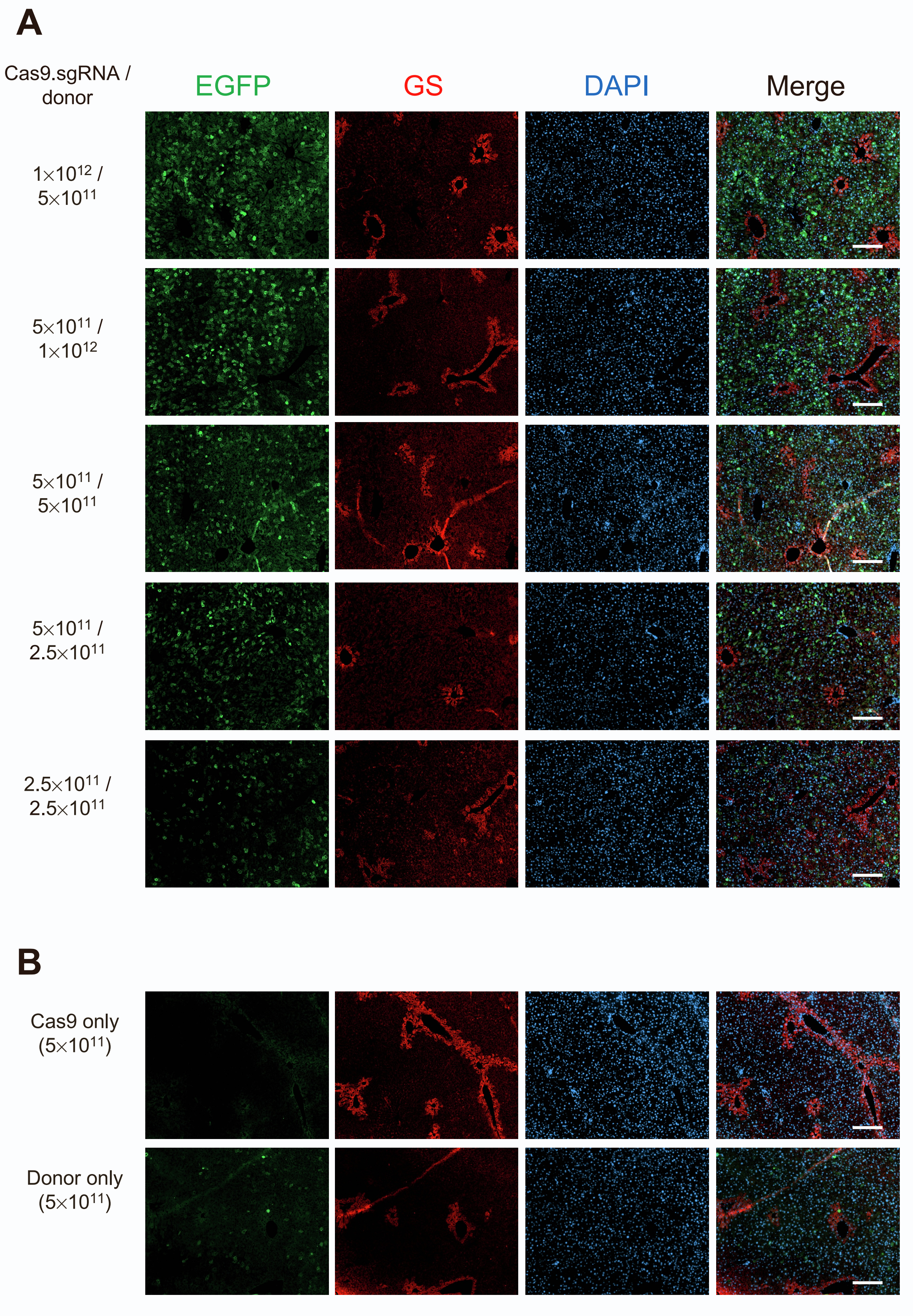
A
Cas9.sgRNA / donor
EGFP
GS
DAPI
Merge
1×10¹² / 5×10¹¹
5×10¹¹ / 1×10¹²
5×10¹¹ / 5×10¹¹
5×10¹¹ / 2.5×10¹¹
2.5×10¹¹ / 2.5×10¹¹
B
Cas9 only (5×10¹¹)
Donor only (5×10¹¹)

Figure S5.

Physiological expression patterns following targeted integration are observed over a range of vector doses. (A) Reporter gene expression following integration of the donor template in murine hepatocytes shows metabolic zonation and (B) single vector treated controls. Sections were labelled with antibodies against the EGFP (green) and glutamine synthetase (GS, red) proteins and nuclei were counter-stained with DAPI (blue). Doses of the SaCas9.sgRNA and EGFP-containing donor vectors are shown to the right of the images. Scale bars = 200 μm.

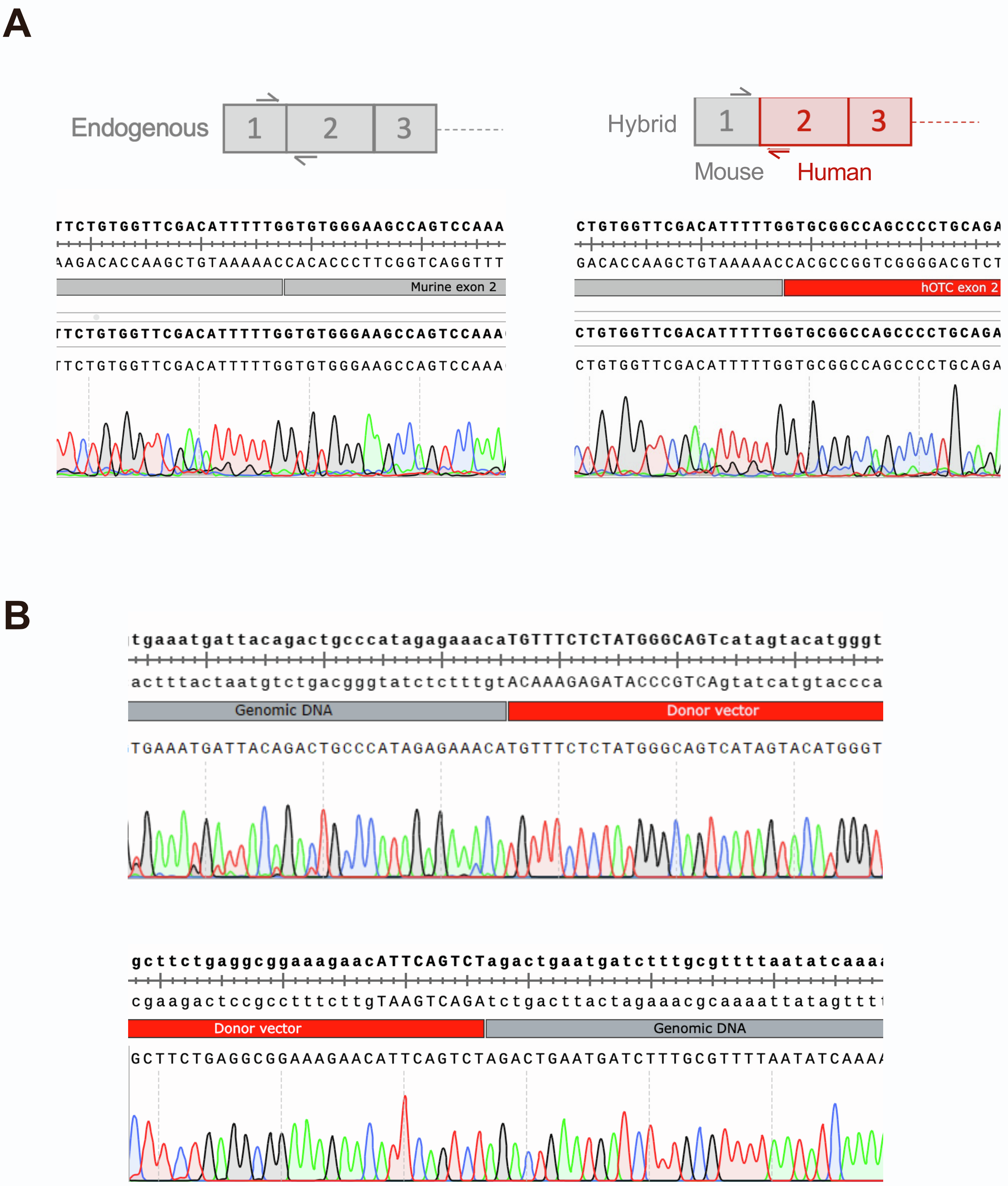

Figure S6.
Identification of predicted upstream and downstream HITI junctions between the murine genome and integrated OTC donor in (A) transcripts and (B) genomic DNA. Junctions were confirmed by PCR using primers that span the predicted junction followed by Sanger sequencing of the amplicons.

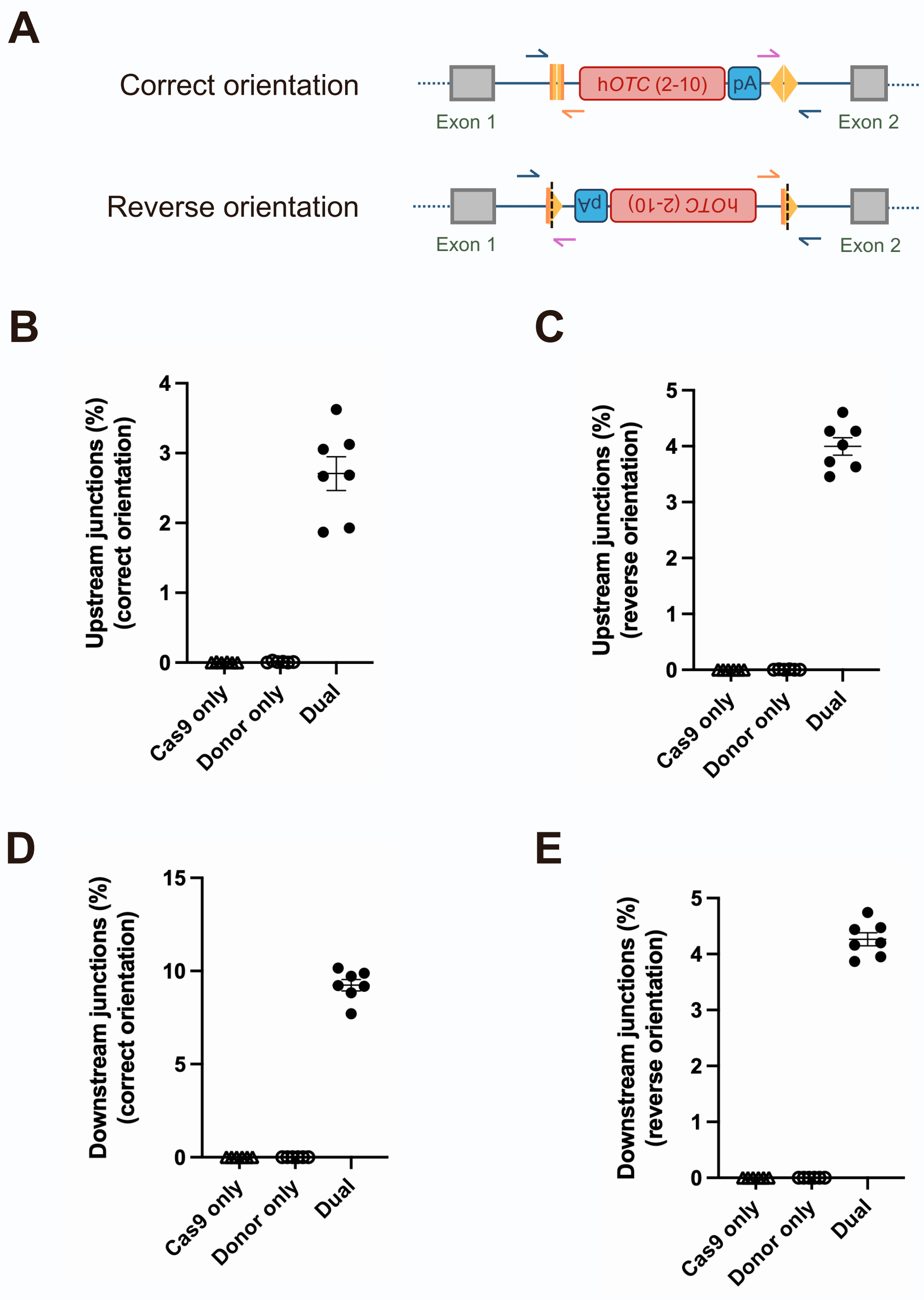

Figure S7.

Quantification of precise HITI junctions between the murine genome and integrated OTC donor. (A) Diagrammatic representation of predicted upstream and downstream integration events with locations of primers indicated. pA, bovine growth hormone polyadenylation signal. Yellow arrow heads indicate the location of the sgRNA sequence and orange rectangles the protospacer adjacent motif (PAM). (B-E) Genomic DNA was isolated from whole liver 8 weeks after vector delivery and predicted junctions into the target locus were quantified by digital PCR (n=6-7 per treatment group, mean ± SEM are indicated).

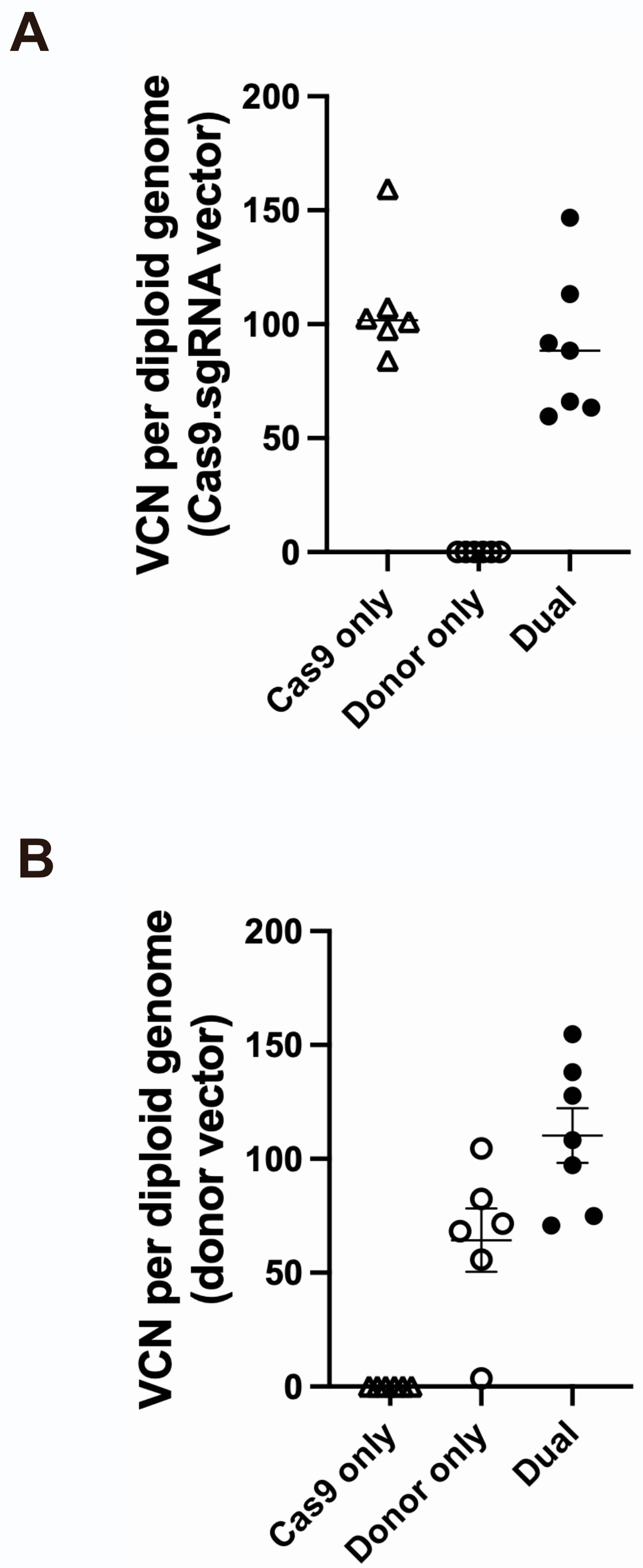

Figure S8.
Quantification of vector copy number (VCN) following dual AAV delivery in *Spf*^{ash} mice. Vector copy number of (A) the SaCas9 and (B) *OTC* transgenes per diploid genome in genomic DNA isolated from whole liver 8 weeks after vector delivery were determined by digital PCR (n=6-7 per treatment group, mean ± SEM are indicated). Animals receiving either the donor or editing reagents were included as controls.

A

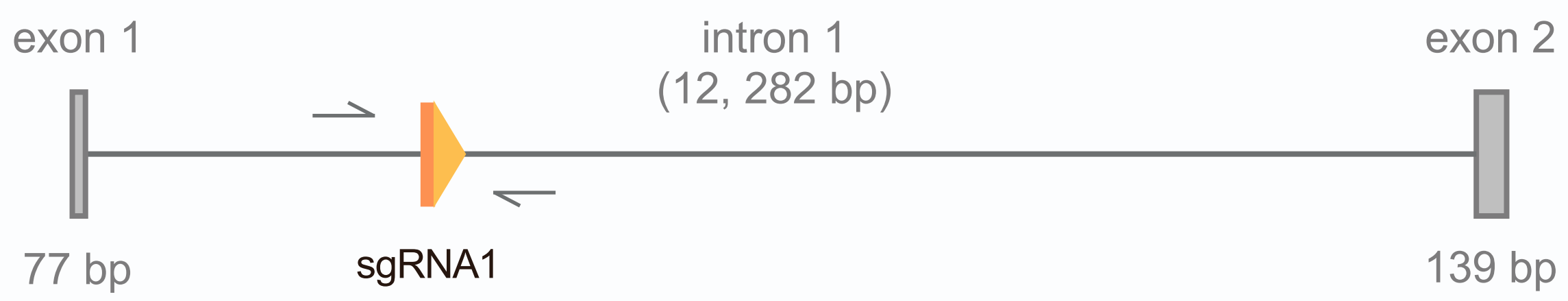

B

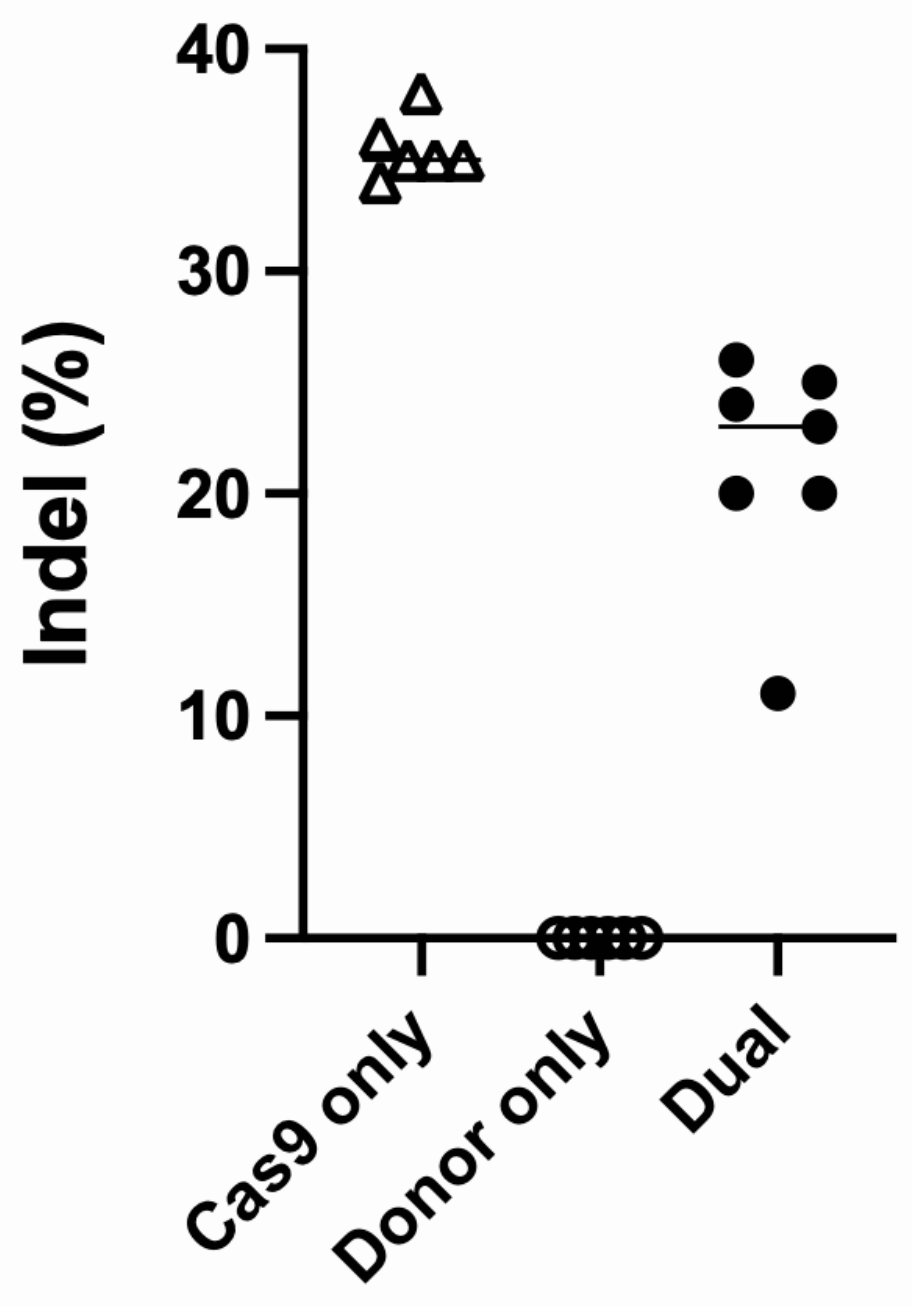

Figure S9.
Quantification of indel frequency following targeted integration. (A) Location of primers and sgRNA (rectangle) and PAM (triangle) sequences in the first intron of the murine Otc locus. (B) Genomic DNA was isolated from whole liver 8 weeks after dual vector delivery and the percentage of indels determined by PCR using primers that span the predicted SaCas9 cleavage site followed by Sanger sequencing of the amplicons (n=6-7 per treatment group, mean ± SEM are indicated). Animals receiving either the donor or editing reagents were included as controls.

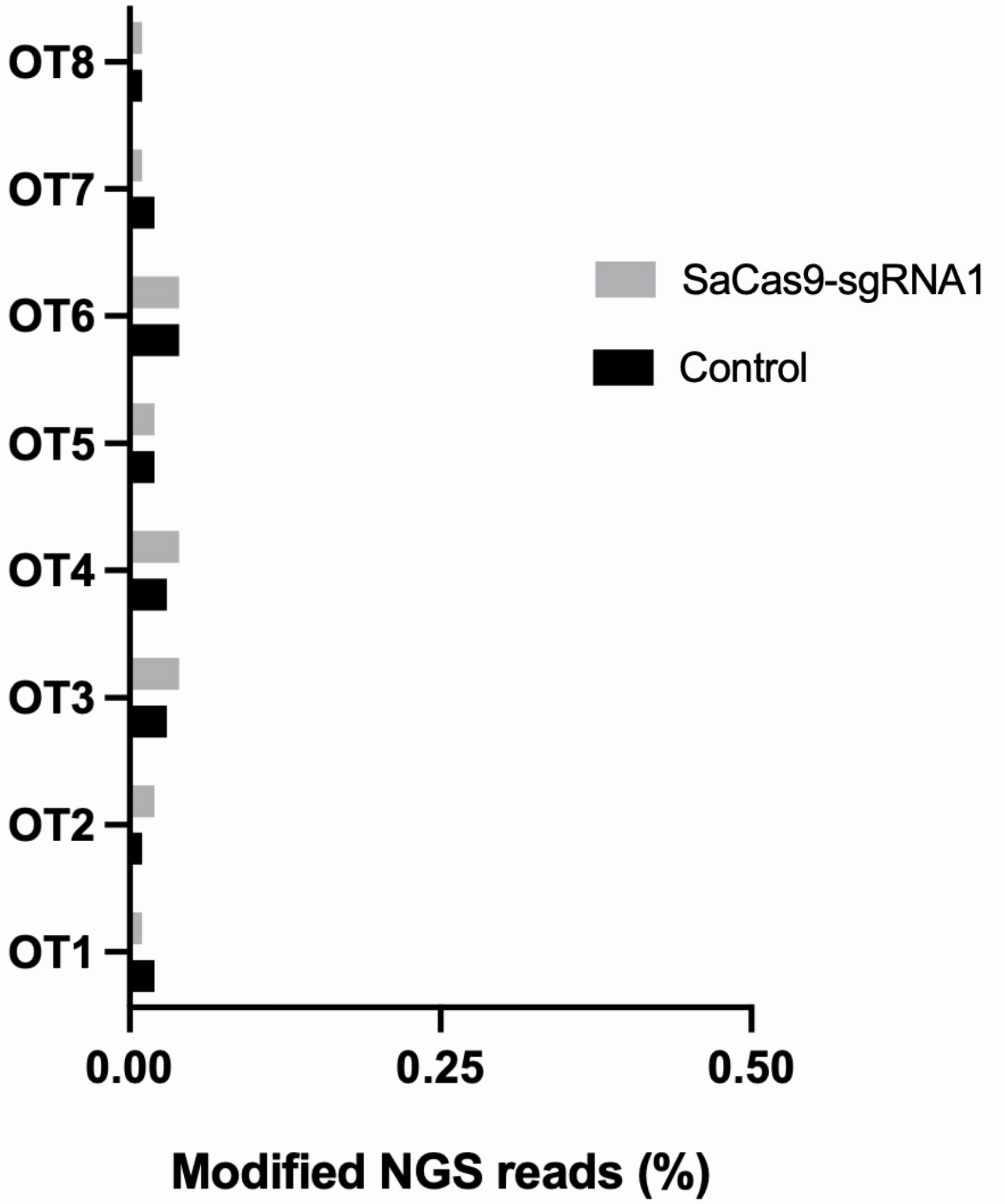

Figure S10.
Analysis of predicted off-target sites in the human genome. Amplicons from both a treated and control FRG mice, engrafted with primary human hepatocytes, were mixed in equimolar ratios and NGS sequencing using a 2 × 150 paired-end configuration (PE150) were performed. Modified reads in both the treated and control samples are expressed as a percentage to the total. Off-target sites were identified using GT-Scan (https://gt-scan.csiro.au/). All values were between 0.01 and 0.04% of the total reads.

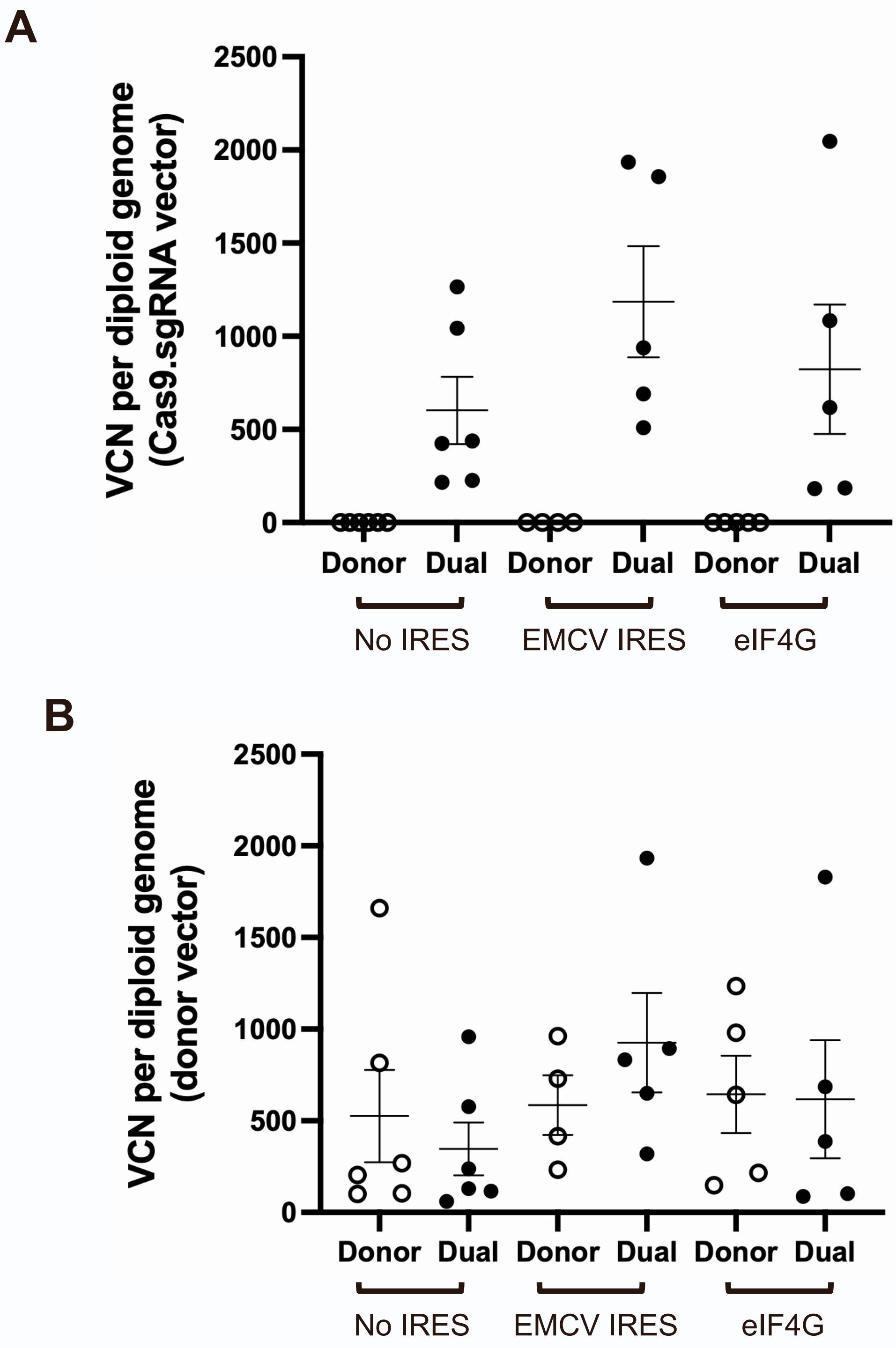

Figure S11.

Quantification of vector copy number (VCN) following dual AAV delivery in engrafted FRG mice. Vector copy number of (A) the SaCas9 and (B) EGFP transgenes per diploid genome in genomic DNA isolated from whole liver were determined by digital PCR (n=4-6 per treatment group). Mean ± SEM for each of the three donor configurations are indicated and animals receiving only the donor vectors were included as controls. All vectors were packaged in the NP59 capsid.

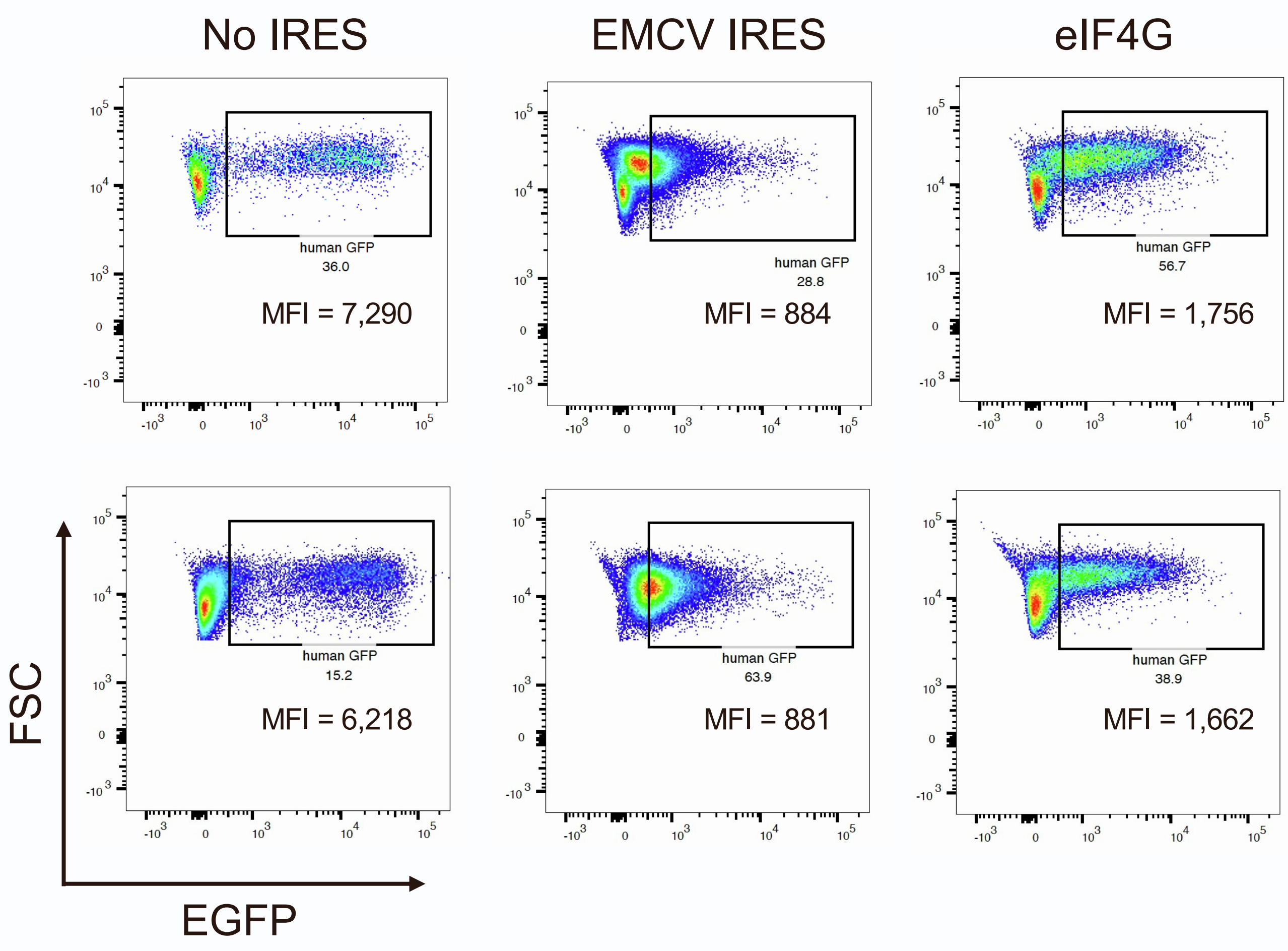

Figure S12.
Influence of the inclusion of an IRES element on gene expression levels. Fluorescent activated cell sorting (FACS) analysis of perfused chimeric mouse-human livers following dual AAV delivery to determine the percentage of EGFP positive human hepatocytes. The mean fluorescence intensity (MFI) for each of the three donor vector configurations are shown (n=2 animals per treatment group shown).

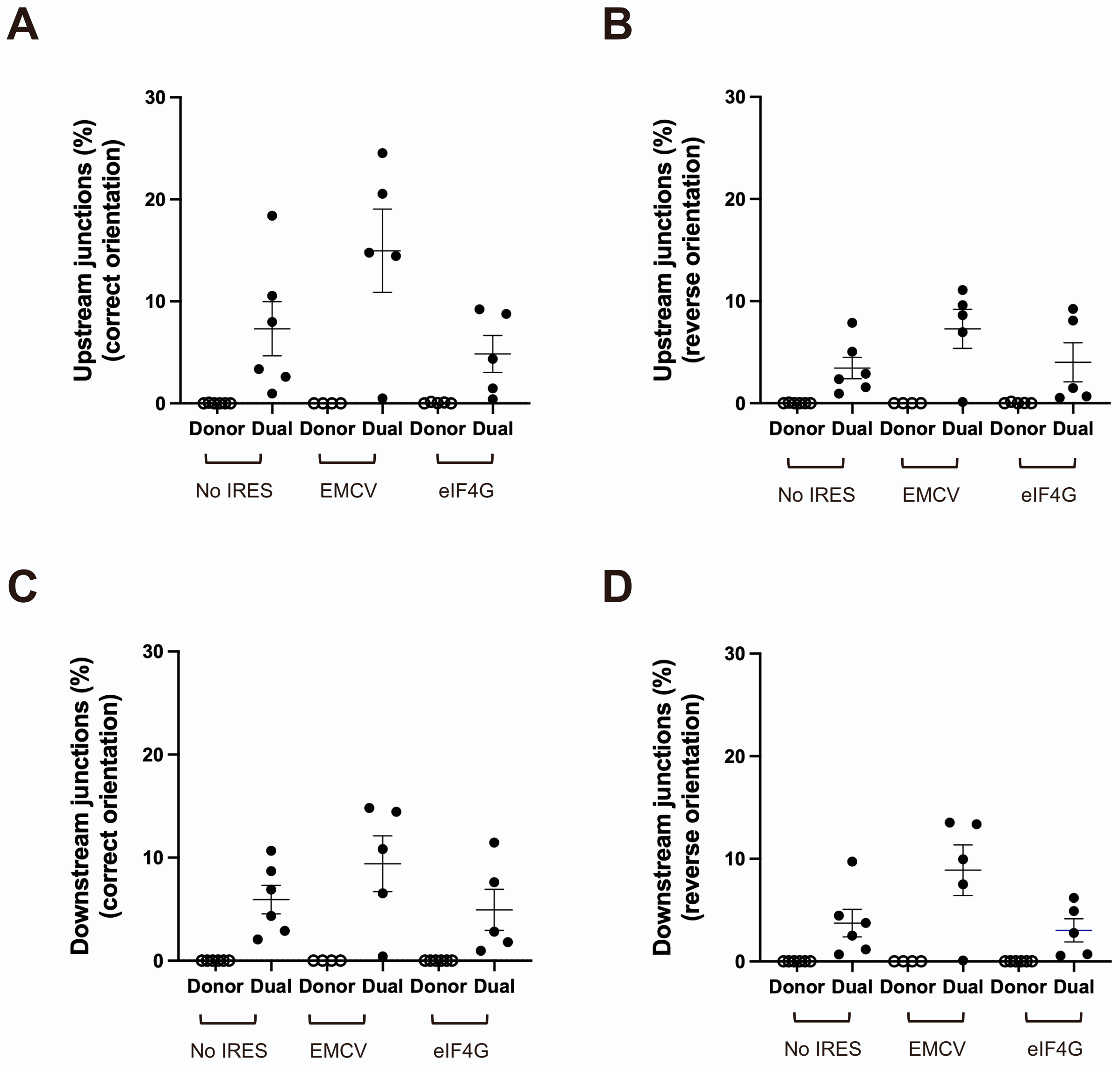

Figure S13.

Quantification of precise HITI junctions between the human genome and integrated EGFP-containing donor cassettes. Genomic DNA was isolated from whole liver 6 weeks after vector delivery and predicted junctions into the target OTC locus were quantified by digital PCR (A) upstream junctions in the correct orientation, (B) upstream junctions in the reverse orientation, (C) downstream junctions in the correct orientation, (D) downstream stream junctions in the reverse orientation (n=5 or 6 per treatment group, mean ± SEM are indicated).

A

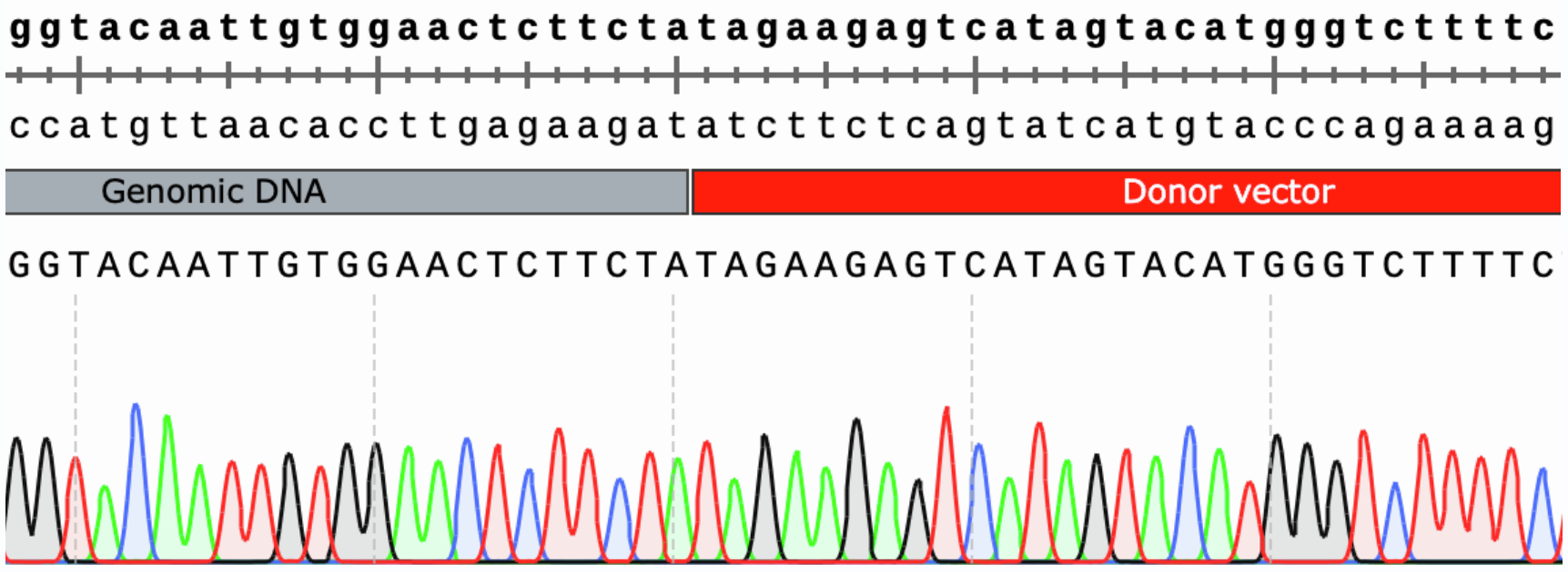

B

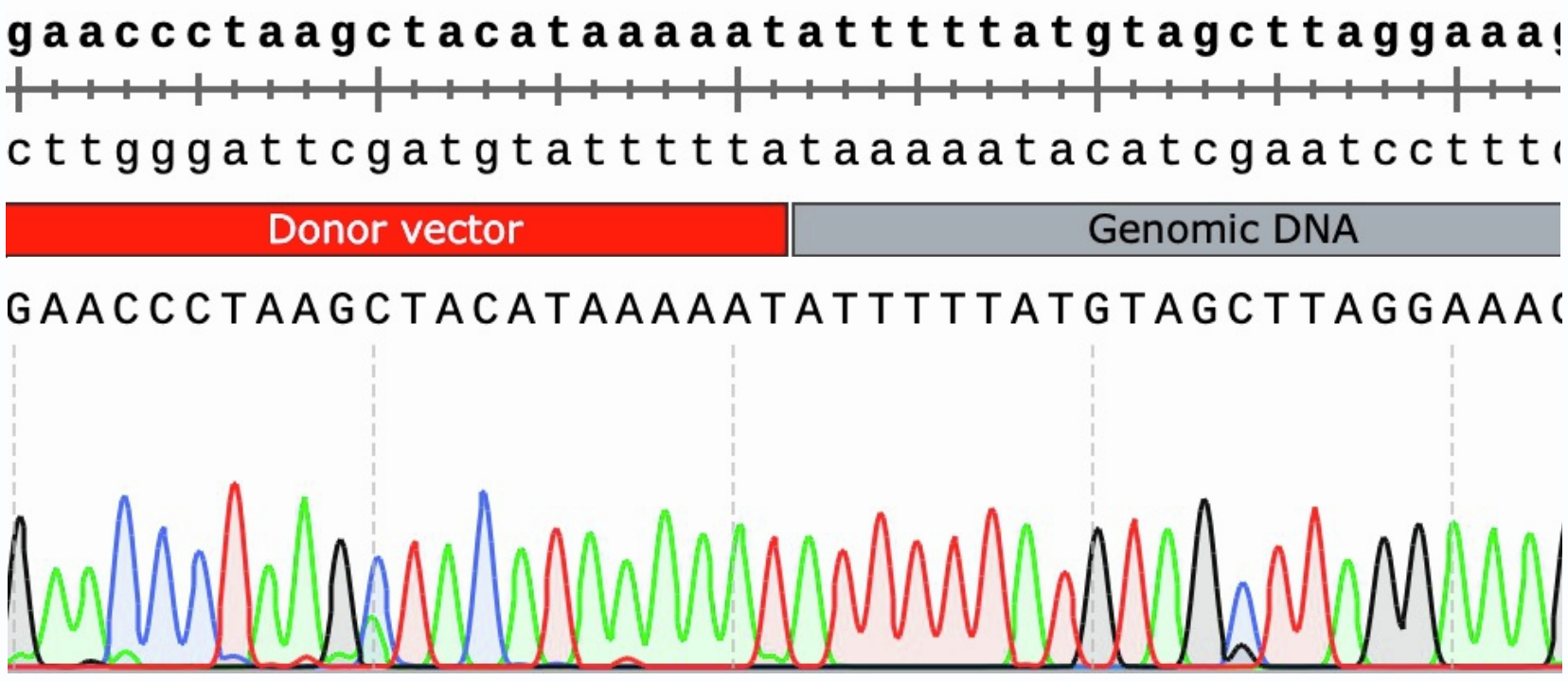

Figure S14.

Identification of predicted HITI junctions between the human genome and integrated EGFP donor. (A) Upstream and (B) downstream junctions were confirmed by PCR using primers that span the predicted junction followed by Sanger sequencing of the amplicons.

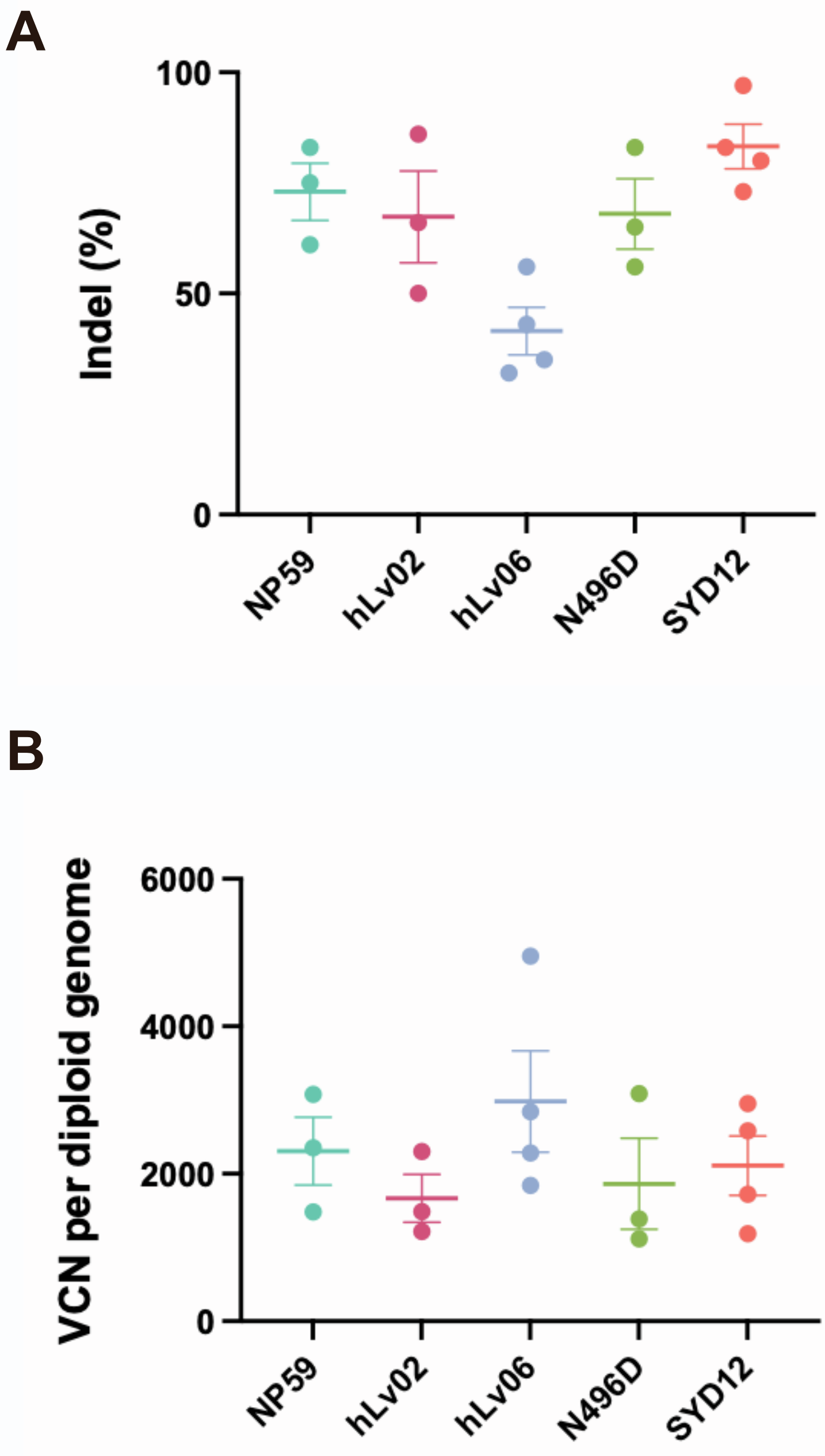

Figure S15.
Comparison of editing efficiency using selected human liver-tropic capsids. (A) The percentage of indels in genomic DNA from chimeric mouse-human livers 6 weeks after vector delivery was determined by PCR using primers that span the predicted SaCas9 cleavage site followed by Sanger sequencing of the amplicons. (B) Quantification of vector copy number (VCN) by digital PCR. (n=3-4 per capsid, mean ± SEM are shown).

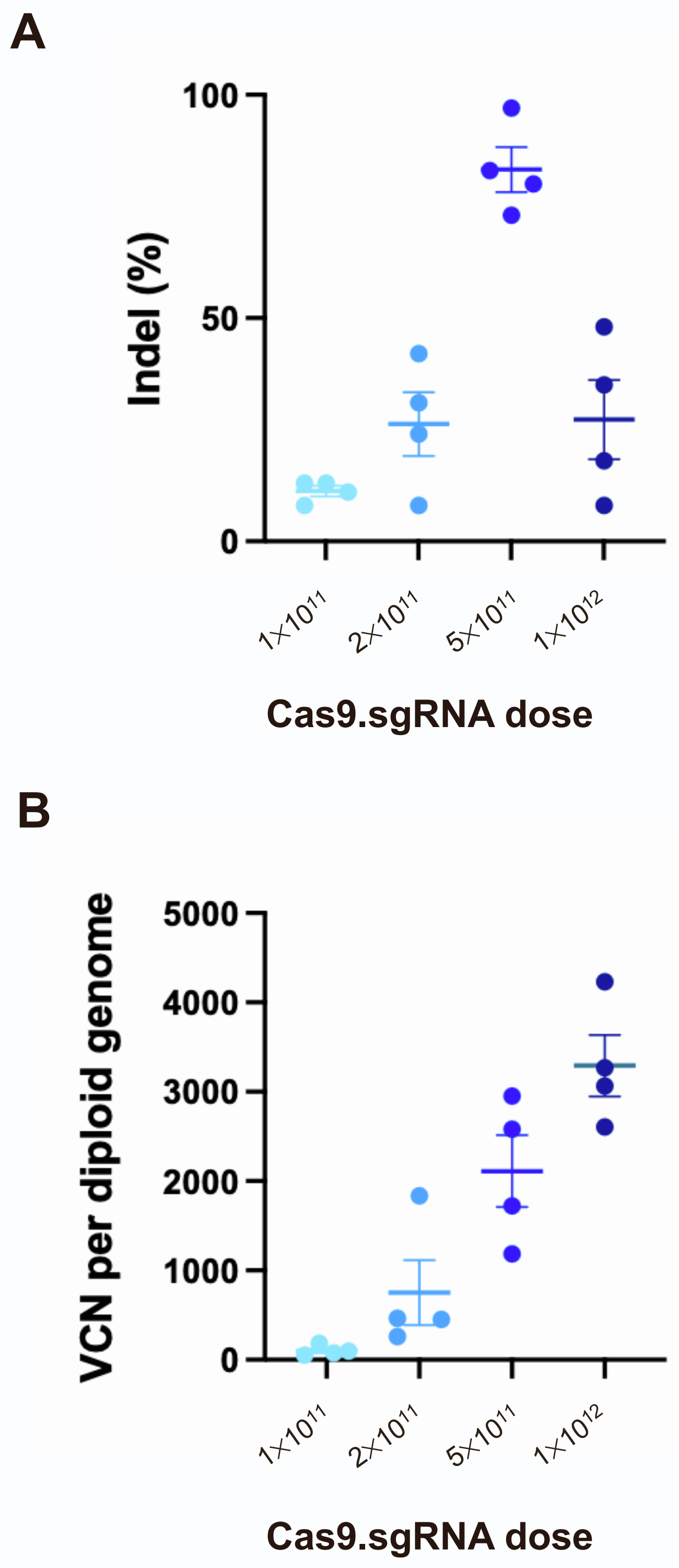

Figure S16. Determination of optimal vector dose for *in vivo* editing using the SYD12 capsid. (A) The percentage of indels in genomic DNA from chimeric mouse-human livers 6 weeks after vector delivery was determined by PCR using primers that span the predicted SaCas9 cleavage site followed by Sanger sequencing of the amplicons. (B) Quantification of vector copy number (VCN) by digital PCR. (n=4 per treatment group, mean ± SEM are shown).

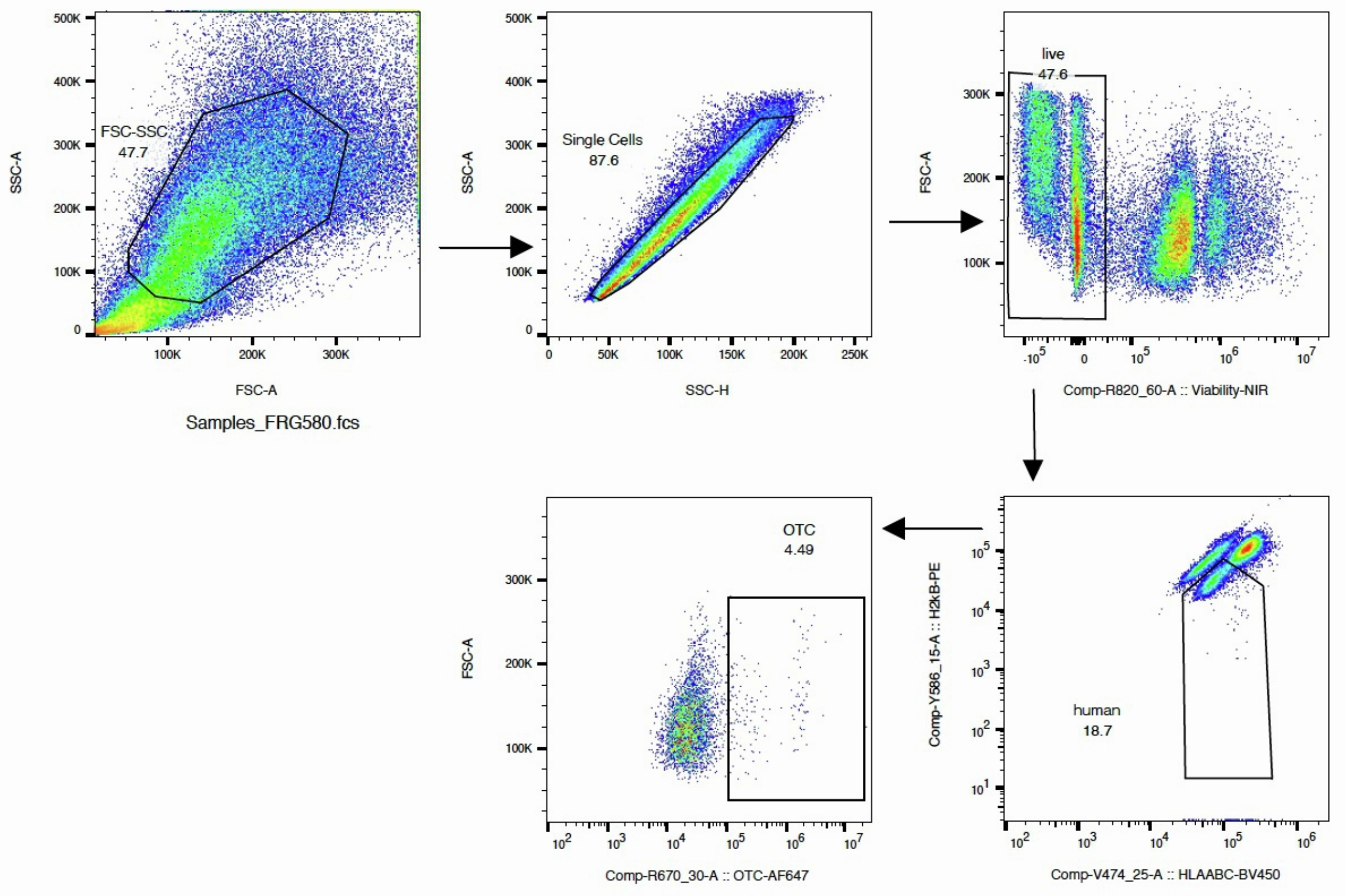

Figure S17.

Gating strategy used for to determine the percentage of OTC positive primary human hepatocytes in chimeric mouse-human livers in FRG mice using FACS analysis. Representative FACS profiles with gating strategy showing the percentage of human OTC positive hepatocytes 6 weeks after dual AAV delivery of 5×10^{11} vg of the editing and 1×10^{11} vg of the donor vector.

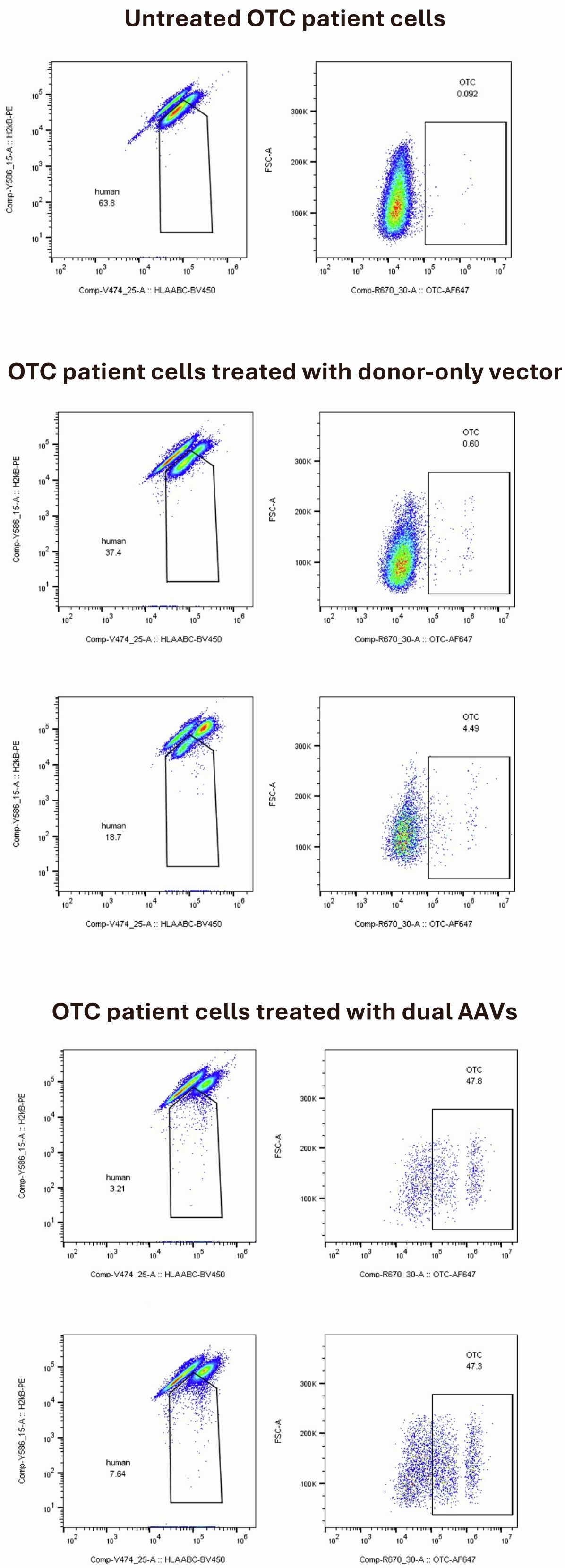

Figure S18.

Restoration of OTC expression in patient-derived primary human hepatocytes *in vivo* following dual vector delivery. The percentage of OTC positive hepatocytes from engrafted FRG animals receiving 5×10^{11} vg/mouse of the AAV vector containing the editing reagents and 1×10^{11} vg/mouse of the AAV donor packaged in the SYD12 capsid. Untreated and mice treated with only the donor vector are shown as controls.

A

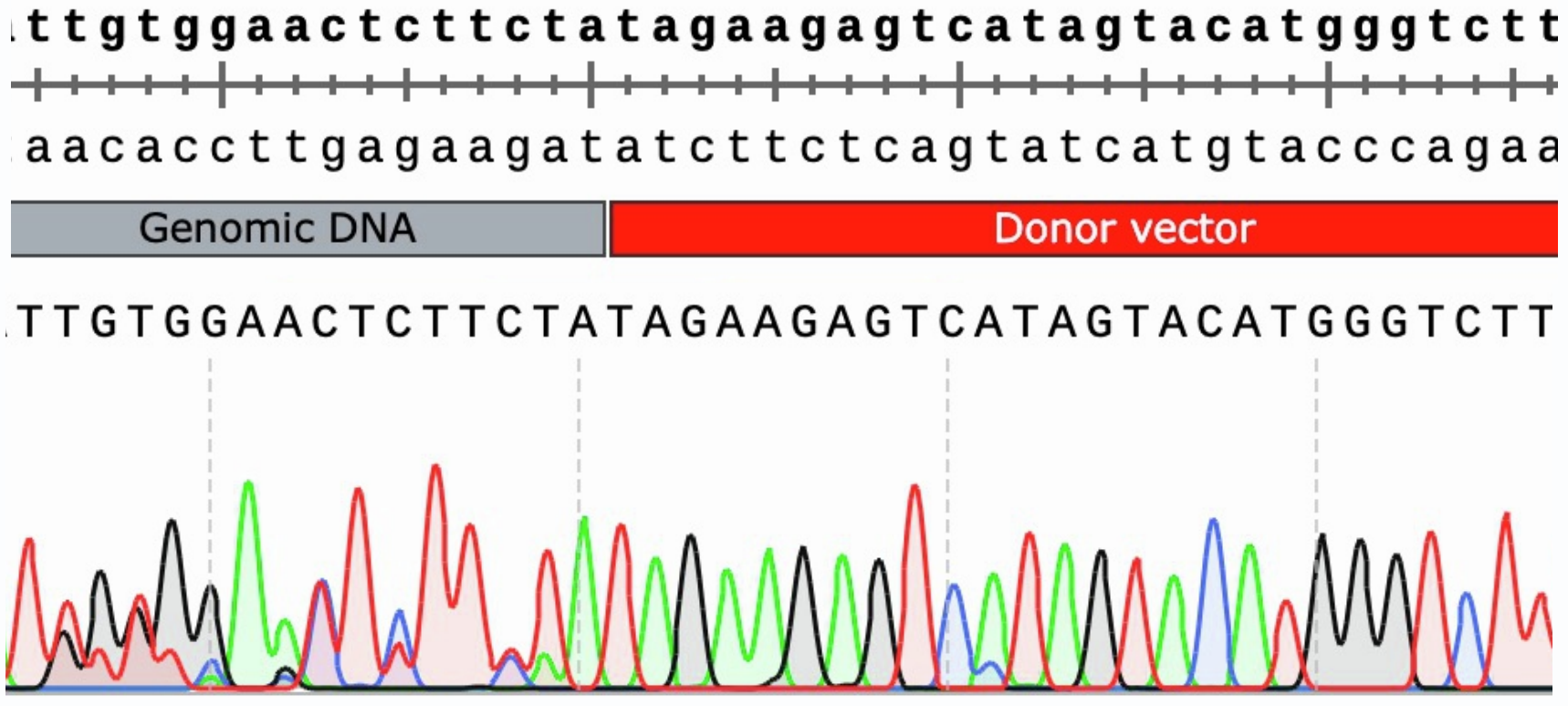

B

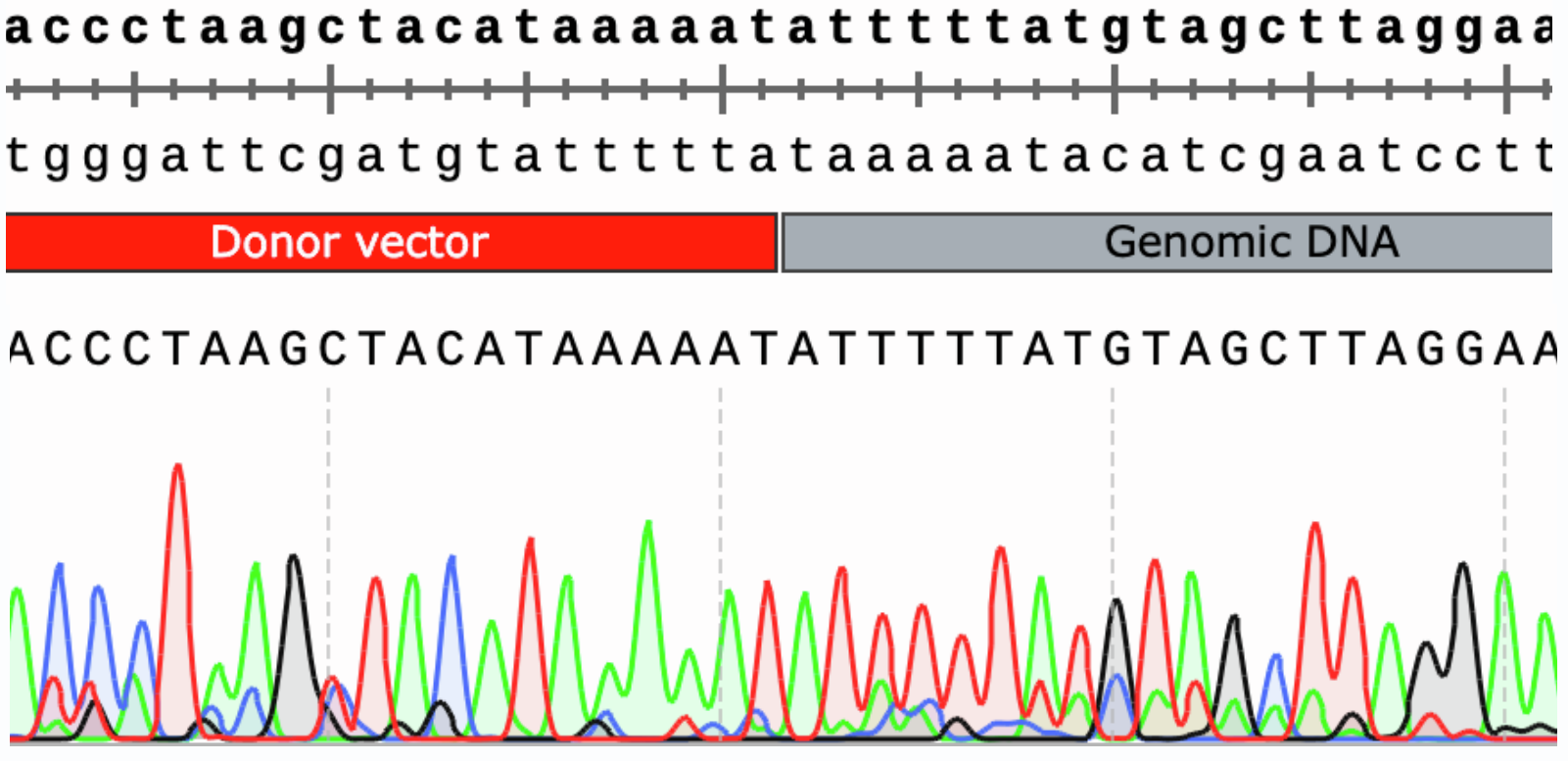

Figure S19.
Identification of predicted HITI junctions between the human genome and integrated OTC donor. (A) Upstream and (B) downstream junctions were confirmed by PCR using primers that span the predicted junction followed by Sanger sequencing of the amplicons.

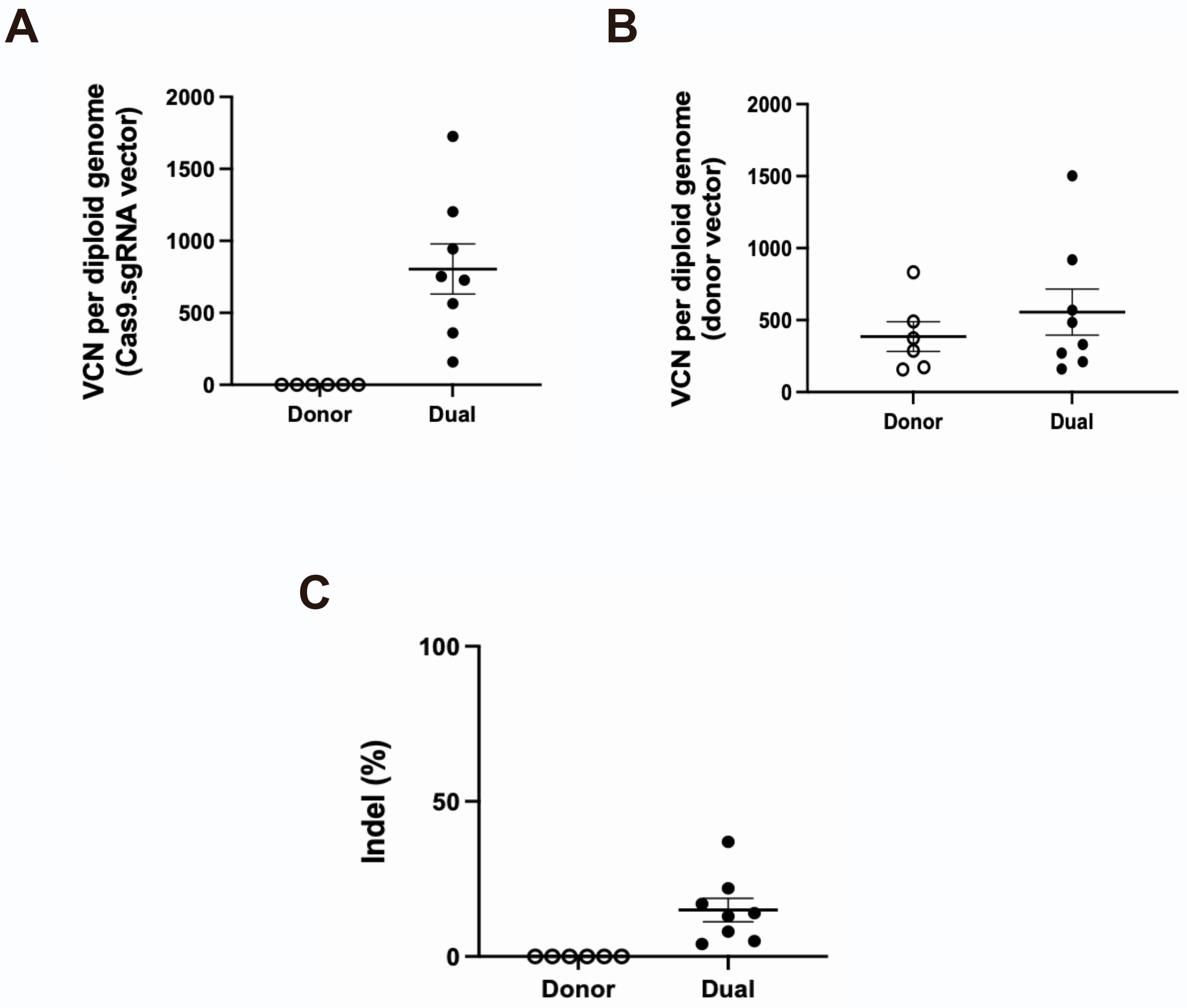

Figure S20.
Molecular analysis of FRG mouse livers engrafted with patient-derived primary human hepatocytes. Vector copy number of the (A) SaCas9 and (B) EGFP transgenes per diploid genome. (C) The percentage of indels identified in genomic DNA from chimeric mouse-human livers following delivery of dual AAV vectors (n=6 for the donor treatment group and n=8 for the dual treatment group, mean ± SEM are shown).

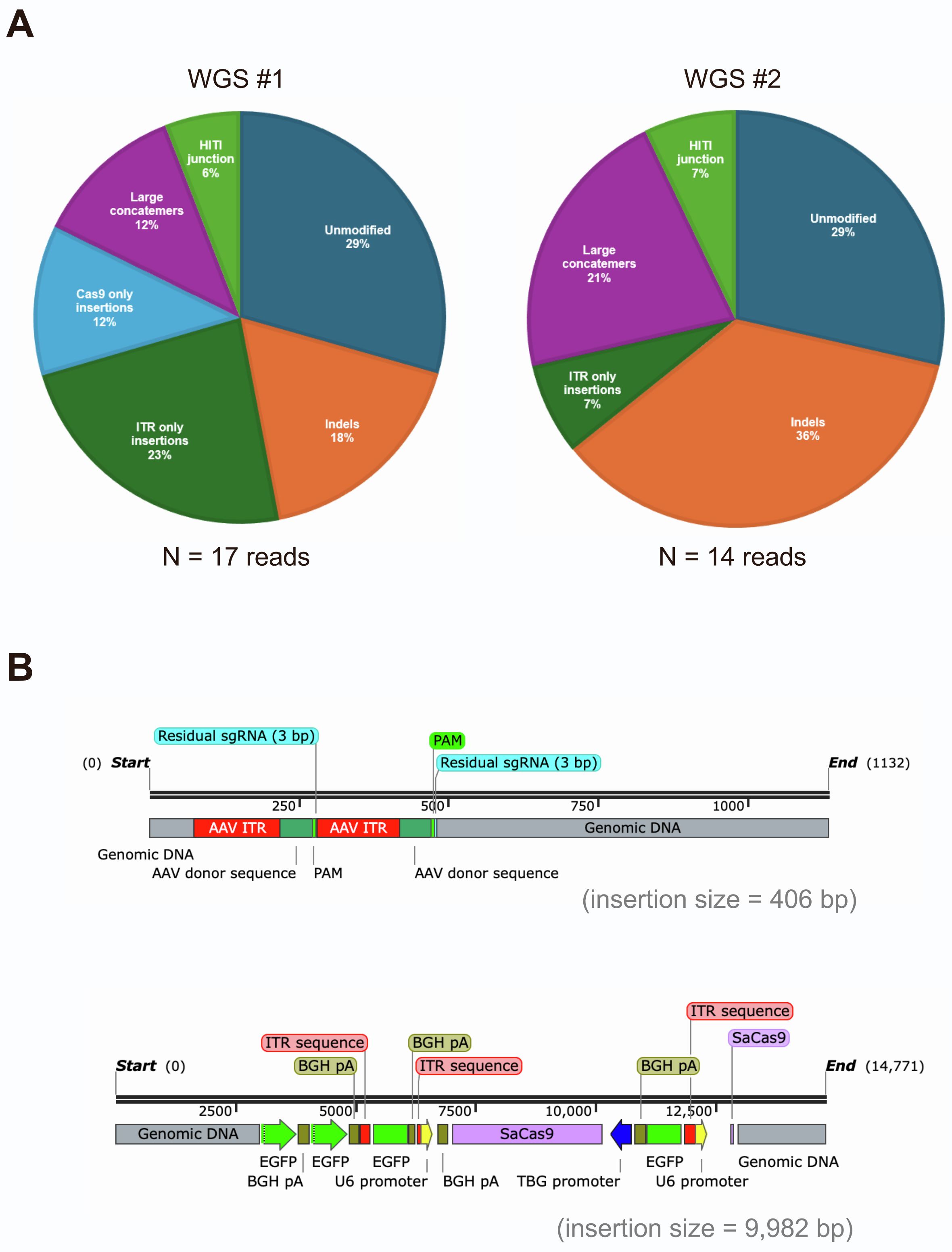

Figure S21.
Composition of integration events identified at the CRISPR-Cas9 cleavage site in murine intron 1. Genomic DNA was isolated from the liver of a C57Bl6 mouse treated with dual AAV vectors and subjected to long-read WGS to determine the types of integrations occurring at the CRISPR-Cas9 cleavage site. (A) Distribution of integration events and (B) Representative examples of integrations events observed at the target site.

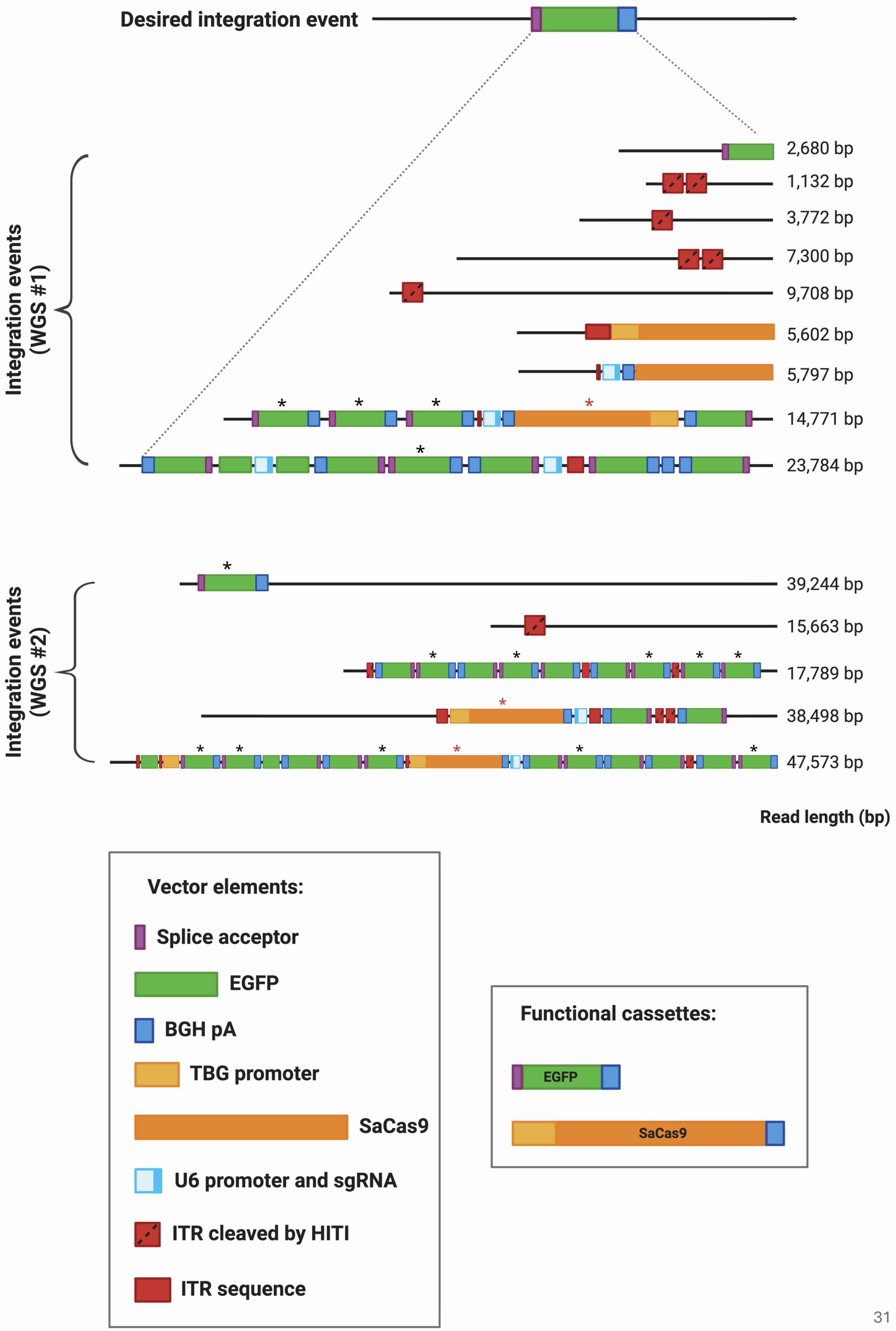

Desired integration event
Integration events (WGS #1)
2,680 bp
1,132 bp
3,772 bp
7,300 bp
9,708 bp
5,602 bp
5,797 bp
14,771 bp
23,784 bp
Integration events (WGS #2)
39,244 bp
15,663 bp
17,789 bp
38,498 bp
47,573 bp
Read length (bp)
Vector elements:
Splice acceptor
EGFP
BGH pA
TBG promoter
SaCas9
U6 promoter and sgRNA
ITR cleaved by HITI
ITR sequence
Functional cassettes:
EGFP
SaCas9

Figure S22.

Diagrammatic representation of the integration events identified at the target site in Otc intron 1. Genomic DNA was isolated from the liver of a C57Bl6 mouse treated with dual AAV vectors and subjected to long-read WGS. Total read lengths (bp) are indicated on the right. Integration events could be assigned to four main classes: the desired HITI event, insertion of ITR sequences cleaved from the donor vector, insertions containing the only the Cas9 vector or complex concatemeric sequences. For the two insertions containing only the Cas9 vector, the read did not contain the 3' junction so it was not possible to determine the full composition of the insertion or whether these events could lead to Cas9 expression. Donor cassettes that would be predicted to be functional, i.e. containing correctly orientated splice acceptor sequences (purple), full length EGFP transgene (green) and BGH pA (blue), are indicted by a black asterisk. Integrations of Cas9 vector that would be predicted to be functional are indicated by a red asterisk. Due to the complex nature of the concatemeric insertions, not all vector elements have been annotated. BGH pA, bovine growth hormone poly adenylation sequence; EGFP, enhanced green fluorescent protein, ITR, inverted terminal repeat; TBG, thyroxine-binding globulin promoter. Not to scale.
