# Supplementary material for: Functional editing of the OTC locus by targeted integration with phenotype correction and restoration of endogenous expression patterns
Source: Mol Ther. 2026 Jul 9;34(9):5426–43. doi: 10.1016/j.ymthe.2026.06.044 (PMC13555538; doi:10.1016/j.ymthe.2026.06.044)
Supplement: Supplementary file 2 [file mmc3.pdf]

Molecular Therapy
Original Article

Functional editing of the OTC locus by targeted integration with phenotype correction and restoration of endogenous expression patterns

Samantha L. Ginn,[1] Fatemeh Doroudian,[1] Sharntie Christina,[1] Otilia P.Y. Chan,[1] Caitlin W. Lucas,[1] Erhua Zhu,[1] Sile F. Yang,[1] Beena Devanapalli,[2] Anne H. Klein,[3] Suzanne Scott,[4] Joseph Vitale,[1] Sharon C. Cunningham,[1] Sophia H.Y. Liao,[1] Marti Cabanes-Creus,[4] Leszek Lisowski,[4,5] and Ian E. Alexander[1,6]

[1]Gene Therapy Research Unit, Children's Medical Research Institute, Faculty of Medicine and Health, The University of Sydney and Sydney Children's Hospitals Network, Westmead, NSW 2145, Australia; [2]NSW Biochemical Genetics Service, The Children's Hospital at Westmead, Westmead, NSW 2145, Australia; [3]Australian e-Health Research Centre, Commonwealth Scientific and Industrial Research Organisation (CSIRO), Herston, QLD 4006, Australia; [4]Translational Vectorology Research Unit, Children's Medical Research Institute, Faculty of Medicine and Health, The University of Sydney and Sydney Children's Hospitals Network, Westmead, NSW 2145, Australia; [5]Laboratory of Molecular Oncology and Innovative Therapies, Military Institute of Medicine, National Research Institute, 04-141 Warszawa, Poland; [6]Discipline of Child and Adolescent Health, Sydney Medical School, Faculty of Medicine and Health, The University of Sydney, Westmead, NSW 2145, Australia

Here, we report highly efficient functional repair of the ornithine transcarbamylase (OTC) locus in mutant mouse and human hepatocytes *in vivo* using a dual adeno-associated virus system delivering CRISPR-Cas9 editing reagents and a promoterless donor for targeted integration. The approach was mutation agnostic and targeted intronic sequences to prevent inadvertent inactivation of hypomorphic alleles. Notably, in a murine model, we corrected the metabolic defect and simultaneously achieved liver-wide restoration of physiological metabolic zonation of Otc expression by capturing native *cis*-acting regulatory elements. The effectiveness of this approach was confirmed using a universally configured therapeutic cassette in patient-derived primary human hepatocytes *in vivo*. These data provide a powerful template to guide further optimization of this approach and, given the high editing efficacy required for phenotypic effect in OTC deficiency, have broader relevance to other liver disease phenotypes.

INTRODUCTION

Previously, we employed a dual adeno-associated virus (AAV) vector-based approach using CRISPR-Cas9-mediated cleavage and homology-directed repair (HDR) for precise *in vivo* locus-specific single-nucleotide correction of a disease-causing mutation in patient-derived primary human hepatocytes.[1] Specifically, we focused on the urea cycle disorder ornithine transcarbamylase (OTC) deficiency, as a cell-autonomous model disease with a high therapeutic threshold. Clinical insights and mathematical modeling suggest that therapeutic efficacy in OTC deficiency correlates more strongly with the number of corrected hepatocytes than with the levels of OTC enzyme activity achieved in individual cells.[2,3] This renders OTC deficiency a relatively challenging therapeutic target, and accordingly, success in the treatment of this condition would have positive implications for a much broader set of liver diseases. In contrast, for non-cell-autonomous diseases that more commonly involve secreted proteins, gene transfer to only a modest number of hepatocytes has proven sufficient to confer clinical benefit.[4] Although we had achieved unprecedented rates of correction that if recapitulated in the clinic would provide unequivocal therapeutic benefit, in the current study, we have explored homology-independent targeted integration (HITI) as an alternative editing strategy. This approach, first described by Suzuki and colleagues,[5] is reported to exploit the non-homologous end joining (NHEJ) repair pathway for targeted integration of a therapeutic transgene in an orientation-specific manner. This is achieved by flanking the donor cassette with single-guide RNA (sgRNA) and protospacer-adjacent motif (PAM) sequences in the opposite orientation to the same target motif in the genome. Following co-delivery of the CRISPR-Cas9 editing machinery, both the target locus and donor cassette are cleaved, generating compatible DNA ends that can be attached through endogenous DNA end-joining pathways. While canonical NHEJ has frequently been implicated in this process, alternative end-joining mechanisms, including microhomology-mediated end joining (MMEJ), may also contribute depending on the genomic context, donor design, and repair environment.[6–8]

Our rationale for using an HITI approach was 3-fold. Firstly, classical HDR occurs preferentially in cycling cells, which will limit the potential number of editing events occurring in the liver.[9] Secondly, unlike base and prime editing systems that require bespoke reagents for individual mutations,[10] the therapeutic cassettes used in this study are

Received 28 January 2026; accepted 25 June 2026;
https://doi.org/10.1016/j.ymthe.2026.06.044.

Correspondence: Samantha L. Ginn, Gene Therapy Research Unit, Children's Medical Research Institute, Locked Bag 2023, Wentworthville, NSW 2145, Australia.
E-mail: sginn@cmri.org.au

Published by Elsevier Inc. on behalf of The American Society of Gene and Cell Therapy.
This is an open access article under the CC BY license (http://creativecommons.org/licenses/by/4.0/).

mutation agnostic, allowing all patients with OTC deficiency to be treated using the same validated set of reagents. Finally, and importantly, we directed the editing event to deep within the first intron of the OTC locus, away from intron-exon boundaries, thereby mitigating the risk of disrupting hypomorphic alleles that could negatively impact the patient and limit successful translation. For example, unwanted events such as large deletions occurring at the Cas9 cleavage site have the potential to disrupt the protein reading frame if the guide RNA and PAM sequences are located close to intron-exon boundaries.[11,12]

Here, we present results describing efficient targeted integration into the OTC locus following dual AAV-mediated delivery of CRISPR-Cas9 editing machinery and donor cassettes in both mouse and human hepatocytes *in vivo*. Importantly, we demonstrate efficient targeted integration where expression of the promoterless donor cassette is dependent on capturing the native Otc promoter resulting in liver-wide metabolic zonation, physiological patterns of gene expression, and correction of the metabolic phenotype in Otc-deficient animals. Moreover, the rates of integration achieved in patient-derived primary hepatocytes would provide therapeutic benefit if recapitulated in humans. While our results describe an editing strategy designed to treat OTC deficiency, they serve as a therapeutically indicative paradigm for liver-targeted gene therapy more broadly.

RESULTS

Targeted integration into the Otc locus confers liver-wide physiological patterns of gene expression

In this study, we employed an HITI approach using a dual AAV-based vector system to deliver both the editing machinery and donor template (Figure 1A). The HITI approach is configured to favor integration in the correct orientation because integrations in the reverse orientation are able to undergo further cleavage with the potential for subsequent integration events in the forward orientation to occur[5] (Figure 1A). One vector contains the *Staphylococcus aureus* Cas9 (SaCas9) under the control of a liver-specific thyroxine-binding globulin (TBG) promoter[14] and selected sgRNA (sgRNA1; Table S1). The second vector delivers the donor template for targeted integration into the nuclease-induced double-strand break at the endogenous Otc locus. The donor template was designed to insert a splicing-competent, promoterless cassette into the first intron of the murine Otc locus (Figure 1B). Both vectors were packaged in the AAV8 capsid, which is highly tropic for the murine liver.[15]

To evaluate the efficiency of the dual AAV editing system *in vivo*, adult male mice, containing a single Otc locus per genome, were harvested 4 weeks after vector delivery (Figure 1C). Upon targeted integration, expression of the EGFP transgene is driven from the native Otc promoter and would, therefore, be expected to display the same zonated expression pattern as Otc (Figure 1D). These integration events generate a hybrid transcript composed of the first exon of endogenous *Otc* in frame with the EGFP transgene (Figure 1E). In addition, small indels were identified using PCR primers spanning the SaCas9 cleavage site (Figure 1F), with the proportion of indels identified in genomes not containing an integration event reduced in the presence of the donor vector (Figure 2A). As expected, vector copy number correlated with the vector dose (Figure S1). Analysis of livers from treated mice showed high levels of EGFP expression, with up to 80.7% ± 2.6% of hepatocytes expressing EGFP at the most effective vector doses (Figures 2B and S2). Notably, the dose of the vector containing the editing machinery had the greatest influence on the percentage of EGFP-positive cells across the range of doses tested, most likely due to limiting availability of sgRNA as has been shown by others.[16–18] For example, there was no difference in the percentage of EGFP-positive cells observed at a donor vector dose of 1×10^{12}, 5×10^{11}, and 2.5×10^{11} vg/mouse if the dose of the vector containing the editing machinery remained constant (Figure 2B). Analysis of the composition of RNA transcripts revealed that up to 80.8% ± 0.6% were hybrid at the most effective vector doses (Figures 2C and S3; Table S2) and correlated with the levels of EGFP expression observed by flow cytometry. Notably, the proportion of hybrid transcripts exceeded that of the endogenous transcripts at all dual vector doses except for the lowest dose of 2.5×10^{11} vg of each vector/mouse. In contrast, however, the number of precise HITI junctions in total liver genomic DNA, while present, was lower (Figure S4). This suggests that, although the resultant integration events have functional outcomes, a significant proportion of these events are configured imperfectly. These results were supported by the observation of liver-wide EGFP expression in sections from mice treated with dual vectors at all doses (Figures 2D and S5A). Importantly, EGFP expression followed physiological patterns, showing reticular periportal distribution consistent with known OTC metabolic zonation.[19] This was not observed in single vector-treated control animals (Figure S5B).

Phenotype correction in Otc-deficient mice following targeted integration

To assess therapeutic potential of our dual AAV editing approach, we next treated adult male Otc-deficient *Spf*^{ash} mice (Figure 3A) with 1×10^{12} vg/mouse of the vector containing the editing reagents and 5×10^{11} vg/mouse of the donor and monitored urinary orotic acid levels as a measure of urea cycle function and the most sensitive indicator of phenotype correction.[20] Notably, the *Spf*^{ash} mouse displays a mild phenotype due to residual Otc activity sufficient to control ammonia levels but not urinary orotic acid. This phenotype results from a point mutation at the final nucleotide of exon 4 that disrupts splice donor recognition and reduces pre-mRNA splicing efficiency.[21] Using an editing strategy mirroring that used for the EGFP transgene, the therapeutic donor cassette contained a codon-optimized human *OTC* complementary DNA (cDNA) encoding exons 2 to 10, which was configured to splice with the endogenous murine *Otc* exon 1 following targeted integration (Figure 3B). If applied in the clinical setting, this construct would not be therapeutic for patients with mutations located in either exon 1 or those that affect upstream regulatory elements. Of note, this produces a hybrid mouse-human mRNA transcript and resultant translated protein, which is a fusion between the first exon of murine *Otc*

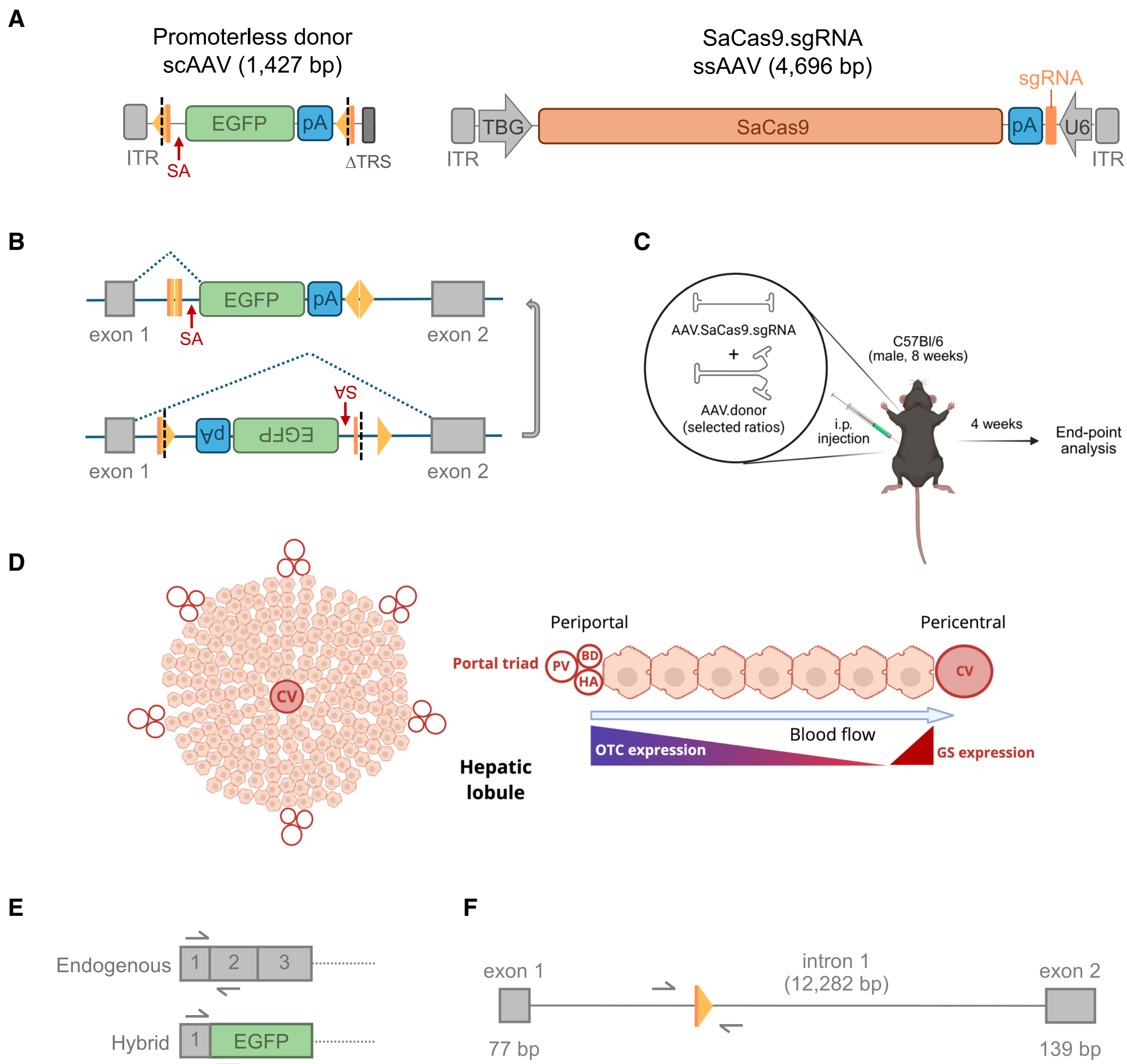

Figure 1. Experimental overview

(A) Schematic representation of the dual AAV vectors used for homology-independent targeted integration (HITI) into the first intron of the Otc locus. One vector contains a promoterless donor DNA in the self-complementary form and the second, the Cas9 nuclease from *Staphylococcus aureus* (SaCas9) under the transcriptional control of a liver-specific TBG promoter and a single-guide RNA (sgRNA) driven from a U6 promoter. Both vectors were packaged in the AAV8 capsid. The locations of sgRNA (orange rectangle) and PAM (yellow triangle) sequences in the first intron of the murine Otc locus are indicated. (B) Outcomes of targeted integration in the correct and reverse orientation into the first intron of the Otc locus. Following the desired integration event, a single transcript is produced containing the first exon of murine *Otc* and the EGFP reporter. Blue dotted lines indicate the location of pre-mRNA splicing to generate the mature transcript. (C) Schematic representation of the dual AAV delivery system in adult C57BL/6J mice. (D) Diagrammatic representation of the hexagonal-shaped hepatic lobule with a central vein (CV) in the middle and portal triad corners (left panel), with the acinus extending from a portal triad into adjacent central veins (right panel). Expression of Otc exhibits metabolic zonation, where it is expressed most highly in the periportal regions, with negligible expression in pericentral zones. Glutamine synthetase (GS) expression is restricted to pericentral hepatocytes surrounding central veins. For these enzymes, this zonated expression pattern is mutually exclusive and provides complementary metabolic functions for ammonia detoxification.[13] BD, bile duct; CV, central vein; HA, hepatic artery; PV, portal vein. (E and F) Expected mRNA transcripts following targeted integration and (F) Location of primer binding sites and sgRNA (rectangle) and PAM (triangle) sequences in the first intron of the murine Otc locus. ITR, inverted terminal repeats; SA, splice acceptor signal; EGFP, enhanced green fluorescent protein; pA, bovine growth hormone polyadenylation signal; ΔTRS, deletion in the terminal resolution site; TBG, human thyroxine binding globulin promoter; U6, RNA polymerase III promoter for human U6 snRNA; SaCas9, Cas9 from *Staphylococcus aureus* with hashed vertical lines used to indicate the location of the SaCas9 cleavage sites. Yellow arrow heads indicate the location of the sgRNA sequence and orange rectangles the protospacer-adjacent motif (PAM).

and the integrated human *OTC* exons 2–10. Following treatment, the urinary orotic acid levels in mice receiving both AAV vectors normalized by 3 weeks post treatment (Figure 3C). At the end of the study, the values for the dual-treated mice, although still mildly elevated, were not significantly different from wild-type animals and were significantly lower than untreated Spf^{ash} controls (p = 0.0068,

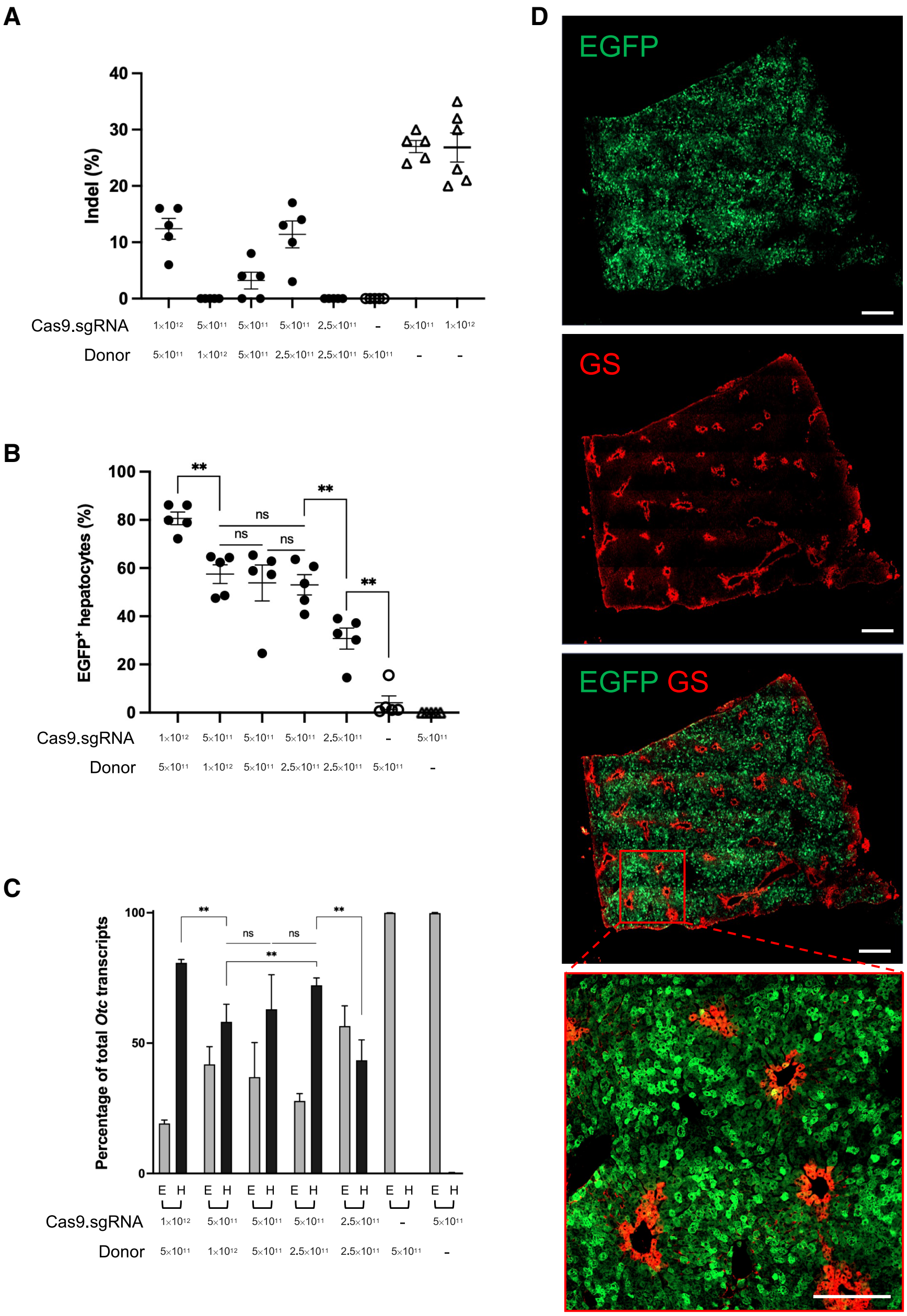

Figure 2. Physiological expression patterns following targeted integration

(A) Quantification of indel frequency following targeted integration. Genomic DNA was isolated from whole liver 4 weeks after dual-vector delivery and the percentage of indels determined by PCR using primers that span the predicted SaCas9 cleavage site followed by Sanger sequencing of the amplicons. (B) Fluorescent activated cell sorting (FACS) analysis of perfused liver samples following dual AAV delivery. The percentages of EGFP-positive cells over a range of vector doses are indicated. (C) Quantification of transcripts from the *Otc* promoter with the proportion of endogenous transcripts "E" and hybrid transcripts "H" shown. (D) Reporter gene expression following integration of the donor template in murine hepatocytes shows metabolic zonation. Mosaic images from an animal receiving 1×10^{12} vg/mouse of the AAV vector containing the editing reagents and 1×10^{11} vg/mouse of the AAV donor. Sections were labeled with antibodies against the EGFP (green) and glutamine synthetase (GS, red) proteins. Higher magnification region of interest is shown in the bottom panel. Scale bars represent 500 μm. Animals receiving either the donor or editing reagents alone were included as controls. Doses of the SaCas9.sgRNA and EGFP-containing donor vectors are shown below the graph. Data are plotted as mean ± SEM and significance evaluated using a one-way ANOVA followed by a Tukey's multiple comparisons test (*** <0.001, ** <0.01, * <0.05, and ns = not significant). *n* = 5 per treatment group.

Tukey's one-way ANOVA). To further assess therapeutic benefit conferred by our editing strategy, additional mice underwent a high-protein diet challenge 8 weeks after vector delivery. Plasma ammonia levels were found to be highly elevated in untreated *Spf*^{ash} controls and the donor-only treatment group. In contrast, animals from the dual-vector treatment group were able to control plasma ammonia levels after the high-protein challenge and had only mildly elevated levels when compared with the wild-type controls (Figure 3D). Untreated wild-type animals and all mice receiving both AAV vectors survived the high-protein challenge, whereas the untreated *Spf*^{ash} mice and four of the five animals (80%) receiving only the donor vector developed clinical signs of hyperammonemia and required euthanasia on the second day of the 3-day course.

Using an *in situ* assay on whole liver sections, Otc activity was detected in up to 40% of hepatocytes (33.2% ± 2.6%, *n* = 7 mice), again displaying physiological patterns of metabolic zonation (Figure 4A), although the activity observed in individual hepatocytes was considerably lower than wild-type levels (Figure 4B). Targeted integration at the Otc locus was further confirmed using direct immunohistochemical staining of liver sections (Figure 4C), again with clear restoration of Otc expression showing metabolic zonation observed in up to 31% of hepatocytes (24.1% ± 2.2%, *n* = 7). This is lower than the number of transgene-positive cells observed in wild-type mice with the EGFP donor at the same vector doses (Figure 2B), which likely reflect different sensitivities between fluorescent activated cell sorting (FACS) and immunofluorescence when used for detection. Such differences were also observed in wild-type mice (Figure 4B compared with Figure 4D); however, metabolic zonation was clearly visible for both *in situ* enzyme activity and antibody labeling (Figure 4A compared with Figure 4C).

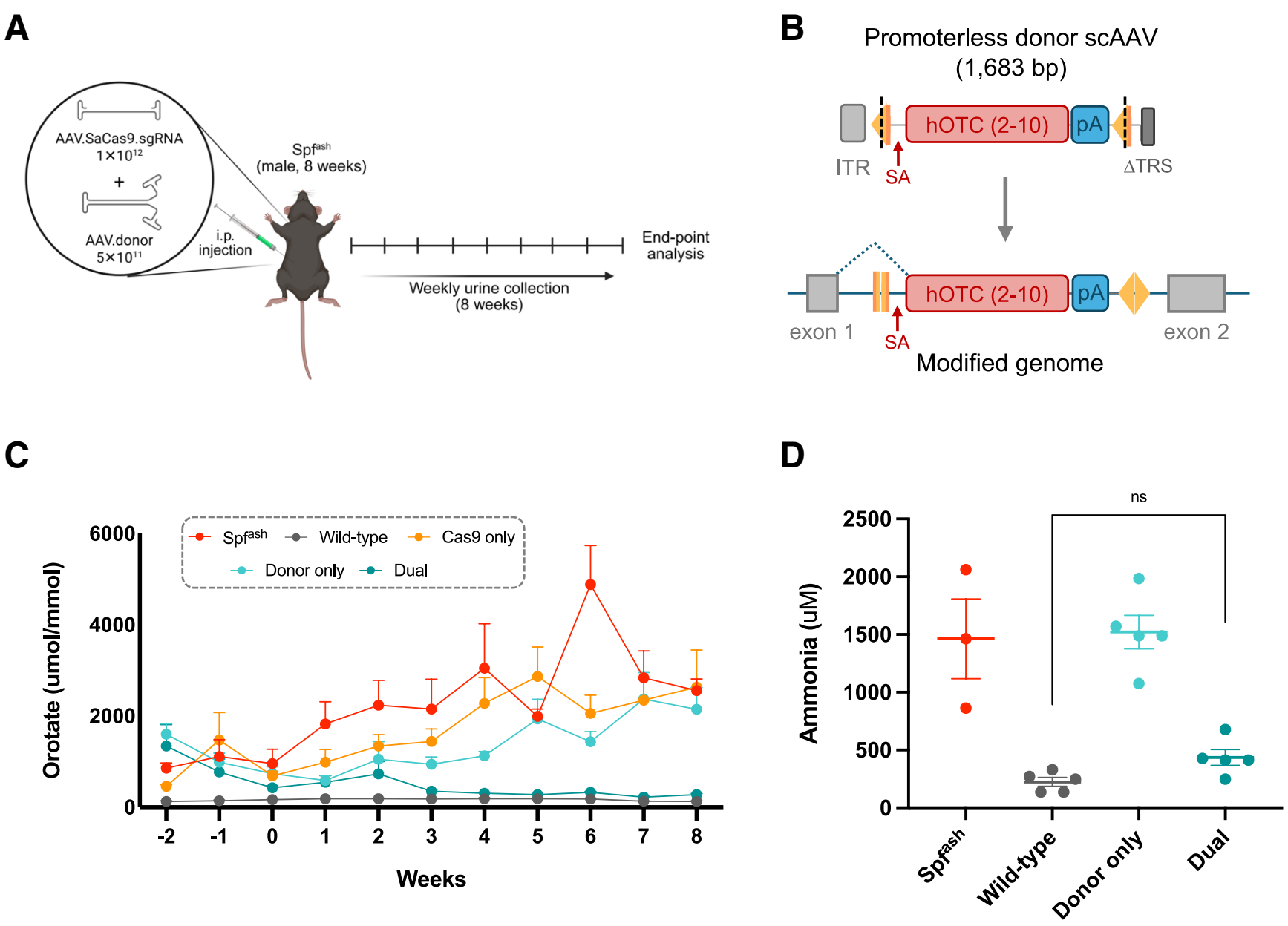

Figure 3. Phenotype correction in Otc-deficient *Spf^ash* mice following targeted integration

(A and B) Diagrammatic representation of (A) the experimental design for dual AAV delivery to adult *Spf^ash* mice and (B) the self-complementary AAV vector containing the donor for phenotypic correction. Animals received 1×10^{12} vg/mouse of the AAV vector containing the editing reagents and 5×10^{11} vg/mouse of the AAV donor as indicated. Both vectors were packaged in the AAV8 capsid. The configuration of the vector is the same as the EGFP-containing donor, with the EGFP reporter replaced by the coding sequence of human *OTC* exons 2 to 10. Following the desired integration event, a single transcript is produced containing the first exon of murine *Otc* and exons 2–10 of human *OTC*. (C) Urinary orotate levels, normalized to creatinine, over an 8-week period following treatment. Urine was collected weekly, with two collections prior to dual AAV vector delivery. $n = 6$ per treatment group for all groups except for the dual-treated cohort, where $n = 7$. (D) Plasma ammonia (μM) levels in mice following a high-protein diet challenge. Data are plotted as mean ± SEM and significance evaluated using a one-way ANOVA followed by a Tukey's multiple comparisons test; ns = not significant. $n = 3$–5 per treatment group.

Analysis of *Otc* transcripts in the liver of animals receiving both vectors revealed that 58.1% ± 3.1% were hybrid (Figures 4E and S6A). The composition of precise HITI junctions in genomic DNA, vector copy number, and the percentage of indels and from whole liver agreed with the results observed in C57BL/6J mice following delivery of the EGFP-containing donor vector (Figures S6B and S7–S9), again revealing that a lower proportion of correct up- and downstream junctions relative to the desired transcript are present following targeted integration. This observation underscores an important advantage of our deep-intronic approach where functional outcomes can result from imprecise editing events due to the inclusion of RNA splicing sequences in the donor vector.[5] In addition to the appearance of hybrid transcripts following delivery of the therapeutic vector system, there was a higher total number of transcripts when compared with other *Spf^ash* treatment groups (Figure 4E). Although not statistically significant, this observation is notable because the targeted integration events occur in the first intron of the *Otc* gene, which lies upstream of the splice donor site mutation in the *Spf^ash* mouse that causes inefficient pre-mRNA splicing. Furthermore, even though Otc activity in whole liver lysates was not restored to wild-type levels, they were greater than 2-fold higher than the level found in untreated *Spf^ash* mice (Figure 4F) with corrected cells distributed across the liver (Figures 4A–4C). Importantly, although this increase did not reach statistical significance (Table S3), it was sufficient to normalize urinary orotic acid levels (Figure 3C) and to control plasma ammonia levels following a high-protein challenge (Figure 3D), which is consistent with the cell-autonomous nature of OTC where the both the distribution and absolute number of hepatocytes expressing OTC is more therapeutically relevant than measures of liver-wide enzymatic activity.

Efficient targeted integration into primary human hepatocytes *in vivo*

An important next step for translation is the evaluation of human-specific reagents in clinically predictive model systems. To this end, we examined our approach *in vivo* in primary human hepatocytes from male donors engrafted into the $Fah^{-/-}Rag2^{-/-}Il2rg^{-/-}$ (FRG) mouse liver. Given the experimentally limiting supply of patient-derived primary human hepatocytes, we first sought to functionally evaluate targeted integration efficiency using locus-specific editing reagents in wild-type hepatocytes (Figure 5A). We again employed a dual AAV delivery system, with one vector encoding SaCas9 and a sgRNA (sgRNA1) that targets the first intron of the human OTC locus with high efficiency and specificity (Figures 5B and 5C; Table S4). Although the analysis was limited, no off-target cleavage was detected at the top *in silico* predicted sites in the human genome (Figure S10). The second vector contained a donor encoding an EGFP reporter (Figure 5D). Animals received 5×10^{11} vg/mouse of the vector containing the editing reagents and 1×10^{11} vg/mouse of the AAV donor. We further configured our targeting integration approach to be mutation agnostic by including two constructs that used either an internal ribosome entry site (IRES) from encephalomyocarditis virus (EMCV) or the eukaryotic initiation factor 4G (EIF4G) sequence, both of which have been shown to have high translational activity in the liver.[22,23] The inclusion of an IRES element allows expression of a second downstream gene (either EGFP or the entire human *OTC* cDNA used later in this study) following targeted integration, making this approach universal for all OTC coding mutations. All vectors were packaged in the NP59 capsid, which has been shown to have high tropism for the human liver and was used in our previous study examining HDR-mediated

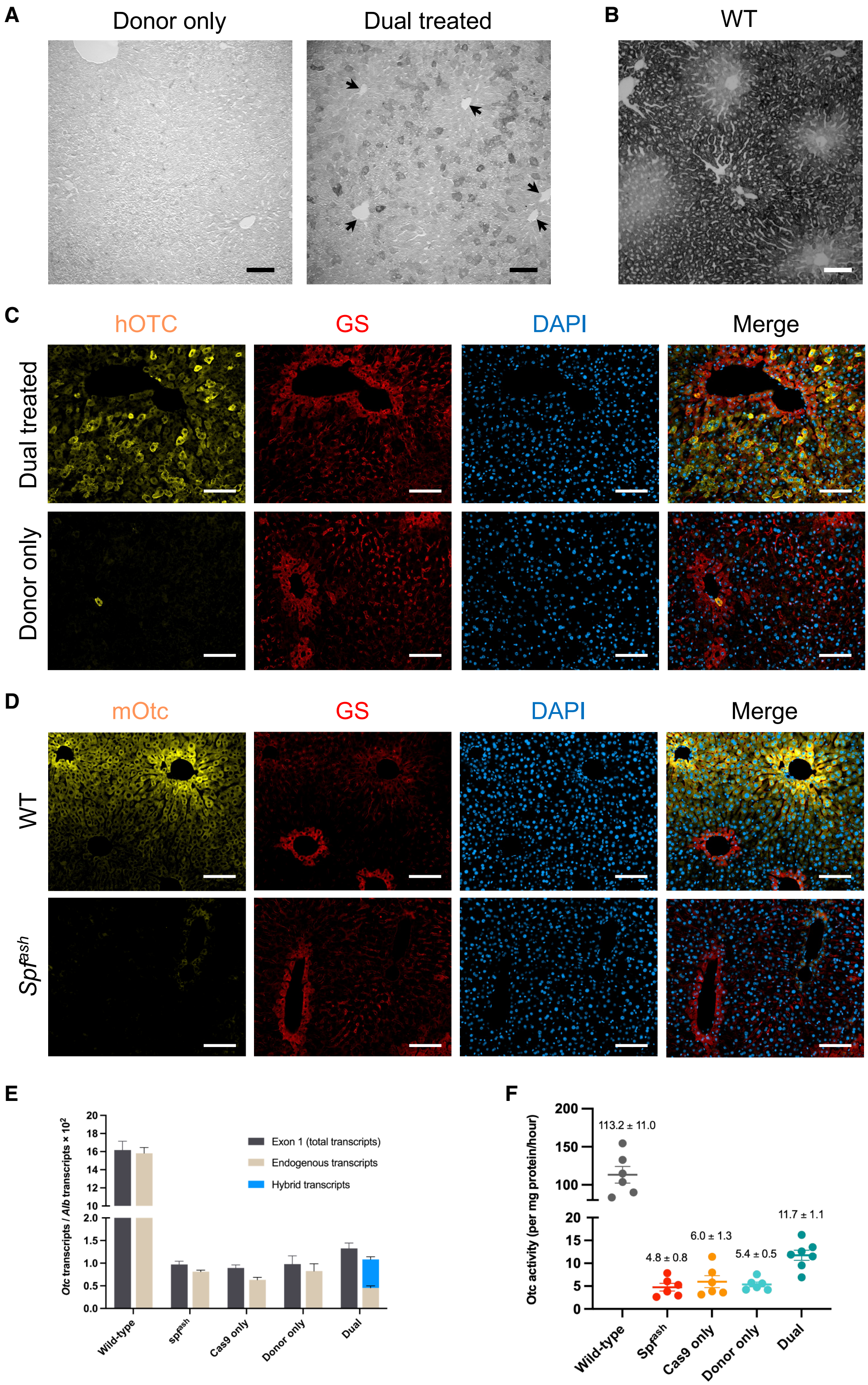

(legend on next page)

correction of patient-derived primary human hepatocytes *in vivo*.[1,24] Vector was administrated while mice were on a water cycle where the human hepatocytes were undergoing selective expansion and the circulating human albumin levels were between 0.2 and 2.0 mg/mL (Table S5). Consistent with the tropism of NP59 for human hepatocytes, molecular analysis confirmed the presence of AAV vector genomes and indels with all vector combinations at a high level (Figures 5E and S11).

Analysis of livers from engrafted FRG mice demonstrated that, although variable, up to 91.8% of primary human hepatocytes expressed EGFP, and no significant difference in the number of transgene positive cells was observed between the donor configurations (Figure 5F). Of note, the same pattern of metabolic zonation seen in the murine liver was not observed in primary human hepatocytes in the context of chimeric livers in FRG mice. This is not unexpected because reports have suggested that although zonation-associated gene expression can be partially re-established after engraftment, current evidence indicates this represents incomplete or altered zonation rather than faithful recapitulation of the human state.[25,26] In addition, although no difference in the number of EGFP-positive hepatocytes was observed, the intensity of expression differed. Unsurprisingly, the level of transgene expression was highest in the absence of an IRES element, consistent with previous reports using these elements. Furthermore, the EIF4G sequence was found to be superior to the EMCV IRES as predicted (Figure S12).[23] Notably, liver-wide transgene expression was observed in the human hepatocytes (Figure 6A) and, following treatment with the dual-vector system, was associated with the expected loss of OTC expression (Figure 6B). This observation is consistent with expression being driven by the endogenous OTC promoter, the consequence of targeted integration events. In contrast, in livers from engrafted mice receiving only the donor vector, the number of cells expressing EGFP was both significantly reduced and not associated with loss of the OTC protein (Figure 6B, bottom panels). This is consistent with untargeted random AAV integration capturing other cellular promoters for transgene expression. Precise junctions between the human genome and integrated donor fragment were identified, again, lower than the number of EGFP-positive human hepatocytes with approximately 2-fold more integrations detected in the correct orientation (Figures S13 and S14).

Targeted integration restores OTC protein expression in patient-derived primary human hepatocytes *in vivo*

Prior to testing the validated editing reagents in OTC-deficient patient-derived primary human hepatocytes, a series of AAV capsids with high tropism for the human liver were compared with NP59 for *in vivo* editing efficiency (Figure S15). We identified SYD12 as the best performing capsid.[27] We further confirmed that the dose of 5×10^{11} vg/mouse was optimal using the percentage of indels as a surrogate readout for editing efficiency (Figure S16). We next tested the ability to perform targeted integration using the dual AAV editing system in OTC-deficient patient-derived primary human hepatocytes, obtained from the liver removed from an infant at the time of liver transplantation.

Based on the results obtained using the donor vectors encoding the EGFP reporter, we chose to evaluate a universal donor construct encoding a codon-optimized full-length human *OTC* cDNA behind the EIF4G sequence in patient-derived OTC-deficient primary human hepatocytes *in vivo*. Evidence of targeted integration into the human OTC locus was observed by both direct immunohistochemical staining of chimeric liver sections (Figure 7A) and FACS analysis, where clear restoration of OTC expression was observed in up to 48% of human hepatocytes (Figures S17 and S18). Targeted integration was confirmed in dual-treated animals by interrogating samples obtained from whole liver. Treated animals contained both a high proportion of vector-encoded OTC transcripts (Figure 7B) and precise HITI junctions (Figures 7C and S19). The presence of both vectors was confirmed by using digital PCR (dPCR) (Figures S20A and S20B). Interestingly, like results obtained in wild-type and Otc-deficient mice, the proportion of indels identified in genomes not containing an integration event was reduced in the presence of the donor vector (compare Figure 7C with Figure S20C). Collectively, these data confirm targeted integration into the OTC locus in both wild-type and patient-derived primary human hepatocytes *in vivo*.

Integration events at the target locus are heterogeneous in nature

Given the disparity observed between the number of precise HITI junctions amplified from the liver of treated animals and the functional outcomes observed in both murine and human hepatocytes, we sought to better understand the architecture of integration events

Figure 4. Detection of hybrid *Otc* transcripts and restoration of Otc protein expression and enzymatic activity in Otc-deficient *Spf^ash* mice after targeted integration

(A–D) *In situ* Otc enzyme activity in representative images of liver sections from (A) *Spf^ash* mice treated with either the donor or dual AAV vectors and (B) an untreated wild-type animal. The location of central veins in the dual-treated image are indicated with back arrow heads. Scale bars represent 50 μm. Restoration of Otc expression was observed following targeted integration. Sections were labeled with antibodies against (C) human OTC or (D) mouse Otc (yellow) and glutamine synthetase (GS, red) proteins. Nuclei were counter-stained with DAPI (blue). Scale bars represent 100 μm. (E) Quantification of transcripts from the *Otc* promoter. The total number of transcripts was determined using primers with binding sites located wholly within exon 1 (dark gray bars). The proportion of endogenous and hybrid transcripts was determined by digital PCR (dPCR) using a common forward primer in combination with a reverse primer specific to either the endogenous murine *Otc* or vector-encoded *OTC* transcripts, where the primer binding sites span the exon 1-exon 2 boundary. Transcripts were normalized to murine albumin. (F) Otc enzyme activity from individual animals in whole-liver lysates. Data are plotted as mean ± SEM, and $n = 6$ per treatment group for all groups except for the dual-treated cohort, where $n = 7$. Significance was evaluated using a one-way ANOVA followed by a Tukey's multiple comparisons test. All animals received 1×10^{12} vg/mouse of the AAV vector containing the editing reagents and 5×10^{11} vg/mouse of the AAV donor.

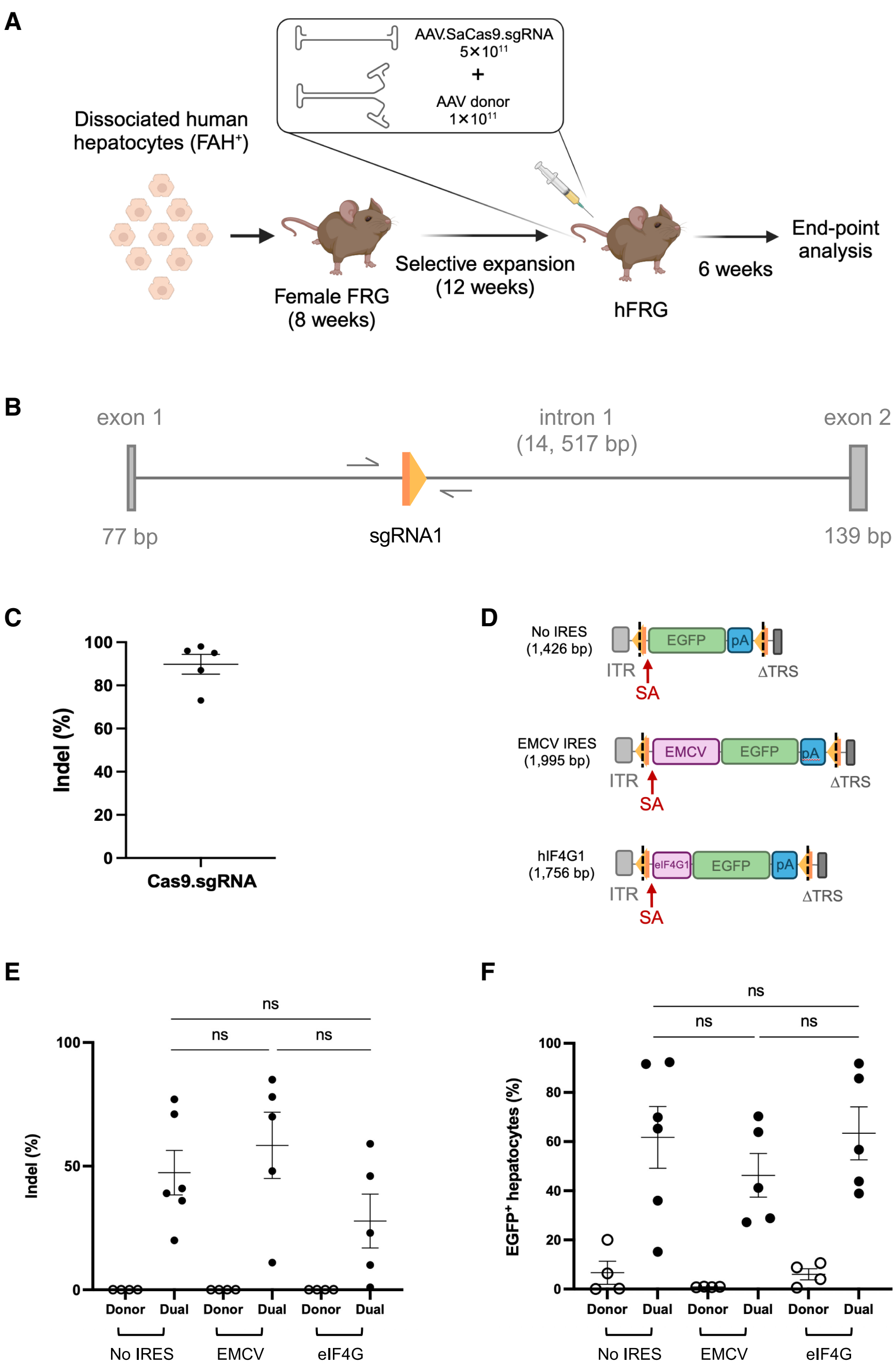

Figure 5. Efficient targeted integration into primary human hepatocytes *in vivo*

(A) Wild-type primary human hepatocytes were engrafted into FRG mice and repopulation established by cycling on and off the drug 2-(2-nitro-4-fluoromethylbenzoyl)-1,3-cyclohexanedione (NTBC). 12 weeks later, animals received 5 × 10^{11} vg/mouse SaCas9-sgRNA vector and 1 × 10^{11} vg/mouse of the donor vectors packaged in the NP59 capsid via the tail vein. Livers were analyzed 6 weeks following vector delivery. (B–C) Determination of indel frequency across the SaCas9 cleavage site (sgRNA1) in the first intron of the human OTC locus. (B) Location of primer binding sites and the sgRNA (rectangle) and PAM (triangle) sequences in intron 1 are shown and (C) the percentage of indels in genomic DNA from chimeric mouse-human livers following delivery of the SaCas9-sgRNA vector only (n = 5). (D) Schematic representation of the EGFP-containing donor vectors used in this study; the first construct was analogous to the donor evaluated in C57BL/6J mice, with the other two containing IRES elements. EMCV, IRES element from encephalomyocarditis virus; eIF4G, eukaryotic initiation factor 4G element. (E) The percentage of indels in genomic DNA from chimeric mouse-human livers after dual AAV vectors were determined (n = 4–6 animals per treatment group). (F) FACS analysis of perfused chimeric mouse-human livers following dual AAV delivery. The percentage of EGFP-positive cells in wild-type primary human hepatocytes are indicated for each of the three donor vector construct's significance. Data are plotted as mean ± SEM and significance evaluated using a one-way ANOVA followed by a Tukey's multiple comparisons test; ns = not significant.

occurring at the CRISPR-Cas9 cleavage site. To achieve this, we subjected genomic DNA extracted from the liver of one C57BL/6J mouse displaying high levels of EGFP expression (78.9% by FACS) to long-read whole-genome sequencing (WGS) to obtain a sample of unbiased integration events. Interrogation of the reads spanning the CRISPR-Cas9 cleavage site in the first intron of *Otc* revealed the presence of unmodified genomes, expected small indels resulting from imperfect repair, and a heterogeneous pattern of integration events (Figures S21 and S22; Table S6). Although only a modest number of targeted integration events were captured, notably, the desired HITI-mediated integration event was in the minority, with an integration event containing the ITR sequences that were released upon CRISPR-Cas9-mediated cleavage of the donor vector more common. Long-read sequencing also identified integration events containing the Cas9 editing machinery and long AAV concatemers, the latter, a finding that has been observed by others.[28,29] In three of the five cases, the long concatemeric sequences contained a Cas9 expression cassette that would be predicted to be functional (Figure S22), highlighting the need to consider alternative delivery systems for transient nuclease expression.

However, more frequently the integration events consisted of concatemerized EGFP donor cassettes, where in four of the five cases, at least one was full length and correctly oriented. This configuration would result in expression of the transgene from the native *Otc*

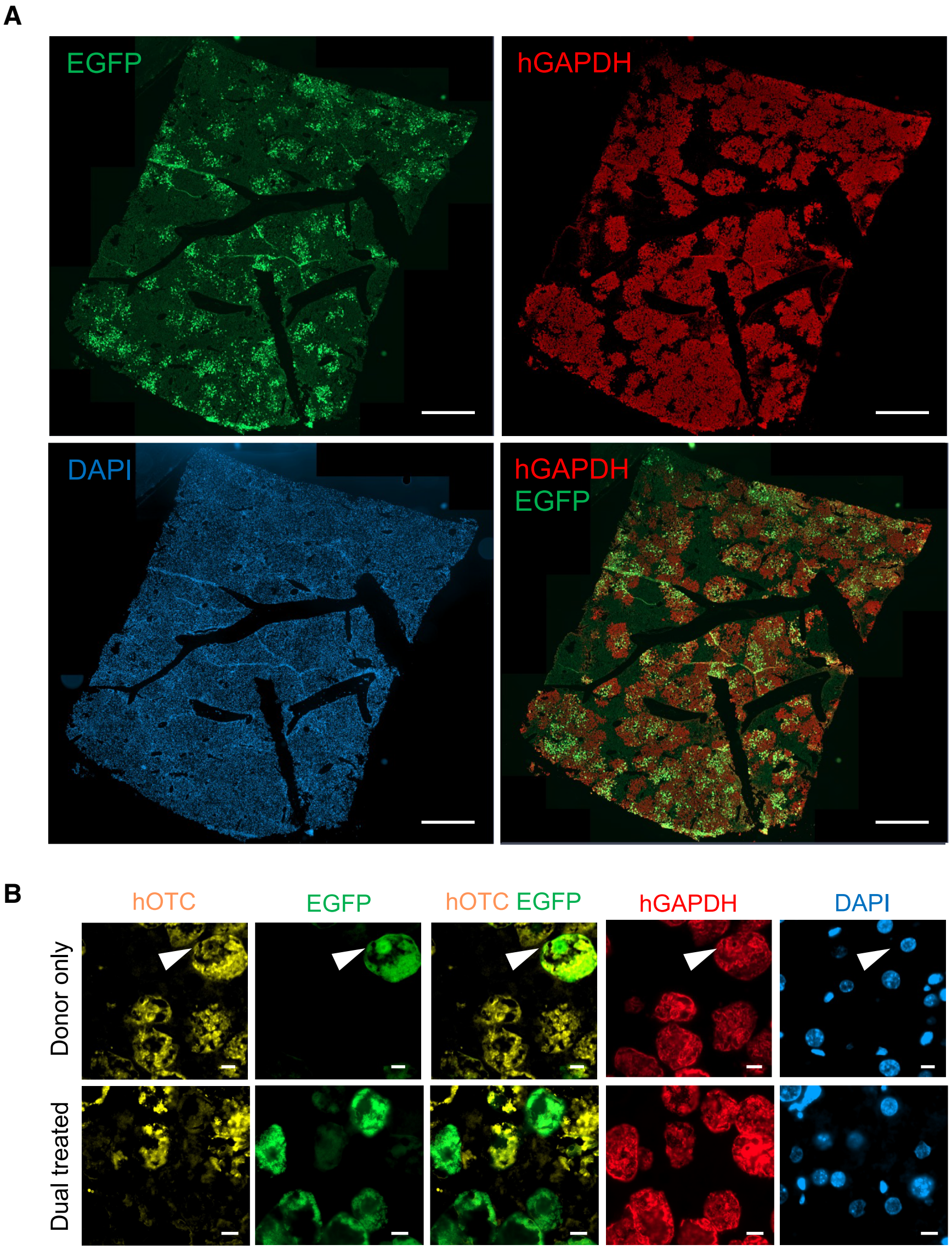

Figure 6. Reporter gene expression following targeted integration in primary human hepatocytes *in vivo*

(A) Mosaic images of chimeric liver sections from an animal receiving 5×10^{11} vg/mouse of the AAV vector containing the editing reagents and 1×10^{11} vg/mouse of the AAV donor. Sections were labeled with antibodies against the EGFP (green) and human GAPDH (red) proteins, and nuclei were counter-stained with DAPI (blue). Scale bars represent 500 μm. (B) High-magnification images of liver sections in dual- and donor-only-treated animals with sections stained as indicated above. White arrowheads indicate a primary human hepatocyte (red) that is both OTC and EGFP positive in an animal that received the donor vector only. Scale bars represent 10 μm.

promoter upon integration. This observation suggests that these concatemeric forms, although unintended, make a significant contribution to the functional editing events after splicing of the RNA transcript. To confirm these observations, an additional sample was subjected to WGS sequencing with adaptive sampling (targeting the Otc locus at the Cas9 cleavage site), which identified a similar pattern of heterogeneous integration events in 14 reads, consisting of long AAV concatemers (21%), ITR insertions (7%), and the desired HITI event (7%) (Figure S21).

DISCUSSION

The development of genome editing approaches capable of safe and efficient functional restoration of mutant genes in the human liver remains a challenge. While mutation-specific editing technologies that return the target locus to its native state are conceptually appealing,[30,31] there are formidable practical and logistic constraints to such approaches given the high number of different mutations underlying most genetic/metabolic liver disease phenotypes.[32,33] Mutation-agnostic approaches address these constraints but invariably demand the installation of large sequence elements, not currently possible using RNA-based approaches.[30,34] Using dual-AAV-mediated gene delivery, we have configured an HITI editing strategy that simultaneously addresses these challenges and evaluated efficacy in the context of OTC deficiency, a therapeutically challenging exemplar metabolic liver disease.[2,35] We report targeted integration with functional repair of the mutant Otc locus in adult *Spf*^{ash} mouse and human patient-derived hepatocytes *in vivo* at efficiencies of up to 40% and 47%, respectively. Even higher efficiencies of up to 81% and 92% were achieved in wild-type mouse and human hepatocytes as measured by capture of the endogenous OTC promoter and expression of a donor EGFP reporter cassette. Importantly, the levels of functional locus repair achieved at the mutant Otc locus in *Spf*^{ash} mice were sufficient to correct the metabolic phenotype and reconstitute physiological metabolic zonation of Otc expression across the hepatic lobule[13] by capture of the native endogenous *Otc* enhancer-promoter. This capture allowed elimination of exogenous enhancer-promoter elements from the therapeutic rescue cassette, which was targeted to deep within the first intron of the OTC locus and configured to universally correct all coding region mutations in an agnostic manner.

The high rates of functional edits achieved at the target loci in this study exceed published hepatocyte editing efficiencies.[36–40] Using a dual AAV system and an HDR-dependent approach, we have previously reported successful reconstitution of OTC expression in up to

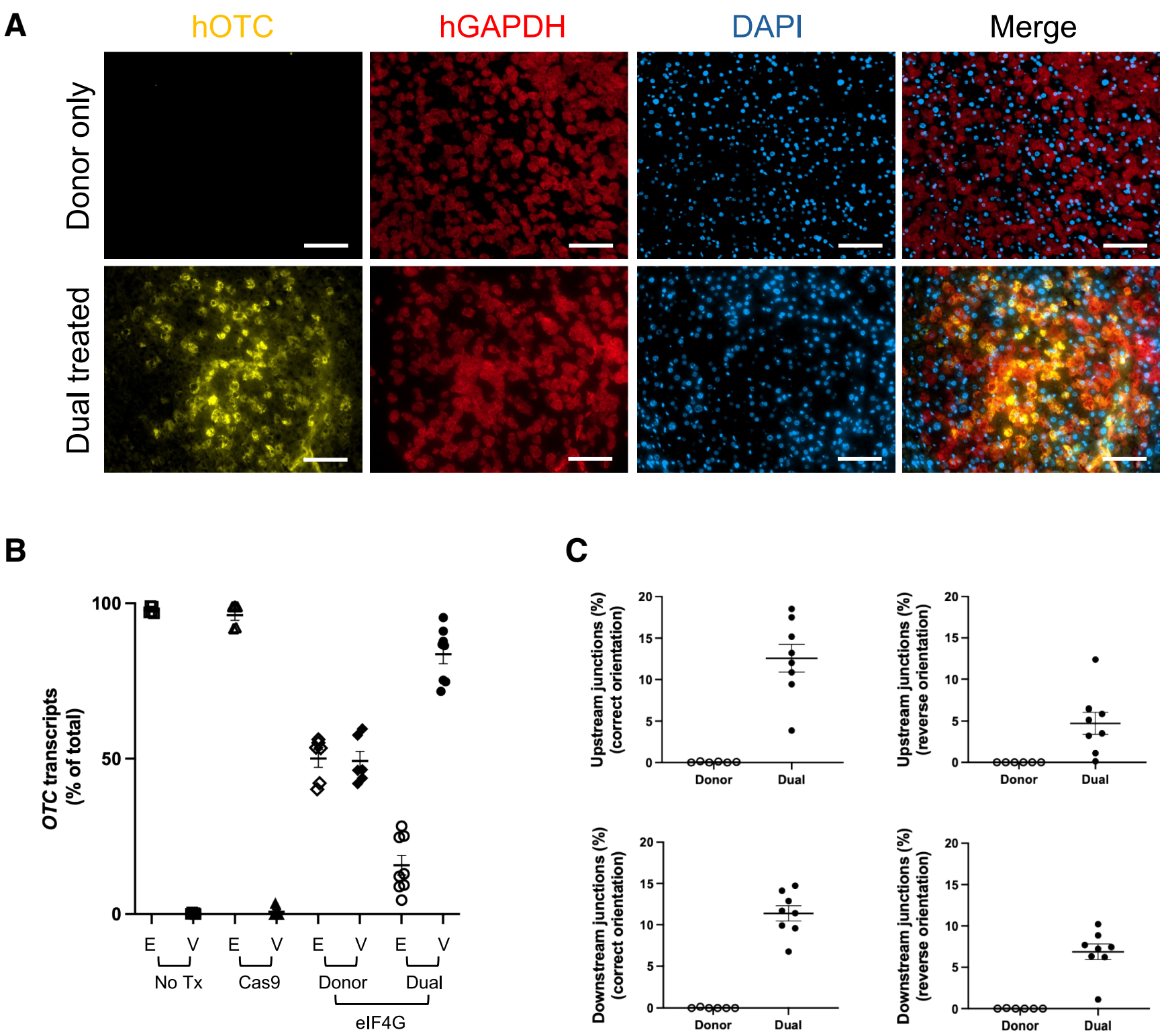

Figure 7. Efficient restoration of OTC expression in patient-derived primary human hepatocytes *in vivo*
(A) Restoration of OTC expression in chimeric liver sections 6 weeks following vector delivery. Animals received 5×10^{11} vg/mouse of the AAV vector containing the editing reagents and 1×10^{11} vg/mouse of the AAV donor. The donor construct contained a full-length human OTC cDNA downstream of a human eIF4G element packaged in the AAV-SYD12 capsid. Sections were labeled with antibodies against OTC (yellow) and human GAPDH (red) proteins. Nuclei were counter-stained with DAPI (blue). Scale bars represent 100 μm. (B) To quantitate the proportion of endogenous (E, open shapes) and vector-encoded (V, filled shapes) *OTC* transcripts, next-generation Illumina sequencing was performed on cDNA isolated from chimeric human-mouse livers transplanted with patient-derived human hepatocytes. (C) Quantification of predicted junctions between the human genome and the integrated donor was determined by using digital PCR of genomic DNA isolated from chimeric mouse-human livers. Editing events were quantified at both the 5′ and 3′ ends of the integrated donor cassette and in both the correct and reverse orientations, expressed as a percentage of copies of the human albumin locus. Samples from engrafted FRG mice that received the donor AAV vector only were used as controls (n = 6 for the donor-only treatment group and n = 8 for the dual-treatment group; mean ± SEM are shown).

29% of patient-derived primary human hepatocytes engrafted and expanded in the FRG mouse liver,[1] a result close to that achieved by Yang et al. using an equivalent approach in the newborn Spf^{ash} mouse where mutation reversion rates of up to 20% were observed.[36] In the later study, the mutation reversion rates achieved dropped to 0.2%–0.3% when the same experiments were performed in adult Spf^{ash} mice, indicative of the efficacy limitations imposed by the cell-cycle dependence of HDR-dependent approaches. This has led to a greater focus on homology-independent approaches for targeted integration of exogenous DNA that exploit alternate repair pathways.[38,40–42] Several earlier liver-focused studies have used homology-independent approaches to target intronic sequences in the murine albumin locus in non-cell-autonomous disease contexts including mucopolysaccharidosis type VI and hemophilia A and B.[38–40,43] The highest functional editing rate reported was 18%, and this was following neonatal administration.[40] This is similar to the mutation reversion rates achieved by Yang et al. in the newborn Spf^{ash} mouse using an HDR-dependent approach, albeit at a different target locus,[36] but it does not capture the impact of hepatocyte quiescence in the adult liver on the relative efficacy of these two editing approaches. Our targeted integration data and earlier HDR-dependent integration data published by Yang et al., both generated in the adult Spf^{ash} mouse model, allow direct comparison.[36] The differences in functional repair of the murine Otc locus are dramatic (24%–40% versus 0.2%–0.3%) with our targeted integration being greater than 100-fold more efficient. Although less dramatic, results from this and our previous study targeting the OTC locus in patient-derived primary human hepatocytes engrafted in the FRG mouse liver show the same hierarchy of effect, albeit blunted (47% versus 29%).[1] This blunted effect is likely the consequence of proliferative activity in engrafted human hepatocytes.

The relationship between AAV vector dose, liver physiology, and genome editing efficiency remains incompletely understood. In principle, vector administration could influence editing outcomes through mechanisms beyond simple increased delivery of editing components, including effects on hepatocellular homeostasis and turnover. While we cannot formally exclude such contributions, previous work from our group demonstrated that AAV8-mediated gene transfer is associated with a transient reduction rather than stimulation of hepatocellular proliferation in the murine liver.[44] Consistent with this, the Spf^{ash} liver is generally considered parenchymally normal and, like patients with OTC deficiency, does not exhibit the degree of ongoing liver injury or regeneration observed in other disease models.[45] In the present study, editing efficiency correlated with vector dose in both Spf^{ash} and wild-type mice, supporting the interpretation that improved delivery of the editing machinery is a primary determinant of editing outcomes rather than dose-dependent changes in hepatocyte turnover. Nevertheless, the potential contribution of vector dose-dependent effects on liver physiology cannot be completely excluded. Future studies directly assessing hepatocellular proliferation and markers of liver injury following

administration of genome editing vectors would be valuable to further define the interplay between vector dose, liver biology, and editing efficiency.

An important unresolved observation arising from the current study is that both the absolute number of transcripts originating from the Otc locus and restoration of Otc enzymatic activity in successfully edited hepatocytes in Otc-deficient mice did not reach wild-type levels. There are several possible explanations. Most notably, targeting intron 1 in the murine locus using a donor cassette containing human *OTC* exons 2 to 10 results in hybrid mouse-human transcripts that encode a hybrid mouse-human OTC protein that could plausibly have reduced transcript abundance and enzymatic activity. Clearly, this would not be an issue when using the same approach to target the human locus. Other possibilities include, but are not limited to, differing mRNA architecture in the 3′ UTR, reduced mRNA expression/processing efficiency from target loci with complex integration events, and possible disruption of undefined native locus enhancer effects on the endogenous murine Otc promoter. These effects, if present, might also occur when targeting the human locus, despite reconstitution of wild-type human OTC expression. In the context of severe OTC deficiency in humans, reconstitution of OTC expression to even 10% physiological levels would be sufficient for therapeutic effect provided sufficient hepatocytes are edited.[32,46,47] This said, further detailed studies in human hepatocytes will be required to dissect these possibilities, particularly if the approach described were to be used in target disease contexts requiring full or near full physiological reconstitution of mutant target gene function.

A particularly noteworthy observation in the current study was that the high functional editing rates achieved in both mouse and human hepatocytes markedly exceeded the number of precise edits that would be expected if bona fide HITI events were the underlying mechanism.[5] Based on molecular analysis of predicted 5′ and 3′ junctions between integrated donor DNA and host cell sequences adjacent to the targeted integration site, a crude estimate of the amplitude of this discrepancy was at a minimum of 3- to 10-fold across the different experiments performed and likely considerably higher. Limited whole-genome nanopore long-read sequencing data obtained from a male C57BL/6J mouse in which locus-specific editing resulted in expression of EGFP from an inserted DNA donor cassette in approximately 80% of hepatocytes provided insight into the molecular basis of this discrepancy. This identified 31 sequence reads that mapped to the targeted insertion site in intron 1 of the *Otc* gene (Figure S21A). Of these, 22 (71%) showed evidence of editing, with 14 (45.2%) of those containing DNA sequences derived from one or both AAV vectors used (Figure S22). Notably, only 2 (6.5%) of these locus-specific editing events contained a precise 5′ HITI junction that could have plausibly originated from a functionally competent integration event. Four (12.9%) fully sequenced integration events contained complete, correctly oriented EGFP open reading frames (ORFs), but in the context of complex concatemeric events that also included EGFP ORFs in the reverse orientation and, in three of the four instances, additional sequences derived from the Cas9-encoding vector. Given intronic targeting, the presence of splice donor and acceptors sites flanking the correctly oriented EGFP ORFs in these concatemeric events provides a probable explanation for the observed excess of functional edits over precise HITI events. The remaining 8 (25.8%) integration events at the target locus contained unintended sequences, including ITRs released by Cas9-mediated cleavage of the donor vector and sequences derived from the Cas9-encoding vector or concatemers of the donor vector in the reverse orientation.

A further limitation of our study is the restricted scope of the off-target analysis. Although *in silico* prediction provides a useful first-pass assessment of potential guide-dependent off-target sites, it does not capture all possible nuclease-induced lesions, nor does it comprehensively assess unintended vector integration. This limitation is important in the context of our finding that the Cas9 vector frequently integrates at the on-target cleavage site. These observations support the need for more comprehensive off-target and integration-site profiling in future studies, particularly as the platform is adapted toward clinical translation.

In humans, an infant treated as part of the iECURE-sponsored OTC-HOPE trial (NCT06255782) is showing early signs of clinical benefit with plasma ammonia and glutamine levels remaining within the normal range, and the participant weaned off nitrogen scavenger medications.[48] This trial uses dual AAV vectors to deliver an ARCUS nuclease targeting the *PCSK9* locus as a “safe harbor” in combination with a donor vector encoding the *OTC* transgene. In contrast to our study, the donor cassette contains a liver-specific promoter so that the *OTC* transgene is expressed not only when the desired integration event occurs, but also from random integration events and from episomal vector genomes. This makes it difficult to determine the level of targeting efficiency using this approach without further in-depth molecular analysis. In addition, based on our results and the observations of others,[49,50] long-term ARCUS nuclease expression would be a likely possibility if the AAV vector containing the editing reagents integrates into the target site or elsewhere in the genome.

Together, these data provide a basis for improving the efficiency and fidelity of locus repair strategies using targeted integration. These include reducing or eliminating integration of unintended competing DNA sequences at the target site such as ITRs cleaved from the donor vector, an unavoidable consequence of the classical HITI approach,[5] and sequences derived from the second vector encoding the Cas9 editing machinery. For liver targeting, the latter could be eliminated by use of Cas9-encoding mRNA and sgRNA sequences delivered using lipid nanoparticle technology. The former could be achieved by not cleaving the donor AAV vector but rather relying on the natural capacity of AAV genomes to undergo concatenation in head-to-head and/or tail-to-tail configurations.[51,52] This would ensure correct orientation of therapeutic donor sequences in the target intron ready for transcription and mRNA splicing.

Therapeutic vector genomes in the reverse orientation simply become additional intronic sequence. This is directly supported by studies in which AAV genomes inserted into the Ube3a-ATS transcript did not interfere with transcription, provided promoter elements in the reverse orientation or polyadenylation signals were not present.[53] Finally, a deeper understanding of cellular DNA repair mechanisms will enable more rational design of donor templates for use in targeted integration strategies. For example, tailoring the donor template to the specific repair pathways engaged at the genomic break site may improve integration efficiency and precision. Given that genome editing using HITI has been achieved in non-dividing cells such as neurons,[5] donor template insertion has largely been attributed to the NHEJ pathway in this context. However, the contribution of MMEJ cannot be excluded, because MMEJ has been implicated in DNA integration events at double-strand breaks for both genome editing systems[54–56] and viral genomes such as hepatitis B virus.[57]

In conclusion, this study demonstrates highly efficient and functionally consequential targeted integration into the OTC locus in both murine and human hepatocytes *in vivo*. Importantly, the efficiencies achieved were sufficient for phenotype correction in the *Spf*^{ash} mouse and would provide unequivocal therapeutic benefit in patients with OTC deficiency and a host of other genetic/metabolic disease phenotypes if recapitulated in the intact human liver. The findings presented are also significant at a mechanistic level, as the unprecedented functional editing efficacies achieved were largely independent of precise HITI events and highlighted elements of this approach requiring further development and even re-evaluation. Further improvements in efficacy and safety are likely to be readily achievable by delivery of editing machinery as RNA. This would remove the risk of constitutive Cas9 expression, eliminate the use of exogenous promoter-enhancer elements, and reduce competition with the therapeutic cassette for integration at the target locus. Other potential improvements will require focus on donor vector configuration, dosage, and timing of delivery relative to the editing machinery. Finally, one particularly interesting possibility, highlighted by preliminary analysis of integrant structure, is to explore whether the natural tendency of AAV vector genomes to undergo concatemerization can exploited therapeutically as an alternative to HITI.

MATERIALS AND METHODS

Plasmid construction

Donor templates for repair were synthesized by GenScript Biotech (Singapore) and cloned into a self-complementary AAV2 backbone.[58] For the CRISPR-Cas9 nuclease system, guides were designed by selecting 21-nt target sequences next to a 5′NNGRRT PAM in the first intron of either the murine *Otc* or human *OTC* genes. Oligonucleotides for each guide (Table S7) were annealed and cloned into the pX602-AAV-TBG::NLS-SaCas9-NLS-HA-OLLAS-bGHpA;U6::BsaI-sgRNA plasmid after digestion with the restriction endonuclease BsaI (New England Biolabs). The pX602-AAV-TBG::NLS-SaCas9-NLS-HA-OLLAS-bGHpA;U6::BsaI-sgRNA plasmid was obtained from Feng Zhang (Addgene plasmid #61593) and contains the Cas9 nuclease from *Staphylococcus aureus* (SaCas9) under the control of a human liver-specific TBG promoter in an AAV2 backbone.[14]

Cell culture

Human embryonic kidney 293 cells (HEK-293) were maintained in Dulbecco's modified Eagle medium (Sigma-Aldrich, St. Louis, MA) supplemented with 10% (v/v) fetal bovine serum (SERANA, Bunbury, WA, Australia) at 37°C in a humidified 5% CO_2-air atmosphere. Cells were mycoplasma free.

Vector production

Recombinant AAV vectors were produced in HEK-293 cells by triple transfection as previously described.[59] Vectors used to target murine hepatocytes were packaged in the AAV8 capsid using the plasmid construct p5E18-VD2/8 (courtesy of James M. Wilson, University of Pennsylvania). Vectors targeting primary human hepatocytes engrafted into FRG mice were packaged into NP59,[24] hu.Lvr02 and hu.Lvr06,[60] N496D,[61] or the SYD12[27] capsids.

Vector particles were purified by standard cesium chloride (CsCl) gradient centrifugation from both the cell lysate and supernatant precipitated using 40% (w/v) PEG-8000 in 2.5 M NaCl. Vector genome titers were assigned by digital droplet PCR (Bio-Rad, Berkeley, CA, USA) using EvaGreen supermix (Bio-Rad, Cat#1864034) and following the manufacturer's instructions using primers specific to the bovine growth hormone poly adenylation sequence (Table S7).

Mouse studies

All animal care and experimental procedures were evaluated and approved by the Animal Ethics Committee of the Children's Medical Research Institute and The Children's Hospital at Westmead. Otc-deficient *Spf*^{ash} mice (B6EiC3Sn *a/A-Otc*$^{spf\text{-}ash}$/J; 001811) were bred in-house from breeding pairs originally purchased from The Jackson Laboratory (Bar Harbor, ME). Adult male C57BL/6J mice were purchased as required from the Animal Resources Center (Perth, Australia). Animals were housed in standard boxes in a temperature-controlled environment and had free access to water and mouse chow containing 18.9% (w/w) protein (Glen Forrest Stockfeeders, Glen Forrest, WA, Australia). Vectors were administered by either intraperitoneal or tail vein injection, as indicated, at a dose of 1×10^{12} vg of the Cas9-containing vector (approximately 4×10^{13} vg/kg) and 5×10^{11} vg (approximately 2×10^{13} vg/kg) of the donor vector unless otherwise stated. For *Spf*^{ash} mice, urine was collected at designated time points for orotate analysis. To determine the efficacy of Otc correction, a subset of mice (n = 3 or 5 per treatment group) were subjected to a high-protein diet challenge (50% [w/w] protein; SF00-252, Glen Forrest Stockfeeders) for up to 3 days. After this time, blood was collected via cardiac puncture, and plasma ammonia levels were determined using the Sigma Ammonia Assay Kit (Cat # AA0100).

$Fah^{-/-}Rag2^{-/-}Il2rg^{-/-}$ (FRG) mice breeding pairs were obtained from the laboratory of Professor Markus Grompe (Oregon Stem Cell Center, Oregon Health and Science University, Portland,

Oregon) and bred in-house. As FRG mice are immunodeficient, they were housed in individually ventilated filter cages. Mice had free access to drinking water supplemented with 2-(2-nitro-4-trifluoromethylbenzoyl)-1,3-cyclohexanedione (NTBC) before engraftment. 8- to 12-week-old female mice were engrafted with male human hepatocytes, isolated from an OTC-deficient pediatric donor or purchased from Lonza (Basel, Switzerland), as described previously.[62] Hepatocytes from an OTC-deficient pediatric donor were obtained after informed consent and approval by the Sydney Children's Hospitals Network Human Ethics Committee (2024/ETH00845). Engrafted mice were cycled on and off NTBC to promote liver repopulation as described previously.[1] Blood was collected every 2 weeks and at the end of the experiment to measure the levels of human albumin, used as a marker to estimate the level of engraftment, in serum by enzyme-linked immunosorbent assay (Bethyl Laboratories, Montgomery, TX). 11 weeks after engraftment, mice were treated with recombinant AAV vectors packaged in human hepatotropic capsids. At this time, mice were on a water cycle, and circulating human albumin levels were between 0.2 and 2.0 mg/mL (Table S5). Vectors were administered by tail vein injection at a dose of 5×10^{11} vg of the Cas9-containing vector and 1×10^{11} vg of the donor vector unless otherwise stated. Mice were cycled for a further 6 weeks before being euthanized.

At the end of the experiment, livers were harvested, and tissue was either snap frozen in liquid nitrogen for molecular analyses or fixed in 4% (w/v) paraformaldehyde (PFA) for immunofluorescence as described below. Experiments were conducted in an unblinded manner.

Tissue fixation

Fixation of liver tissue for immunofluorescent microscopy was carried out in 4% (w/v) PFA. Following fixation of liver in 4% PFA overnight, the tissue was cryoprotected by incubating in changes of 10%, 20%, and 30% (w/v) sucrose in PBS for at least 4 h each at 4°C. The final change in 30% sucrose was performed overnight at 4°C. The liver pieces were then frozen in optimum cutting temperature (TissueTek; Sakura Finetek, Torrance, CA) in isopentane/liquid nitrogen and stored at −80°C.

Analysis of urinary orotic acid

Urine was collected by placing mice on wire mesh over Whatmann filter paper for a 24-h period. Ten circular punches (3 mm in diameter) per paper were randomly selected and eluted in 300 μL of molecular grade water. Samples were analyzed for orotate and normalized to creatinine, and both were measured using liquid chromatography/tandem mass spectrometry on the WATERS XEVO TQS (Waters Australia, Rydalmere, Australia). To measure orotate, 50 μL of each sample was transferred to a 96-well polypropylene v-base plate (Nunc, Rosklide, Denmark). An internal standard (100 μmol/L stock of stable isotope-labeled 1,3-$15N_2$ orotic acid; Cambridge Isotope laboratories, Andover, MA) was added to each sample (50 μL/well). Orotate was analyzed in negative ion mode by multiple reaction monitoring using the transitions 155.1 > 111.1 and 157.1 > 113.1 for native and stable isotope, orotic acid, respectively. To measure creatinine, 5 μL of each sample was transferred to a 96-well polypropylene v-base plate and diluted with 100 μL of water. An internal standard (88 μmol/L stock of stable isotope labeled D3 creatinine; Cambridge Isotope laboratories, Andover, MA) was added to each sample (5 μL/well). Creatinine was analyzed in a positive ion mode by multiple reaction monitoring using transitions 114.15 > 86.19 and 117.15 > 89.19 for native and stable isotope-creatinine, respectively.

Analysis of Otc enzymatic activity

Otc enzymatic activity in liver sections in situ

Otc activity in liver sections, fixed prior in 4% (w/v) PFA, was analyzed as previously described[63] with modifications. Cryosections (5 μm) were incubated in two changes of 0.05% lead nitrate-containing reaction buffer for 15 min each, washed with distilled water, and then reacted with ammonium sulfide solution for 1 min followed by further washing. Imaging was carried out using the Zeiss AxioImager M1 microscope with a ZeissMRc color camera and Zen software. The proportion of gene-modified cells was estimated using FIJI/ImageJ analysis software[64] with a thresholding function applied to distinguish the positively stained area. Particles below a given size (representing intracellular lead deposits rather than cells with activity staining) were discarded from the analysis. Subsequently, total positive area was measured in 6 images per mouse taken with a ×20 objective.

Otc enzymatic activity in liver lysates

Otc activity in liver lysates was determined as previously described,[63] modified for a 96-well plate format. The final reaction volume was scaled down to 200 μL containing 50 to 100 mg liver tissue.

Isolation of human hepatocytes by collagenase perfusion and FACS analysis

Mouse liver was perfused, and a single-cell suspension was obtained as described previously.[60] Following perfusion and collection of liver cells, live cells were pelleted at 650 × *g* for 10 min at 4°C, and the pellet was resuspended in FACS buffer ($PBS^{-/-}$ with 5% [v/v] FBS and 5 mM EDTA). For engrafted FRG mice, to distinguish between mouse liver cells and human hepatocytes, cells were labeled with PE-conjugated anti-mouse-H2Kb (BD Biosciences, Cat # 553570, clone AF6-88.5; 1:100), biotin-conjugated anti-human-HLA-ABC (eBioscience, San Diego, CA; Cat # 13-9983-82, clone W6/32; 1:100), and eFluor450-conjugated streptavidin (eBioscience; Cat # 48-4317-82; 1:500). To identify OTC-positive human hepatocytes, cells were labeled with a rabbit polyclonal antibody to human OTC (Sigma-Aldrich; Cat # HPA000243; 1:100) at 4°C overnight followed by a polyclonal donkey anti-rabbit Alexa Fluor 647 (Invitrogen, Cat # A32795; 1:400 dilution) at room temperature for 2 h. Flow cytometry was performed in the Flow Cytometry Facility, Westmead Institute for Medical Research, Westmead, NSW, Australia. The data were analyzed using FlowJo 7.6.1 (FlowJo).

Immunostaining and fluorescent imaging of liver sections

Frozen sections (5 μm) were permeabilized in methanol at $-20^{\circ}C$ and blocked using 10% (v/v) serum appropriate for the primary antibody (Sigma-Aldrich) and 10% (v/v) FBS in $PBS^{-/-}$. For C57BL/6J mice, sections were incubated with anti-GFP conjugated with DyLight 488 (Rockland Immunochemicals, Limerick, PA; Cat #600-141-215; 1:800 dilution) at room temperature for 1 h and anti-glutamine synthetase (OriGene, Rockville, MD; Cat # TA500700 clone OTI1F4; 1:100 dilution) for 1.5 h at room temperature, followed by a polyclonal donkey anti-mouse Alexa Fluor 594 (Invitrogen; Cat # A32744; 1:800 dilution) for 1 h at room temperature. For *Spf*^{ash} mice, sections were incubated with either a rabbit anti-human OTC polyclonal (Sigma-Aldrich; Cat # HPA000243; 1:100 dilution) or rabbit anti-mouse Otc monoclonal (Abcam, Cambridge, UK; Cat # ab203859; 1:60 dilution) antibody overnight at $4^{\circ}C$ followed by a donkey anti-rabbit Alexa Fluor 594 secondary (Invitrogen; Cat# A21207; 1:800) for 1 h at room temperature. This was followed by anti-glutamine synthetase as described above and then a donkey anti-mouse IgG (H + L) Alexa Fluor Plus 488 secondary (Invitrogen; Cat # A32766; 1:800 dilution) for 1 h at room temperature. For FRG mice engrafted with primary human hepatocytes, sections were incubated with either a rabbit monoclonal antibody to human GAPDH conjugated with Alexa Fluor 647 (Abcam; Cat# ab215227, clone EPR6256; 1:800 dilution) for 1 h at room temperature, goat anti-GFP conjugated with DyLight 488 (as above) for 1 h at room temperature, or a rabbit polyclonal antibody to human OTC for 1.5 h at room temperature followed by a polyclonal donkey anti-rabbit Alexa Fluor 594 (as above) at room temperature for 1 h. Image analysis was performed using a Zeiss Axio Imager Beta with ZEN 2 software (ZEN 2 version 3.7.97.000). To determine the percentage of Otc-positive hepatocytes in *Spf*^{ash} mice following dual-vector delivery, the number of positive hepatocytes were counted in at least 5 images per mouse taken with a ×20 objective using QuPath 0.5.1.

Molecular biology techniques

DNA extraction

DNA was purified from whole liver using standard phenol/chloroform extraction and ethanol precipitation. Briefly, approximately 200 mg of liver tissue was homogenized in 600 μL of lysis buffer (10 mM TRIS-Cl [pH 8], 0.1 M EDTA [pH8], 0.5% [w/v] SDS and 10 mg/mL RNase A) and incubated for 1 h at $37^{o}C$. Proteinase K was then added to a final concentration of 100 μg/mL and incubated at $56^{o}C$ overnight. The DNA was extracted twice using phenol:chloroform:isoamyl alcohol (25:24:1) followed by chloroform:isoamyl alcohol (24:1) to remove any residual phenol. DNA in the aqueous phase was then precipitated in 0.2 volumes of 10 M ammonium acetate and 2 volumes of absolute ethanol. After centrifugation, the DNA pellet was washed in 70% (v/v) ethanol, air dried, and resuspended in 10 mM Tris-Cl (pH 8.5). The DNA was quantitated using the Invitrogen QuBit HS dsDNA kit (Thermo Fisher Scientific) and used for further analyses.

RNA isolation, cDNA synthesis, and quantification of transcripts

Total RNA was isolated from 50 to 100 mg whole liver using the Invitrogen PureLink RNA Mini Kit (Thermo Fisher Scientific) with on-column DNase digestion. cDNA was synthesized from 500 ng of the purified RNA using the Invitrogen SuperScript IV First-strand synthesis system (Thermo Fisher Scientific). The resulting cDNA was diluted 1:10 in nuclease-free water and used for quantitative RT-PCR analysis or dPCR where indicated using primers specific to the endogenous murine Otc and human *OTC* transcripts and vector-encoded *OTC*, EGFP, and hybrid transcripts. Transcripts were normalized to albumin, with the cDNA diluted 1:100 for this reaction (Table S7).

Determination of vector genome copy number

Vector genome copy number was determined using dPCR using the Qiacuity EvaGreen PCR Kit (Qiagen, Hilden, Germany) following the manufacturer's instructions. To detect AAV genomes, primers specific for either the SaCas9, EGFP, or the human OTC donor were used (Table S7). Vector genomes were normalized to either human or mouse albumin copy number using primers depending on the species (Table S7).

Determination of expected genomic junctions

The percentage of expected genomic junctions was determined by dPCR using the Qiacuity Probe PCR Kit (Qiagen) as per the manufacturer's protocol with primers and probes (Table S7). Briefly, the numbers of upstream and downstream junctions in both orientations were determined using 200 ng of predigested DNA and an initial denaturation step at $95^{\circ}C$ for 2 min, followed by 45 cycles of $95^{\circ}C$ for 15 s and $62^{\circ}C$ for 30 s. Signals were acquired on the green channel (FAM probe) with exposure of 600 s and a gain of 8. Results were normalized to the reference gene (murine or human albumin), which was acquired on the yellow channel (HEX probe) with an exposure of 500 s and a gain of 5.

Indel analysis

The presence of small insertions and deletions (indels) spanning the CRISPR-Cas9 cleavage site for each sgRNA (Table S7) was identified in PCR amplicons using Synthego's online ICE Analysis tool (Synthego Performance Analysis, ICE Analysis. 2019. v3.0). Briefly, the region spanning each SaCas9 cleavage site was amplified from 300 ng total liver genomic DNA using Q5 High-Fidelity DNA Polymerase (New England Biolabs, Ipswich, MA). PCR products were purified by agarose gel electrophoresis and the Wizard SV Gel and PCR Clean-Up System (Promega Corporation, Madison, WI). Large insertions at the target site are not amplified using this approach. The purified products were sequenced by the Australian Genome Research Facility (AGRF, Westmead, NSW, Australia).

Off-target analysis

Potential off-target sites in the human genome were identified using GT-Scan.[65] The 150-bp region spanning the predicted off-target site was amplified with Q5 High-Fidelity DNA Polymerase (New England Biolabs) and purified as described above. Amplicons from both a treated and control mouse were mixed in equimolar ratios, and next-generation sequencing (NGS) library preparations and

sequencing using a 2 × 150 paired-end configuration (PE150) were performed by Genewiz (Suzhou, China) using an Illumina MiSeq instrument. The raw paired-end reads were subjected to quality filtering to eliminate low-quality sequences. Reads with a mean quality score below 20 were discarded using BBDuk from BBTools 39.06 (https://sourceforge.net/projects/bbmap/). Subsequently, the filtered reads were merged using BBMerge. The merged and filtered FASTQ files were then processed with a Python script (Python 3.9) (filter.py) to separate the reads based on their sequence identity. The reads were aligned to the human genome (GRCh38) using the BWA aligner.[66] The resulting BAM files were sorted and indexed using Samtools[67] before being filtered to retain alignments with a mismatch rate of 5 or fewer. Mutation efficiency was calculated using GOANA.[68]

Analysis of *OTC* transcripts using next-generation sequencing

The proportion of endogenous and vector-derived human transcripts in engrafted patient-derived primary human hepatocytes following treatment was determined by NGS analysis. Library preparations and sequencing using a 2 × 250 paired-end configuration (PE250) were performed by Genewiz (Suzhou, China) using an Illumina MiSeq instrument. A 311-bp region was amplified using Q5 High-Fidelity DNA Polymerase (New England Biolabs) using primers that were common to both the endogenous and vector-derived human transcripts (Table S7). The reverse primer was barcoded. The raw paired-end reads were subjected to quality filtering to eliminate low-quality sequences. Reads with a mean quality score below 20 were discarded using BBDuk from BBTools 39.06 (https://sourceforge.net/projects/bbmap/). The subsequent filtered reads were merged using BBMerge. The merged and filtered FASTQ files were then processed with a Python script (Python 3.9) to separate the reads following their barcodes. The barcode-identified reads were aligned to the human genome (GRCh38) and a vector genome using the BWA aligner.[66] The resulting BAM files were sorted and indexed using Samtools[67] before being filtered to retain alignments with a mismatch rate of 5 or fewer.

Whole-genome sequencing

Genomic DNA from whole liver (4 μg) was sheared using a Megaruptor shearing kit (E07010003, Diagenode SA. Seraing, Belgium) using speed setting 29 and the fragment distribution confirmed to be between 20 and 30 kb using a Tapestation genomic kit (Agilent, Santa Clara, CA). 3 μg of DNA was used for library preparation with an SQK-LSK114 kit (Oxford Nanopore Technologies, Oxford, United Kingdom). The library was loaded onto an R10.4.1 PromethION flow cell and sequenced on an ONT PromethION P48 device. Samples were run for a maximum duration of 72 hours, with 2 nuclease flushes and reloads performed during the run, to maximize sequencing yield with or without adaptive sampling to enrich for reads containing the target locus or vector sequences. Base calling used the Dorado Basecall Server (7.1.4 + d7df870c0) with the high accuracy (HAC) model. Base-called reads were aligned to the mouse genome (GRCm39) using minimap2 v2.26. Reads aligning to a 100-bp window centered on the expected CRISPR-Cas9 cleavage site (chrX:10121747- chrX:10121847) were extracted using Samtools v1.19[67] and the composition of each read determined manually using SnapGene software (Dotmatics, Boston, MA). Additional processing was performed using a custom Python script. Processing scripts are available at https://github.com/szsctt/dual_AAV_OTC_nanopore.

Statistical analyses

GraphPad Prism (version 10.4.1) was used to generate graphs and for statistical analysis. Unless otherwise stated, data are displayed as the mean of each sample, with error bars indicating the standard error of the mean (SEM). Differences between groups were determined using a one-way ANOVA followed by a Tukey's multiple comparisons test. p values <0.05 were considered significant.

DATA AND CODE AVAILABILITY

Whole-genome sequencing data have been deposited in the Sequence Read Archive (SRA) with BioProject ID number PRJNA1402129.

ACKNOWLEDGMENTS

This work was supported by an Ideas grant from the National Health and Medical Research Council of Australia (2019329) and a project grant from the Foundation For Children (2019-201). We wish to thank the Cytometry Facility of the Westmead Institute for Medical Research for help with sorting of human/murine hepatocytes and all members of the BioResources Facility of the Children's Medical Research Institute for their ongoing support in maintaining the lines used in this study and Mehtap Baserdem for collecting the filter papers required for orotate analysis. Biorender.com was used to generate the graphical abstract and Figures 1C, 1D, 3A, 5A, and S22 (https://BioRender.com/kxy62pr).

AUTHOR CONTRIBUTIONS

S.L.G. conceived, designed, and performed experiments, supervised the research, analyzed data, and wrote the manuscript; F.D., O.P.Y.C., S.F.Y., E.Z., B.D., and C.W.L. performed experiments and analyzed data; S.C., S.H.Y.L., and J.V. performed experiments; A.H.K. and S.S. analyzed data; S.C.C., conceived experiments; M.C.-C. and L.L., supplied critical reagents; I.E.A. jointly supervised the research, conceived and designed experiments, and wrote the manuscript.

DECLARATION OF INTERESTS

The authors declare no competing financial interests.

SUPPLEMENTAL INFORMATION

Supplemental information can be found online at https://doi.org/10.1016/j.ymthe.2026.06.044.

REFERENCES

1. Ginn, S.L., Amaya, A.K., Liao, S.H.Y., Zhu, E., Cunningham, S.C., Lee, M., Hallwirth, C.V., Logan, G.J., Tay, S.S., Cesare, A.J., et al. (2020). Efficient in *vivo* editing of OTC-deficient patient-derived primary human hepatocytes. JHEP Rep. *2*, 100065. https://doi.org/10.1016/j.jhepr.2019.100065.
2. Kok, C.Y., Cunningham, S.C., Kuchel, P.W., and Alexander, I.E. (2019). Insights into Gene Therapy for Urea Cycle Defects by Mathematical Modeling. Hum. Gene Ther. *30*, 1385–1394. https://doi.org/10.1089/hum.2019.053.
3. McCullough, B.A., Yudkoff, M., Batshaw, M.L., Wilson, J.M., Raper, S.E., and Tuchman, M. (2000). Genotype spectrum of ornithine transcarbamylase deficiency: Correlation with the clinical and biochemical phenotype. Am. J. Med. Genet. *93*, 313–319. https://doi.org/10.1002/1096-8628(20000814)93:4<313::aid-ajmg11>3.0.co;2-m.
4. Barzel, A., Paulk, N.K., Shi, Y., Huang, Y., Chu, K., Zhang, F., Valdmanis, P.N., Spector, L.P., Porteus, M.H., Gaensler, K.M., and Kay, M.A. (2015). Promoterless

gene targeting without nucleases ameliorates haemophilia B in mice. Nature *517*, 360–364. https://doi.org/10.1038/nature13864.

5. Suzuki, K., Tsunekawa, Y., Hernandez-Benitez, R., Wu, J., Zhu, J., Kim, E.J., Hatanaka, F., Yamamoto, M., Araoka, T., Li, Z., et al. (2016). In vivo genome editing via CRISPR/Cas9 mediated homology-independent targeted integration. Nature *540*, 144–149. https://doi.org/10.1038/nature20565.

6. Yao, X., Wang, X., Hu, X., Liu, Z., Liu, J., Zhou, H., Shen, X., Wei, Y., Huang, Z., Ying, W., et al. (2017). Homology-mediated end joining-based targeted integration using CRISPR/Cas9. Cell Res. *27*, 801–814. https://doi.org/10.1038/cr.2017.76.

7. Sfeir, A., and Symington, L.S. (2015). Microhomology-Mediated End Joining: A Back-up Survival Mechanism or Dedicated Pathway? Trends Biochem. Sci. *40*, 701–714. https://doi.org/10.1016/j.tibs.2015.08.006.

8. Truong, L.N., Li, Y., Shi, L.Z., Hwang, P.Y.H., He, J., Wang, H., Razavian, N., Berns, M.W., and Wu, X. (2013). Microhomology-mediated End Joining and Homologous Recombination share the initial end resection step to repair DNA double-strand breaks in mammalian cells. Proc. Natl. Acad. Sci. USA *110*, 7720–7725. https://doi.org/10.1073/pnas.1213431110.

9. Hustedt, N., and Durocher, D. (2017). The control of DNA repair by the cell cycle. Nat. Cell Biol. *19*, 1–9. https://doi.org/10.1038/ncb3452.

10. Musunuru, K., Grandinette, S.A., Wang, X., Hudson, T.R., Briseno, K., Berry, A.M., Hacker, J.L., Hsu, A., Silverstein, R.A., Hille, L.T., et al. (2025). Patient-Specific In Vivo Gene Editing to Treat a Rare Genetic Disease. N. Engl. J. Med. *392*, 2235–2243. https://doi.org/10.1056/NEJMoa2504747.

11. Kosicki, M., Tomberg, K., and Bradley, A. (2018). Repair of double-strand breaks induced by CRISPR–Cas9 leads to large deletions and complex rearrangements. Nat. Biotechnol. *36*, 765–771. https://doi.org/10.1038/nbt.4192.

12. Cullot, G., Boutin, J., Toutain, J., Prat, F., Pennamen, P., Rooryck, C., Teichmann, M., Rousseau, E., Lamrissi-Garcia, I., Guyonnet-Duperat, V., et al. (2019). CRISPR-Cas9 genome editing induces megabase-scale chromosomal truncations. Nat. Commun. *10*, 1136. https://doi.org/10.1038/s41467-019-09006-2.

13. Gebhardt, R. (1992). Metabolic zonation of the liver: Regulation and implications for liver function. Pharmacol. Ther. *53*, 275–354. https://doi.org/10.1016/0163-7258(92)90055-5.

14. Ran, F.A., Cong, L., Yan, W.X., Scott, D.A., Gootenberg, J.S., Kriz, A.J., Zetsche, B., Shalem, O., Wu, X., Makarova, K.S., et al. (2015). In vivo genome editing using Staphylococcus aureus Cas9. Nature *520*, 186–191. https://doi.org/10.1038/nature14299.

15. Gao, G.-P., Alvira, M.R., Wang, L., Calcedo, R., Johnston, J., and Wilson, J.M. (2002). Novel adeno-associated viruses from rhesus monkeys as vectors for human gene therapy. Proc. Natl. Acad. Sci. USA *99*, 11854–11859. https://doi.org/10.1073/pnas.182412299.

16. Stephenson, A.A., Nicolau, S., Vetter, T.A., Dufresne, G.P., Frair, E.C., Sarff, J.E., Wheeler, G.L., Kelly, B.J., White, P., and Flanigan, K.M. (2023). CRISPR-Cas9 homology-independent targeted integration of exons 1-19 restores full-length dystrophin in mice. Mol. Ther. Methods Clin. Dev. *30*, 486–499. https://doi.org/10.1016/j.omtm.2023.08.009.

17. Bengtsson, N.E., Tasfaout, H., Hauschka, S.D., and Chamberlain, J.S. (2021). Dystrophin Gene-Editing Stability Is Dependent on Dystrophin Levels in Skeletal but Not Cardiac Muscles. Mol. Ther. *29*, 1070–1085. https://doi.org/10.1016/j.ymthe.2020.11.003.

18. Wasala, N.B., Million, E.D., Watkins, T.B., Wasala, L.P., Han, J., Yue, Y., Lu, B., Chen, S.J., Hakim, C.H., and Duan, D. (2022). The gRNA vector level determines the outcome of systemic AAV CRISPR therapy for Duchenne Muscular Dystrophy. Hum. Gene Ther. *33*, 518–528. https://doi.org/10.1089/hum.2021.130.

19. Jungermann, K. (1988). Metabolic zonation of liver parenchyma. Semin. Liver Dis. *8*, 329–341. https://doi.org/10.1055/s-2008-1040554.

20. Cunningham, S.C., Kok, C.Y., Dane, A.P., Carpenter, K., Kizana, E., Kuchel, P.W., and Alexander, I.E. (2011). Induction and Prevention of Severe Hyperammonemia in the spfash Mouse Model of Ornithine Transcarbamylase Deficiency Using shRNA and rAAV-mediated Gene Delivery. Mol. Ther. *19*, 854–859. https://doi.org/10.1038/mt.2011.32.

21. Hodges, P.E., and Rosenberg, L.E. (1989). The spfash mouse: a missense mutation in the ornithine transcarbamylase gene also causes aberrant mRNA splicing. Proc. Natl. Acad. Sci. USA *86*, 4142–4146. https://doi.org/10.1073/pnas.86.11.4142.

22. Borman, A.M., Le Mercier, P., Girard, M., and Kean, K.M. (1997). Comparison of picornaviral IRES-driven internal initiation of translation in cultured cells of different origins. Nucleic Acids Res. *25*, 925–932. https://doi.org/10.1093/nar/25.5.925.

23. Wong, E.-T., Ngoi, S.-M., and Lee, C.G.L. (2002). Improved co-expression of multiple genes in vectors containing internal ribosome entry sites (IRESes) from human genes. Gene Ther. *9*, 337–344. https://doi.org/10.1038/sj.gt.3301667.

24. Paulk, N.K., Pekrun, K., Zhu, E., Nygaard, S., Li, B., Xu, J., Chu, K., Leborgne, C., Dane, A.P., Haft, A., et al. (2018). Bioengineered AAV Capsids with Combined High Human Liver Transduction In Vivo and Unique Humoral Seroreactivity. Mol. Ther. *26*, 289–303. https://doi.org/10.1016/j.ymthe.2017.09.021.

25. Chow, E.C.Y., Wang, J.Z.Y., Quach, H.P., Tang, H., Evans, D.C., Li, A.P., Silva, J., and Pang, K.S. (2016). Functional Integrity of the Chimeric (Humanized) Mouse Liver: Enzyme Zonation, Physiologic Spaces, and Hepatic Enzymes and Transporters. Drug Metab. Dispos. *44*, 1524–1535. https://doi.org/10.1124/dmd.116.070060.

26. Kaffe, E., Roulis, M., Zhao, J., Qu, R., Sefik, E., Mirza, H., Zhou, J., Zheng, Y., Charkoftaki, G., Vasiliou, V., et al. (2023). Humanized mouse liver reveals endothelial control of essential hepatic metabolic functions. Cell *186*, 3793–3809.e26. https://doi.org/10.1016/j.cell.2023.07.017.

27. Cabanes-Creus, M., Navarro, R.G., Zhu, E., Baltazar, G., Liao, S.H.Y., Drouyer, M., Amaya, A.K., Scott, S., Nguyen, L.H., Westhaus, A., et al. (2022). Novel human liver-tropic AAV variants define transferable domains that markedly enhance the human tropism of AAV7 and AAV8. Mol. Ther. Methods Clin. Dev. *24*, 88–101. https://doi.org/10.1016/j.omtm.2021.11.011.

28. Suchy, F.P., Karigane, D., Nakauchi, Y., Higuchi, M., Zhang, J., Pekrun, K., Hsu, I., Fan, A.C., Nishimura, T., Charlesworth, C.T., et al. (2025). Genome engineering with Cas9 and AAV repair templates generates frequent concatemeric insertions of viral vectors. Nat. Biotechnol. *43*, 204–213. https://doi.org/10.1038/s41587-024-02171-w.

29. Luqman, M.W., Jenjaroenpun, P., Spathos, J., Shingte, N., Cummins, M., Nimsamer, P., Ittner, L.M., Wongsurawat, T., and Delerue, F. (2025). Long read sequencing reveals transgene concatemerization and vector sequences integration following AAV-driven electroporation of CRISPR RNP complexes in mouse zygotes. Front. Genome *7*, 1582097.

30. Anzalone, A.V., Randolph, P.B., Davis, J.R., Sousa, A.A., Koblan, L.W., Levy, J.M., Chen, P.J., Wilson, C., Newby, G.A., Raguram, A., and Liu, D.R. (2019). Search-and-replace genome editing without double-strand breaks or donor DNA. Nature *576*, 149–157. https://doi.org/10.1038/s41586-019-1711-4.

31. Komor, A.C., Kim, Y.B., Packer, M.S., Zuris, J.A., and Liu, D.R. (2016). Programmable editing of a target base in genomic DNA without double-stranded DNA cleavage. Nature *533*, 420–424. https://doi.org/10.1038/nature17946.

32. Caldovic, L., Abdikarim, I., Narain, S., Tuchman, M., and Morizono, H. (2015). Genotype–Phenotype Correlations in Ornithine Transcarbamylase Deficiency: A Mutation Update. J. Genet. Genomics *42*, 181–194. https://doi.org/10.1016/j.jgg.2015.04.003.

33. Neto, E.V., Laranjeira, F., Quelhas, D., Ribeiro, I., Seabra, A., Mineiro, N., Carvalho, L.D.M., Lacerda, L., and Ribeiro, M.G. (2018). Mutation analysis of the *PAH* gene in phenylketonuria patients from Rio de Janeiro, Southeast Brazil. Mol. Genet. Genomic Med. *6*, 575–591.

34. Gillmore, J.D., Gane, E., Taubel, J., Kao, J., Fontana, M., Maitland, M.L., Seitzer, J., O'Connell, D., Walsh, K.R., Wood, K., et al. (2021). CRISPR-Cas9 In Vivo Gene Editing for Transthyretin Amyloidosis. N. Engl. J. Med. *385*, 493–502. https://doi.org/10.1056/NEJMoa2107454.

35. Cunningham, S.C., Kok, C.Y., Spinoulas, A., Carpenter, K.H., and Alexander, I.E. (2013). AAV-encoded OTC activity persisting to adulthood following delivery to newborn spfash mice is insufficient to prevent shRNA-induced hyperammonaemia. Gene Ther. *20*, 1184–1187. https://doi.org/10.1038/gt.2013.51.

36. Yang, Y., Wang, L., Bell, P., McMenamin, D., He, Z., White, J., Yu, H., Xu, C., Morizono, H., Musunuru, K., et al. (2016). A dual AAV system enables the Cas9-mediated correction of a metabolic liver disease in newborn mice. Nat. Biotechnol. *34*, 334–338. https://doi.org/10.1038/nbt.3469.

37. Wang, L., Yang, Y., Breton, C., Bell, P., Li, M., Zhang, J., Che, Y., Saveliev, A., He, Z., White, J., et al. (2020). A mutation-independent CRISPR-Cas9–mediated gene targeting approach to treat a murine model of ornithine transcarbamylase deficiency. Sci. Adv. *6*, eaax5701. https://doi.org/10.1126/sciadv.aax5701.

38. Esposito, F., Dell'Aquila, F., Rhiel, M., Auricchio, S., Chmielewski, K.O., Andrieux, G., Ferla, R., Horrach, P.S., Padmanabhan, A., Di Cunto, R., et al. (2024). Safe and effective liver-directed AAV-mediated homology-independent targeted integration in mouse models of inherited diseases. Cell Rep. Med. *5*, 101619. https://doi.org/10.1016/j.xcrm.2024.101619.

39. Chen, H., Shi, M., Gilam, A., Zheng, Q., Zhang, Y., Afrikanova, I., Li, J., Gluzman, Z., Jiang, R., Kong, L.-J., and Chen-Tsai, R.Y. (2019). Hemophilia A ameliorated in mice by CRISPR-based in vivo genome editing of human Factor VIII. Sci. Rep. *9*, 16838. https://doi.org/10.1038/s41598-019-53198-y.

40. Tornabene, P., Ferla, R., Llado-Santaeularia, M., Centrulo, M., Dell'Anno, M., Esposito, F., Marrocco, E., Pone, E., Minopoli, R., Iodice, C., et al. (2022). Therapeutic homology-independent targeted integration in retina and liver. Nat. Commun. *13*, 1963. https://doi.org/10.1038/s41467-022-29550-8.

41. Bloomer, H., Smith, R.H., Hakami, W., and Larochelle, A. (2021). Genome editing in human hematopoietic stem and progenitor cells via CRISPR-Cas9-mediated homology-independent targeted integration. Mol. Ther. *29*, 1611–1624. https://doi.org/10.1016/j.ymthe.2020.12.010.

42. Balke-Want, H., Keerthi, V., Gkitsas, N., Mancini, A.G., Kurgan, G.L., Fowler, C., Xu, P., Liu, X., Asano, K., Patel, S., et al. (2023). Homology-independent targeted insertion (HITI) enables guided CAR knock-in and efficient clinical scale CAR-T cell manufacturing. Mol. Cancer *22*, 100. https://doi.org/10.1186/s12943-023-01799-7.

43. He, X., Zhang, Z., Xue, J., Wang, Y., Zhang, S., Wei, J., Zhang, C., Wang, J., Urip, B.A., Ngan, C.C., et al. (2022). Low-dose AAV-CRISPR-mediated liver-specific knock-in restored hemostasis in neonatal hemophilia B mice with subtle antibody response. Nat. Commun. *13*, 7275. https://doi.org/10.1038/s41467-022-34898-y.

44. Dane, A.P., Cunningham, S.C., Kok, C.Y., Logan, G.J., and Alexander, I.E. (2015). Transient suppression of hepatocellular replication in the mouse liver following transduction with recombinant adeno-associated virus. Gene Ther. *22*, 917–922. https://doi.org/10.1038/gt.2015.66.

45. Yaplito-Lee, J., Chow, C.W., and Boneh, A. (2013). Histopathological findings in livers of patients with urea cycle disorders. Mol. Genet. Metab. *108*, 161–165. https://doi.org/10.1016/j.ymgme.2013.01.006.

46. Scharre, S., Posset, R., Garbade, S.F., Gleich, F., Seidl, M.J., Druck, A.-C., Okun, J.G., Gropman, A.L., Nagamani, S.C.S., Hoffmann, G.F., et al. (2022). Predicting the disease severity in male individuals with ornithine transcarbamylase deficiency. Ann. Clin. Transl. Neurol. *9*, 1715–1726. https://doi.org/10.1002/acn3.51668.

47. Cunningham, S.C., Spinoulas, A., Carpenter, K.H., Wilcken, B., Kuchel, P.W., and Alexander, I.E. (2009). AAV2/8-mediated Correction of OTC Deficiency Is Robust in Adult but Not Neonatal Spfash Mice. Mol. Ther. *17*, 1340–1346. https://doi.org/10.1038/mt.2009.88.

48. Baruteau, J., Cohn, G., Dhawan, A., Chakrapani, A., Grunewald, S., Abbott, M., Ashton, H., Foxall, S., Koh, A.L., Lazaridis, C., et al. (2025). P008: A first in human, single arm, open label phase 1/2 study evaluating ECUR-506 in neonatal OTC Deficiency: Initial Observations. Genet. Med. Open *3*, 102852. https://doi.org/10.1016/j.gimo.2025.102852.

49. Hanlon, K.S., Kleinstiver, B.P., Garcia, S.P., Zaborowski, M.P., Volak, A., Spirig, S.E., Muller, A., Sousa, A.A., Tsai, S.Q., Bengtsson, N.E., et al. (2019). High levels of AAV vector integration into CRISPR-induced DNA breaks. Nat. Commun. *10*, 4439. https://doi.org/10.1038/s41467-019-12449-2.

50. Miller, D.G., Petek, L.M., and Russell, D.W. (2004). Adeno-associated virus vectors integrate at chromosome breakage sites. Nat. Genet. *36*, 767–773. https://doi.org/10.1038/ng1380.

51. Duan, D., Sharma, P., Yang, J., Yue, Y., Dudus, L., Zhang, Y., Fisher, K.J., and Engelhardt, J.F. (1998). Circular Intermediates of Recombinant Adeno-Associated Virus Have Defined Structural Characteristics Responsible for Long-Term Episomal Persistence in Muscle Tissue. J. Virol. *72*, 8568–8577. https://doi.org/10.1128/JVI.72.11.8568-8577.1998.

52. Duan, D., Yan, Z., Yue, Y., and Engelhardt, J.F. (1999). Structural Analysis of Adeno-Associated Virus Transduction Circular Intermediates. Virology *261*, 8–14. https://doi.org/10.1006/viro.1999.9821.

53. Bazick, H.O., Mao, H., Niehaus, J.K., Wolter, J.M., and Zylka, M.J. (2024). AAV vector-derived elements integrate into Cas9-generated double-strand breaks and disrupt gene transcription. Mol. Ther. *32*, 4122–4137. https://doi.org/10.1016/j.ymthe.2024.09.032.

54. Nakade, S., Tsubota, T., Sakane, Y., Kume, S., Sakamoto, N., Obara, M., Daimon, T., Sezutsu, H., Yamamoto, T., Sakuma, T., and Suzuki, K.i.T. (2014). Microhomology-mediated end-joining-dependent integration of donor DNA in cells and animals using TALENs and CRISPR/Cas9. Nat. Commun. *5*, 5560. https://doi.org/10.1038/ncomms6560.

55. Yuan, B., Bi, C., Tian, Y., Wang, J., Jin, Y., Alsayegh, K., Tehseen, M., Yi, G., Zhou, X., Shao, Y., et al. (2024). Modulation of the microhomology-mediated end joining pathway suppresses large deletions and enhances homology-directed repair following CRISPR-Cas9-induced DNA breaks. BMC Biol. *22*, 101. https://doi.org/10.1186/s12915-024-01896-z.

56. Yao, X., Wang, X., Liu, J., Hu, X., Shi, L., Shen, X., Ying, W., Sun, X., Wang, X., Huang, P., and Yang, H. (2017). CRISPR/Cas9 – Mediated Precise Targeted Integration In Vivo Using a Double Cut Donor with Short Homology Arms. EBioMedicine *20*, 19–26. https://doi.org/10.1016/j.ebiom.2017.05.015.

57. Li, D., Wettengel, J.M., Ajoyan, H., Ho, V., Wu, G., Bae, S., Zhang, H., Boldbaatar, D., George, J., Douglas, M.W., and Tu, T. (2025). Hepatitis B viral DNA integration occurs within three days of infection and is enhanced by ATR inhibition. Tumour Virus Res. *20*, 200332. https://doi.org/10.1016/j.tvr.2025.200332.

58. Nathwani, A.C., Gray, J.T., Ng, C.Y.C., Zhou, J., Spence, Y., Waddington, S.N., Tuddenham, E.G.D., Kemball-Cook, G., McIntosh, J., Boon-Spijker, M., et al. (2006). Self-complementary adeno-associated virus vectors containing a novel liver-specific human factor IX expression cassette enable highly efficient transduction of murine and nonhuman primate liver. Blood *107*, 2653–2661. https://doi.org/10.1182/blood-2005-10-4035.

59. Cunningham, S.C., Dane, A.P., Spinoulas, A., Alexander, I.E., and Alexander, I.E. (2008). Gene delivery to the juvenile mouse liver using AAV2/8 vectors. Mol. Ther. *16*, 1081–1088. https://doi.org/10.1038/mt.2008.72.

60. Cabanes-Creus, M., Hallwirth, C.V., Westhaus, A., Ng, B.H., Liao, S.H.Y., Zhu, E., Navarro, R.G., Baltazar, G., Drouyer, M., Scott, S., et al. (2020). Restoring the natural tropism of AAV2 vectors for human liver. Sci. Transl. Med. *12*, eaba3312. https://doi.org/10.1126/scitranslmed.aba3312.

61. Cabanes-Creus, M., Westhaus, A., Navarro, R.G., Baltazar, G., Zhu, E., Amaya, A.K., Liao, S.H.Y., Scott, S., Sallard, E., Dilworth, K.L., et al. (2020). Attenuation of Heparan Sulfate Proteoglycan Binding Enhances In Vivo Transduction of Human Primary Hepatocytes with AAV2. Mol. Ther. Methods Clin. Dev. *17*, 1139–1154. https://doi.org/10.1016/j.omtm.2020.05.004.

62. Azuma, H., Paulk, N., Ranade, A., Dorrell, C., Al-Dhalimy, M., Ellis, E., Strom, S., Kay, M.A., Finegold, M., and Grompe, M. (2007). Robust expansion of human hepatocytes in Fah-/-/Rag2-/-/Il2rg-/- mice. Nat. Biotechnol. *25*, 903–910. https://doi.org/10.1038/nbt1326.

63. Ye, X., Robinson, M.B., Batshaw, M.L., Furth, E.E., Smith, I., and Wilson, J.M. (1996). Prolonged metabolic correction in adult ornithine transcarbamylase-deficient mice with adenoviral vectors. J. Biol. Chem. *271*, 3639–3646. https://doi.org/10.1074/jbc.271.7.3639.

64. Schindelin, J., Arganda-Carreras, I., Frise, E., Kaynig, V., Longair, M., Pietzsch, T., Preibisch, S., Rueden, C., Saalfeld, S., Schmid, B., et al. (2012). Fiji: an open-source platform for biological-image analysis. Nat. Methods *9*, 676–682. https://doi.org/10.1038/nmeth.2019.

65. O'Brien, A., and Bailey, T.L. (2014). GT-Scan: identifying unique genomic targets. Bioinformatics *30*, 2673–2675. https://doi.org/10.1093/bioinformatics/btu354.

66. Li, H., and Durbin, R. (2010). Fast and accurate long-read alignment with Burrows-Wheeler transform. Bioinformatics *26*, 589–595. https://doi.org/10.1093/bioinformatics/btp698.

67. Li, H., Handsaker, B., Wysoker, A., Fennell, T., Ruan, J., Homer, N., Marth, G., Abecasis, G., and Durbin, R. (2009). 1000 Genome Project Data Processing Subgroup. The Sequence Alignment/Map format and SAMtools. Bioinforma. Oxf. Engl. *25*, 2078–2079.

68. Reti, D., O'Brien, A., Wetzel, P., Tay, A., Bauer, D.C., and Wilson, L.O.W. (2021). GOANA: A Universal High-Throughput Web Service for Assessing and Comparing the Outcome and Efficiency of Genome Editing Experiments. CRISPR J. *4*, 243–252. https://doi.org/10.1089/crispr.2020.0068.

YMTHE, Volume *34*

Supplemental Information

Functional editing of the OTC locus by targeted integration with phenotype correction and restoration of endogenous expression patterns

Samantha L. Ginn, Fatemeh Doroudian, Sharntie Christina, Otilia P.Y. Chan, Caitlin W. Lucas, Erhua Zhu, Sile F. Yang, Beena Devanapalli, Anne H. Klein, Suzanne Scott, Joseph Vitale, Sharon C. Cunningham, Sophia H.Y. Liao, Marti Cabanes-Creus, Leszek Lisowski, and Ian E. Alexander

Table S1: *In silico* sgRNA selection using Benchling (mouse targeting)

	Sequence (5'to 3')	PAM	Specificity score	Efficiency score
sgRNA1	ACTGCCCATAGAGAAACAAGA	CTGAAT	87.7	35.3
sgRNA2	GCCAATTCTCTGTAGGCACCG	GGGGGT	76.9	47.4
sgRNA3	GAATACAATCCTGGGGCCAAT	GAGAAT	45.5	45.7

Table S2. Statistical analysis of Figure 2C (quantification of transcripts from the *Otc* promoter)

Endogenous transcripts

Tukey's multiple comparisons test	Mean diff.	95.00% CI of diff.	Below threshold?	Summary	Adjusted P Value
2:1 vs. 1:2	-22.65	-35.56 to -9.735	Yes	***	0.0001
2:1 vs. 1:1	-17.79	-30.71 to -4.879	Yes	**	0.0026
2:1 vs. 1:0.5	-8.614	-21.53 to 4.299	No	ns	0.3712
2:1 vs. 0.5:0.5	-37.33	-50.25 to -24.42	Yes	****	<0.0001
2:1 vs. Donor	-80.75	-93.67 to -67.84	Yes	****	<0.0001
2:1 vs. Cas9	-80.65	-93.57 to -67.74	Yes	****	<0.0001
1:2 vs. 1:1	4.856	-8.057 to 17.77	No	ns	0.8910
1:2 vs. 1:0.5	14.03	1.121 to 26.95	Yes	*	0.0265
1:2 vs. 0.5:0.5	-14.68	-27.60 to -1.771	Yes	*	0.0181
1:2 vs. Donor	-58.10	-71.02 to -45.19	Yes	****	<0.0001
1:2 vs. Cas9	-58.00	-70.92 to -45.09	Yes	****	<0.0001
1:1 vs. 1:0.5	9.178	-3.735 to 22.09	No	ns	0.3003
1:1 vs. 0.5:0.5	-19.54	-32.45 to -6.627	Yes	***	0.0008
1:1 vs. Donor	-62.96	-75.87 to -50.05	Yes	****	<0.0001
1:1 vs. Cas9	-62.86	-75.77 to -49.95	Yes	****	<0.0001
1:0.5 vs. 0.5:0.5	-28.72	-41.63 to -15.80	Yes	****	<0.0001
1:0.5 vs. Donor	-72.14	-85.05 to -59.22	Yes	****	<0.0001
1:0.5 vs. Cas9	-72.04	-84.95 to -59.12	Yes	****	<0.0001
0.5:0.5 vs. Donor	-43.42	-56.33 to -30.51	Yes	****	<0.0001
0.5:0.5 vs. Cas9	-43.32	-56.23 to -30.41	Yes	****	<0.0001
Donor vs. Cas9	0.1000	-12.81 to 13.01	No	ns	>0.9999

Test details	Mean 1	Mean 2	Mean diff.	SE of diff.	n1	n2	q	DF
2:1 vs. 1:2	19.20	41.85	-22.65	4.071	5	5	7.868	28
2:1 vs. 1:1	19.20	37.00	-17.79	4.071	5	5	6.181	28
2:1 vs. 1:0.5	19.20	27.82	-8.614	4.071	5	5	2.992	28
2:1 vs. 0.5:0.5	19.20	56.54	-37.33	4.071	5	5	12.97	28
2:1 vs. Donor	19.20	99.96	-80.75	4.071	5	5	28.05	28
2:1 vs. Cas9	19.20	99.86	-80.65	4.071	5	5	28.02	28
1:2 vs. 1:1	41.85	37.00	4.856	4.071	5	5	1.687	28
1:2 vs. 1:0.5	41.85	27.82	14.03	4.071	5	5	4.875	28
1:2 vs. 0.5:0.5	41.85	56.54	-14.68	4.071	5	5	5.101	28
1:2 vs. Donor	41.85	99.96	-58.10	4.071	5	5	20.19	28
1:2 vs. Cas9	41.85	99.86	-58.00	4.071	5	5	20.15	28
1:1 vs. 1:0.5	37.00	27.82	9.178	4.071	5	5	3.188	28
1:1 vs. 0.5:0.5	37.00	56.54	-19.54	4.071	5	5	6.788	28
1:1 vs. Donor	37.00	99.96	-62.96	4.071	5	5	21.87	28
1:1 vs. Cas9	37.00	99.86	-62.86	4.071	5	5	21.84	28
1:0.5 vs. 0.5:0.5	27.82	56.54	-28.72	4.071	5	5	9.977	28
1:0.5 vs. Donor	27.82	99.96	-72.14	4.071	5	5	25.06	28
1:0.5 vs. Cas9	27.82	99.86	-72.04	4.071	5	5	25.03	28
0.5:0.5 vs. Donor	56.54	99.96	-43.42	4.071	5	5	15.08	28
0.5:0.5 vs. Cas9	56.54	99.86	-43.32	4.071	5	5	15.05	28
Donor vs. Cas9	99.96	99.86	0.1000	4.071	5	5	0.03474	28

Number of families	1
Number of comparisons per family	21
Alpha	0.05

Hybrid transcripts

Tukey's multiple comparisons test	Mean diff.	95.00% CI of diff.	Below threshold?	Summary	Adjusted P Value
2:1 vs. 1:2	22.65	9.735 to 35.56	Yes	***	0.0001
2:1 vs. 1:1	17.79	4.879 to 30.71	Yes	**	0.0026
2:1 vs. 1:0.5	8.614	-4.299 to 21.53	No	ns	0.3712
2:1 vs. 0.5:0.5	37.33	24.42 to 50.25	Yes	****	<0.0001
2:1 vs. Donor	80.75	67.84 to 93.67	Yes	****	<0.0001
2:1 vs. Cas9	80.65	67.74 to 93.57	Yes	****	<0.0001
1:2 vs. 1:1	-4.856	-17.77 to 8.057	No	ns	0.8910
1:2 vs. 1:0.5	-14.03	-26.95 to -1.121	Yes	*	0.0265
1:2 vs. 0.5:0.5	14.68	1.771 to 27.60	Yes	*	0.0181
1:2 vs. Donor	58.10	45.19 to 71.02	Yes	****	<0.0001
1:2 vs. Cas9	58.00	45.09 to 70.92	Yes	****	<0.0001
1:1 vs. 1:0.5	-9.178	-22.09 to 3.735	No	ns	0.3003
1:1 vs. 0.5:0.5	19.54	6.627 to 32.45	Yes	***	0.0008
1:1 vs. Donor	62.96	50.05 to 75.87	Yes	****	<0.0001
1:1 vs. Cas9	62.86	49.95 to 75.77	Yes	****	<0.0001
1:0.5 vs. 0.5:0.5	28.72	15.80 to 41.63	Yes	****	<0.0001
1:0.5 vs. Donor	72.14	59.22 to 85.05	Yes	****	<0.0001
1:0.5 vs. Cas9	72.04	59.12 to 84.95	Yes	****	<0.0001
0.5:0.5 vs. Donor	43.42	30.51 to 56.33	Yes	****	<0.0001
0.5:0.5 vs. Cas9	43.32	30.41 to 56.23	Yes	****	<0.0001
Donor vs. Cas9	-0.1000	-13.01 to 12.81	No	ns	>0.9999

Test details	Mean 1	Mean 2	Mean diff.	SE of diff.	n1	n2	q	DF
2:1 vs. 1:2	80.80	58.15	22.65	4.071	5	5	7.868	28
2:1 vs. 1:1	80.80	63.00	17.79	4.071	5	5	6.181	28
2:1 vs. 1:0.5	80.80	72.18	8.614	4.071	5	5	2.992	28
2:1 vs. 0.5:0.5	80.80	43.46	37.33	4.071	5	5	12.97	28
2:1 vs. Donor	80.80	0.044	80.75	4.071	5	5	28.05	28
2:1 vs. Cas9	80.80	0.144	80.65	4.071	5	5	28.02	28
1:2 vs. 1:1	58.15	63.00	-4.856	4.071	5	5	1.687	28
1:2 vs. 1:0.5	58.15	72.18	-14.03	4.071	5	5	4.875	28
1:2 vs. 0.5:0.5	58.15	43.46	14.68	4.071	5	5	5.101	28
1:2 vs. Donor	58.15	0.044	58.10	4.071	5	5	20.19	28
1:2 vs. Cas9	58.15	0.144	58.00	4.071	5	5	20.15	28
1:1 vs. 1:0.5	63.00	72.18	-9.178	4.071	5	5	3.188	28
1:1 vs. 0.5:0.5	63.00	43.46	19.54	4.071	5	5	6.788	28
1:1 vs. Donor	63.00	0.044	62.96	4.071	5	5	21.87	28
1:1 vs. Cas9	63.00	0.144	62.86	4.071	5	5	21.84	28
1:0.5 vs. 0.5:0.5	72.18	43.46	28.72	4.071	5	5	9.977	28
1:0.5 vs. Donor	72.18	0.044	72.14	4.071	5	5	25.06	28
1:0.5 vs. Cas9	72.18	0.144	72.04	4.071	5	5	25.03	28
0.5:0.5 vs. Donor	43.46	0.044	43.42	4.071	5	5	15.08	28
0.5:0.5 vs. Cas9	43.46	0.144	43.32	4.071	5	5	15.05	28
Donor vs. Cas9	0.044	0.144	-0.1000	4.071	5	5	0.03474	28

Number of families	1
Number of comparisons per family	21
Alpha	0.05

Table S3. Statistical analysis of Figure 4F (whole liver Otc activity)

Tukey's multiple comparisons test	Mean diff.	95.00% CI of diff.	Below threshold?	Summary	Adjusted P Value
Spfash vs. Wild-type	-108.5	-128.8 to -88.26	Yes	****	<0.0001
Spfash vs. Cas9 only	-1.222	-21.49 to 19.04	No	ns	0.9998
Spfash vs. Donor only	-0.5983	-20.86 to 19.67	No	ns	>0.9999
Spfash vs. Dual	-6.989	-26.52 to 12.54	No	ns	0.8306
Wild-type vs. Cas9 only	107.3	87.04 to 127.6	Yes	****	<0.0001
Wild-type vs. Donor only	107.9	87.66 to 128.2	Yes	****	<0.0001
Wild-type vs. Dual	101.5	82.01 to 121.1	Yes	****	<0.0001
Cas9 only vs. Donor only	0.6233	-19.64 to 20.89	No	ns	>0.9999
Cas9 only vs. Dual	-5.768	-25.29 to 13.76	No	ns	0.9070
Donor only vs. Dual	-6.391	-25.92 to 13.14	No	ns	0.8710

Test details	Mean 1	Mean 2	Mean diff.	SE of diff.	n1	n2
Spf^{ash} vs. Wild-type	4.755	113.3	-108.5	6.920	6	6
Spf^{ash} vs. Cas9 only	4.755	5.977	-1.222	6.920	6	6
Spf^{ash} vs. Donor only	4.755	5.353	-0.5983	6.920	6	6
Spf^{ash} vs. Dual	4.755	11.74	-6.989	6.668	6	7
Wild-type vs. Cas9 only	113.3	5.977	107.3	6.920	6	6
Wild-type vs. Donor only	113.3	5.353	107.9	6.920	6	6
Wild-type vs. Dual	113.3	11.74	101.5	6.668	6	7
Cas9 only vs. Donor only	5.977	5.353	0.6233	6.920	6	6
Cas9 only vs. Dual	5.977	11.74	-5.768	6.668	6	7
Donor only vs. Dual	5.353	11.74	-6.391	6.668	6	7

Number of families	1
Number of comparisons per family	10
Alpha	0.05

Table S4: *In silico* sgRNA selection using Benchling (human targeting)

	Sequence (5'to 3')	PAM	Specificity score	Efficiency score
sgRNA1	CCTAAGCTACATAAAAATTAG	AAGAGT	43.8	43.6
sgRNA2	AAGTGAAGACTACTTAGACAG	TAGAGT	46.5	87.2
sgRNA3	TCTCAAATTCCTTGAAACAGA	GGGAGT	48.9	42.8

Table S5. Experimental details for studies using engrafted FRG mice

ID	Donor cells*	Cell number injected (million)	Capsid	Treatment	Human albumin level at time of injection (mg/mL)	Human albumin level at time of harvest (mg/mL)	Relevent Figure
Screening sgRNA efficiency in wild-type primary human hepatocytes							
253	#HUM4198	0.68			0.3	0.4	
258	#HUM4198	0.68			1.7	1.5	
618	#HUM4198	0.52	NP59	Cas9 only	0.2	0.6	Figure 5C
634	#HUM4198	0.52			0.4	1.5	
646	#HUM4198	0.52			0.4	0.7	
Evaluation of EGFP-containing donor vectors in wild-type primary human hepatocytes							
87	#HUM181141	0.44			0.9	1.2	
156	#HUM181141	0.44		Donor only (EGFP-	0.5	1.4	
382	#HUM181141	0.56		no IRES)	0.4	2.0	
550	#HUM182411	0.48			1.2	3.5	
60	#HUM4198	0.8			2.0	9.3	
61	#HUM4198	0.8			1.1	4.2	
191	#HUM181141	0.45		Dual (EGFP-	0.2	1.0	
208	#HUM181141	0.45		no IRES)	0.4	3.9	
606	#HUM181141	0.54			0.6	3.4	
620	#HUM181141	0.54			0.8	1.4	
501	Auxilary liver	0.54			0.5	0.6	
503	Auxilary liver	0.54		Donor only	0.2	0.2	
504	Auxilary liver	0.54		(EMCV-EGFP)	0.5	1.5	
505	Auxilary liver	0.54	NP59		0.9	0.5	Figure 5D-F
167	#HUM4198	0.6			0.4	3.1	
165	#HUM181141	0.45		Dual	0.6	2.5	
211	#HUM181141	0.45		(EMCV-EGFP)	0.2	1.7	
605	#HUM181141	0.54			0.9	2.2	
673	#HUM181141	0.8			0.6	2.8	
74	#HUM181141	0.44			0.4	1.3	
83	#HUM181141	0.44		Donor only	0.5	1.2	
289	Auxilary liver	0.9		(eIF4G-EGFP)	0.7	2.3	
305	Auxilary liver	0.9			0.4	1.9	
166	#HUM181141	0.45			0.2	1.1	
202	#HUM181141	0.45		Dual (eIF4G-	0.5	1.5	
355	#HUM182411	0.6		EGFP)	0.5	1.6	
477	#HUM4198	0.45			0.7	3.5	
699	#HUM181141	0.8			0.4	0.9	
Correction of OTC-deficient patient hepatocytes							
292		0.82			0.2	2.6	
527		0.82			0.2	2.9	
532		0.73	N/A	Untreated	0.6	2.7	
555		0.82			0.3	3.4	
591		0.82			0.1	0.8	
500		0.8			0.9	3.6	
506		0.8			0.5	2.7	
518		0.8		Cas9 only	0.4	2.5	
557		0.82			0.3	2.0	
589		0.82			0.4	2.6	
482	OTC patient (male)	0.82			0.3	1.2	
515	Exon 2 mutation:	0.82			0.5	1.1	Figure 7
537	c.140 A>T, p. Asn47Ile	0.82		Donor only	1.1	2.3	
575		0.82		(eIF4G-hOTC)	0.6	1.9	
580		0.82	SYD-12		1.3	5.7	
582		0.82			1.2	3.4	
246		0.65			0.6	0.7	
253		0.65			1.0	1.5	
480		0.82			1.0	1.3	
481		0.82		Dual (eIF4G-	1.2	1.4	
524		0.82		hOTC)	0.9	1.5	
536		0.82			0.5	0.4	
556		0.82			0.8	1.3	
581		0.82			0.4	0.6	

#HUM4198, #HUM181141 and #HUM182411 are wild-type primary human hepatocytes obtained from Lonza. Wild-type cells were also obtained from The Children's Hospital at Westmead
om an auxilary liver. OTC-deficient primary human hepatocytes were obtained from a patient undergoing liver transplantation at The Children's Hospital at Westmead. All donor cells are male

Table S6. Summary of reads at the Cas-9 cleavage site of the murine *Otc* locus

Sequencing strategy*	read_id	classification	Host contig	mapping_start	mapping_end	overlap_start	overlap_end	mapq	identity	indel_count	indel_details	class assigned
WGS	058c688c-955a-4069-b00b-3eec154046c5	single_junction	chrX	10114775	10121772	10121747	10121772	60	0.985	0	NA	ITR only
WGS	1a4dca7d-5567-4805-a24f-a5a14a92c85a	indel	chrX	10109608	10122616	10121747	10121847	60	0.9574	3	del:3@10121793-10121795;del:1@10121797-10121797;del:1@10121800-10121800	indel
WGS	28fca91a-3b61-4175-bb77-815501900b8a	single_junction	chrX	10118172	10121806	10121747	10121806	60	0.9819	1	del:4@10121793-10121796	ITR only
WGS	2a78a26d-3454-48a6-b674-1568702b6bbd	double_junction	chrX	10118589	10128165	10121747	10121847	60	0.9816	2	ins:1@10121798;ins:93@10121800	ITR only
WGS	2c213eb0-8409-4588-902d-199f53b6d6ba	indel	chrX	10118498	10128726	10121747	10121847	60	0.9886	1	del:3@10121797-10121799	indel
WGS	2f5d323a-a813-4f42-8308-de8f103b18df	single_junction	chrX	10118537	10121798	10121747	10121798	60	0.993	0	NA	Cas9 only
WGS	3c92c559-63fc-48bd-9470-64779fe12156	indel	chrX	10121457	10126023	10121747	10121847	60	0.9969	1	del:2@10121795-10121796	indel
WGS	4252cdc3-51a2-47b7-a180-f7b774077ac5	unmodified	chrX	10114668	10136273	10121747	10121847	60	0.9759	0	NA	unmodified
WGS	44ac8e95-9204-4b24-83cb-1f70ed923c3d	unmodified	chrX	10119200	10135941	10121747	10121847	60	0.9936	0	NA	unmodified
WGS	66c626e8-eb6d-4155-bdd2-8ae42d3cc84c	unmodified	chrX	10121195	10131411	10121747	10121847	60	0.986	0	NA	unmodified
WGS	82a0084c-07a4-47de-9c64-a6c58b178236	single_junction	chrX	10120255	10121798	10121747	10121798	60	0.9916	0	NA	Cas9 only
WGS	9ce04493-5da0-4d02-8a2b-adf502452acd	unmodified	chrX	10117706	10124073	10121747	10121847	60	0.9874	0	NA	unmodified
WGS	b9f2da27-2137-4bf3-8dec-b7d6c70404b2	single_junction	chrX	10119693	10121783	10121747	10121783	60	0.9852	0	NA	HITI event
WGS	c51a40aa-7bc7-412f-b119-5b6bb995fcb4	single_junction	chrX	10118819	10121797	10121747	10121797	60	0.9826	0	NA	concatemer
WGS	c8ec8b11-5189-41be-9f50-99289c0a5853	unmodified	chrX	10121669	10122049	10121747	10121847	60	0.9869	0	NA	unmodified
WGS	c9d3ba09-c89b-4594-adee-751355f934a0	single_junction	chrX	10121845	10122469	10121845	10121847	60	0.9968	0	NA	ITR only
WGS	da8b0b5b-4115-4fc2-bbe7-aabc5469d144	single_junction	chrX	10121799	10126030	10121799	10121847	60	0.9854	0	NA	concatemer
WGS	ec94f8f7-daef-4889-864d-9937c3deb117	other	chrX	10121838	10127149	10121838	10121847	60	0.9617	0	NA	downstream of cut site, not included in analysis
AS	022cfe96-73a0-4faa-8519-5c6aab1a658a	unmodified	chrX	10051107	10123312	10121747	10121847	60	0.9777	0	NA	unmodified
AS	0bae4216-eb6f-4e0e-89ce-4a517621ec04	double_junction	chrX	10099710	10129649	10121747	10121847	60	0.7733	1	ins:408@10121797	concatemer
AS	23d4f159-5694-4fcd-985e-898a463a41bc	indel	chrX	10121755	10146410	10121755	10121847	60	0.9834	1	ins:1@10121798	indel
AS	26f1df09-933a-4665-aa09-51f7016b03c2	unmodified	chrX	10114923	10147152	10121747	10121847	60	0.9636	0	NA	unmodified
AS	3a9c63cb-b83d-458c-b25a-66bc65a64856	single_junction	chrX	10097065	10121797	10121747	10121797	60	0.9743	0	NA	concatemer
AS	455c8907-cacd-48a6-8b94-d984dcbf9cd5	single_junction	chrX	10121798	10136889	10121798	10121847	60	0.9652	0	NA	ITR only
AS	535b21db-652d-4b99-b36b-0aab3a251e7b	unmodified	chrX	10120898	10129853	10121747	10121847	60	0.9837	0	NA	unmodified
AS	982a0e59-da14-4afc-ab37-01c709ba602e	indel	chrX	10120447	10122777	10121747	10121847	60	0.9854	2	del:1@10121793-10121793;del:1@10121797-10121797	indel
AS	9bd12fde-db60-4036-9717-62fb3fb878a7	indel	chrX	10117760	10123606	10121747	10121847	60	0.9867	2	del:1@10121793-10121793;ins:1@10121800	indel
AS	a447265f-4e53-424b-afec-97afee86baef	single_junction	chrX	10121797	10126255	10121797	10121847	60	0.9605	0	NA	concatemer
AS	b9bc71fb-0f60-41ce-891e-741e33f592de	single_junction	chrX	10121800	10122076	10121800	10121847	60	0.9819	0	NA	cut site not detected, not included in analysis
AS	d6991770-8b9c-4ec6-a7c9-691f5109a0a4	single_junction	chrX	10121798	10159968	10121798	10121847	60	0.9766	0	NA	HITI event
AS	f3a77775-c158-4fd9-a202-b9ff83aaabe0	unmodified	chrX	10121797	10126255	10121797	10121847	60	0.975	0	NA	unmodified
AS	f6e6a744-ce1a-4ba9-8263-debb97bc1844	indel	chrX	10105026	10134004	10121747	10121847	60	0.993	1	del:5@10121792-10121796	indel
AS	f8609b57-d5ea-4755-bda2-238e4823d9e5	indel	chrX	10121115	10128075	10121747	10121847	60	0.9843	1	del:1@10121793-10121793	indel

*WGS, whole genome sequencing; AS, WGS with adaptive sampling

Table S7. Primers used in this study

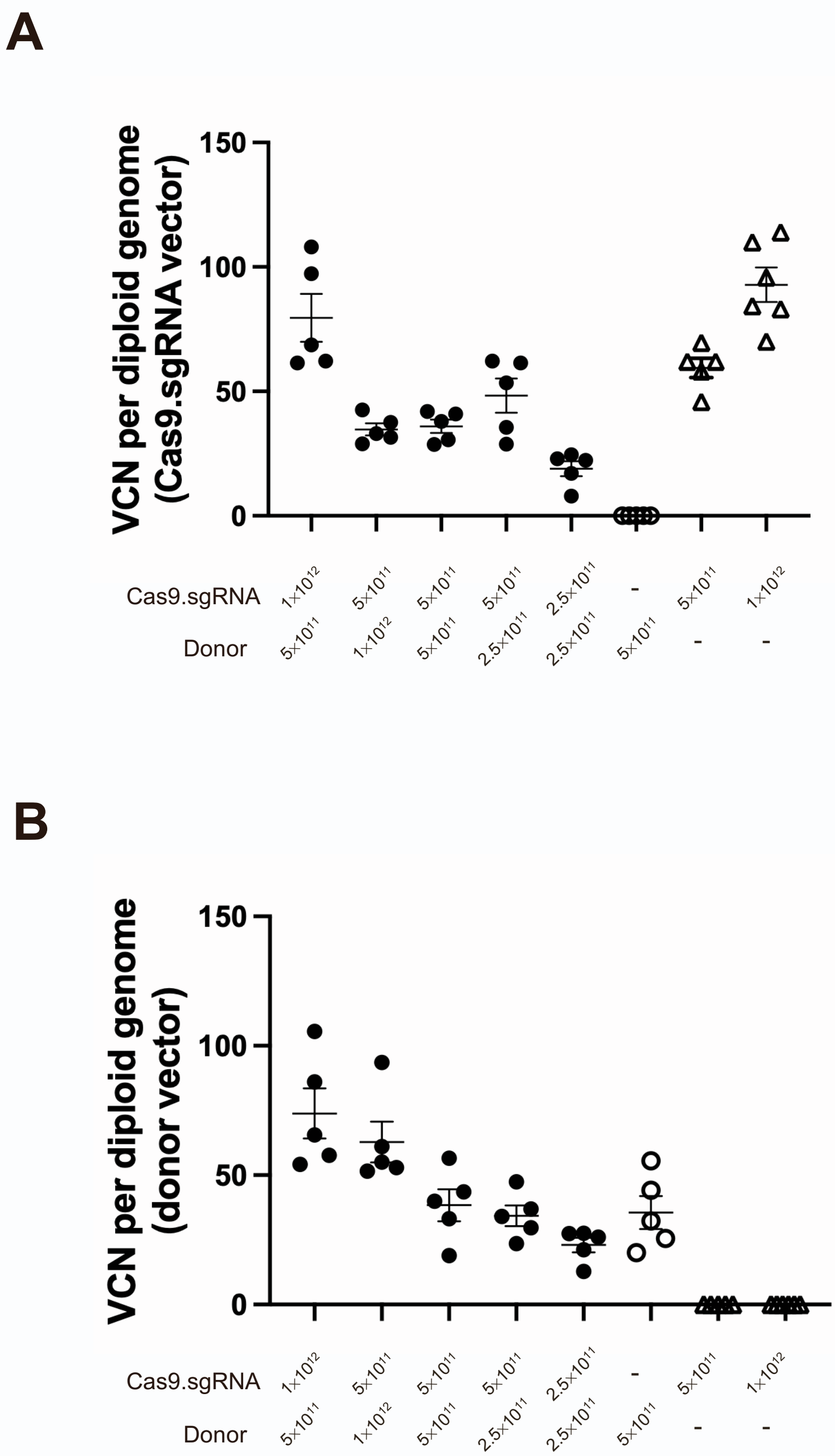

Figure S1.

Quantification of vector copy number (VCN) following dual AAV delivery in C57Bl6 mice. Vector copy number of (A) the SaCas9 and (B) EGFP transgenes per diploid genome in genomic DNA isolated from whole liver were determined by digital PCR (n=5 per treatment group, mean ± SEM are indicated). Doses of the SaCas9.sgRNA and EGFP-containing donor vectors are shown below each graph.

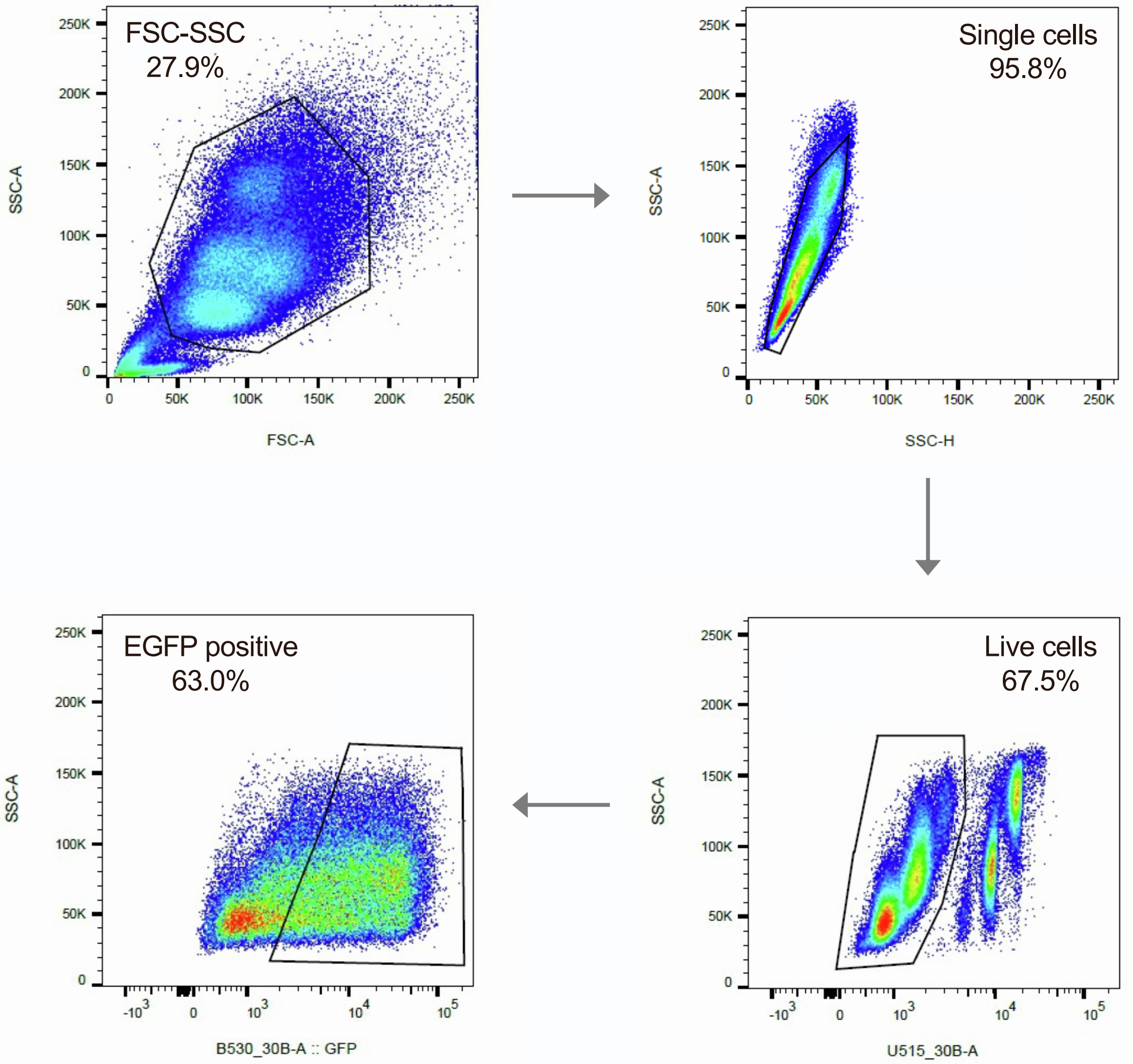

Figure S2.
Analysis of EGFP expression in the murine liver. Representative FACS profiles with gating strategy showing the percentage of EGFP positive hepatocytes 4 weeks after dual AAV delivery with 5×10^{11} vg of each vector. FSC, forward scatter; SSC, side scatter.

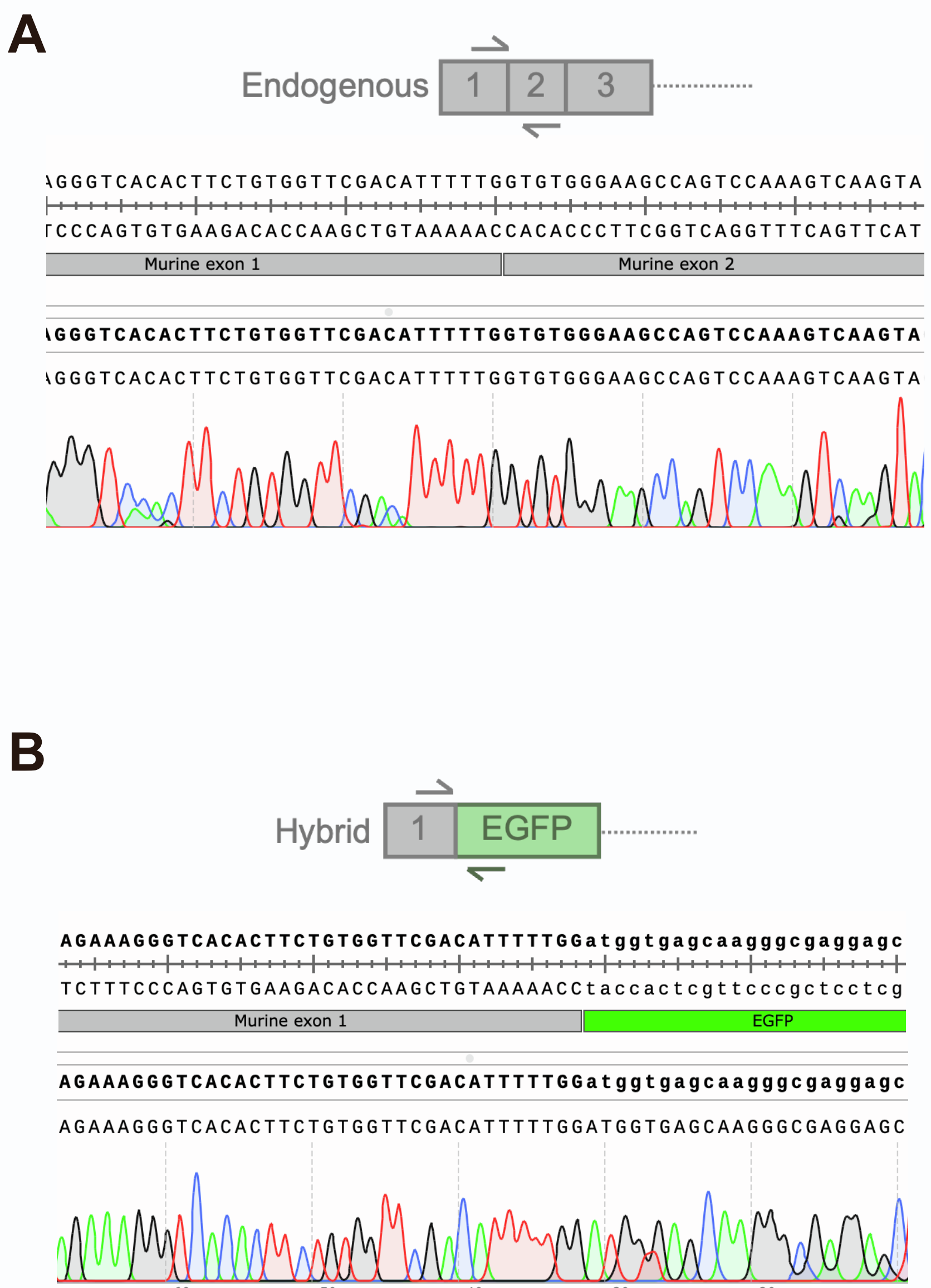

Figure S3.
Identification of transcripts originating from the *Otc* promoter. (A) endogenous transcripts and (B) hybrid transcripts between murine *Otc* exon 1 and EGFP. Junctions were confirmed in cDNA by using PCR primers that span the predicted junction followed by Sanger sequencing of the amplicons.

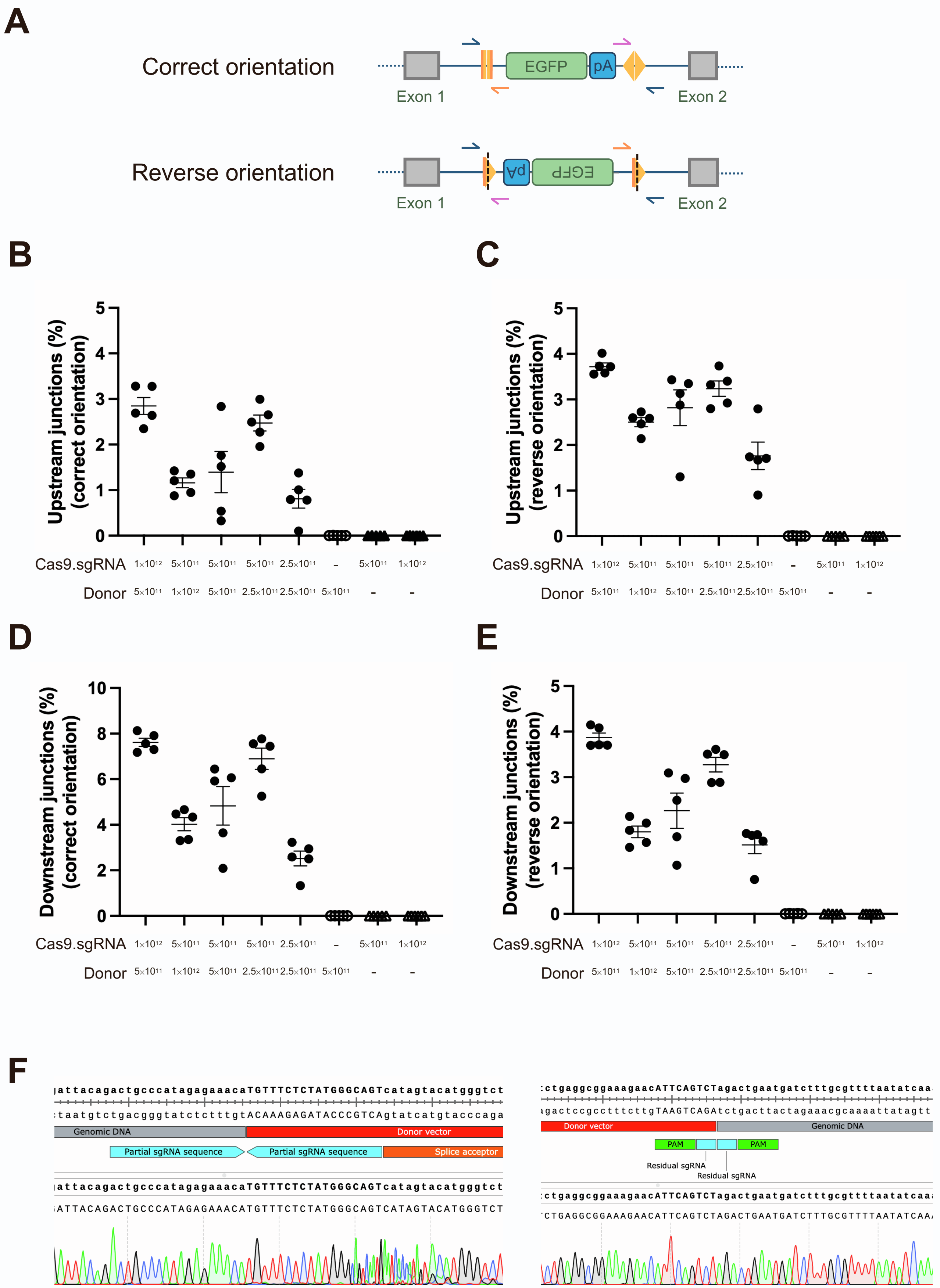
A
Correct orientation
EGFP
pA
Exon 1
Exon 2
Reverse orientation
Exon 1
Exon 2
B
Upstream junctions (%) (correct orientation)
C
Upstream junctions (%) (reverse orientation)
D
Downstream junctions (%) (correct orientation)
E
Downstream junctions (%) (reverse orientation)
Cas9.sgRNA
Donor
F
Genomic DNA
Donor vector
Partial sgRNA sequence
Partial sgRNA sequence
Splice acceptor
PAM
PAM
Residual sgRNA
Residual sgRNA

Figure S4.

Quantification of precise HITI junctions between the murine genome and integrated EGFP donor. (A) Diagrammatic representation of predicted upstream and downstream integration events with locations of primers indicated. pA, bovine growth hormone polyadenylation signal. Yellow arrow heads indicate the location of the sgRNA sequence and orange rectangles the protospacer adjacent motif (PAM). (B-E) Genomic DNA was isolated from whole liver 4 weeks after vector delivery and predicted junctions into the target locus were quantified by digital PCR (n=5 per treatment group, mean ± SEM are indicated). (F) The identify of upstream and downstream junctions were confirmed by PCR using primers that span the predicted junction followed by Sanger sequencing of the amplicons.

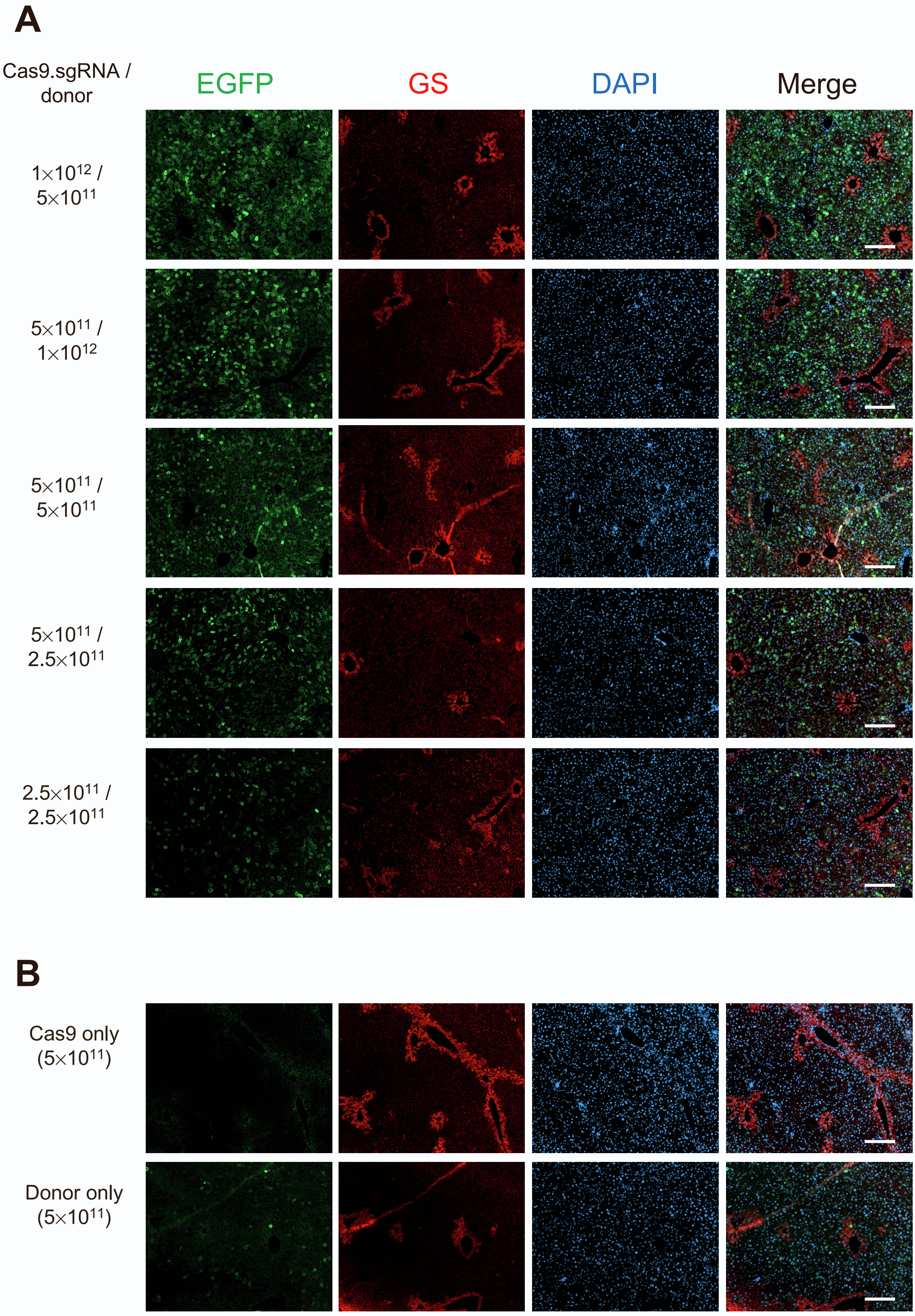
A
Cas9.sgRNA / donor
EGFP
GS
DAPI
Merge
1×10^12 / 5×10^11
5×10^11 / 1×10^12
5×10^11 / 5×10^11
5×10^11 / 2.5×10^11
2.5×10^11 / 2.5×10^11
B
Cas9 only (5×10^11)
Donor only (5×10^11)

Figure S5.

Physiological expression patterns following targeted integration are observed over a range of vector doses. (A) Reporter gene expression following integration of the donor template in murine hepatocytes shows metabolic zonation and (B) single vector treated controls. Sections were labelled with antibodies against the EGFP (green) and glutamine synthetase (GS, red) proteins and nuclei were counter-stained with DAPI (blue). Doses of the SaCas9.sgRNA and EGFP-containing donor vectors are shown to the right of the images. Scale bars = 200 μm.

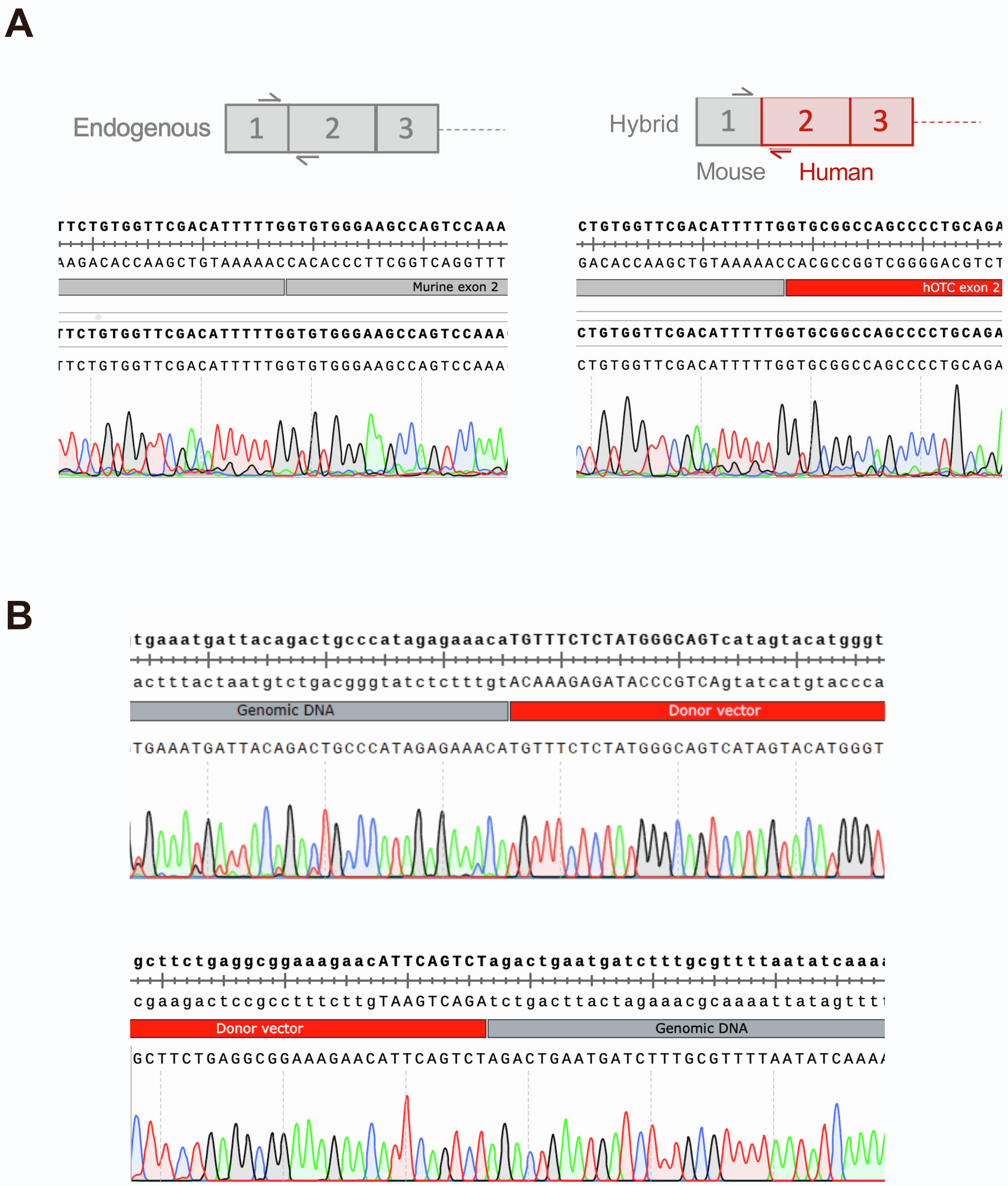

Figure S6.
Identification of predicted upstream and downstream HITI junctions between the murine genome and integrated OTC donor in (A) transcripts and (B) genomic DNA. Junctions were confirmed by PCR using primers that span the predicted junction followed by Sanger sequencing of the amplicons.

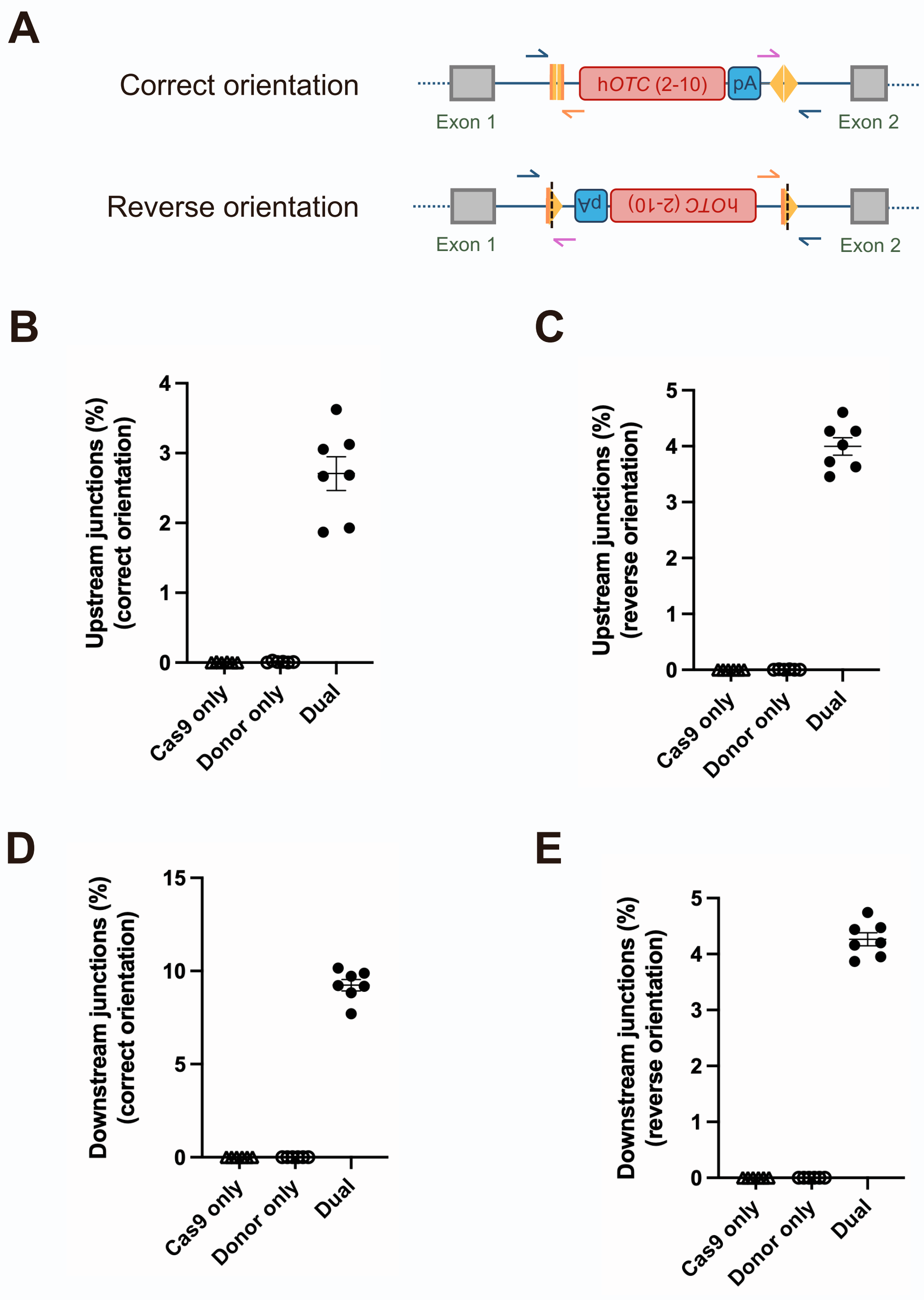

Figure S7.

Quantification of precise HITI junctions between the murine genome and integrated OTC donor. (A) Diagrammatic representation of predicted upstream and downstream integration events with locations of primers indicated. pA, bovine growth hormone polyadenylation signal. Yellow arrow heads indicate the location of the sgRNA sequence and orange rectangles the protospacer adjacent motif (PAM). (B-E) Genomic DNA was isolated from whole liver 8 weeks after vector delivery and predicted junctions into the target locus were quantified by digital PCR (n=6-7 per treatment group, mean ± SEM are indicated).

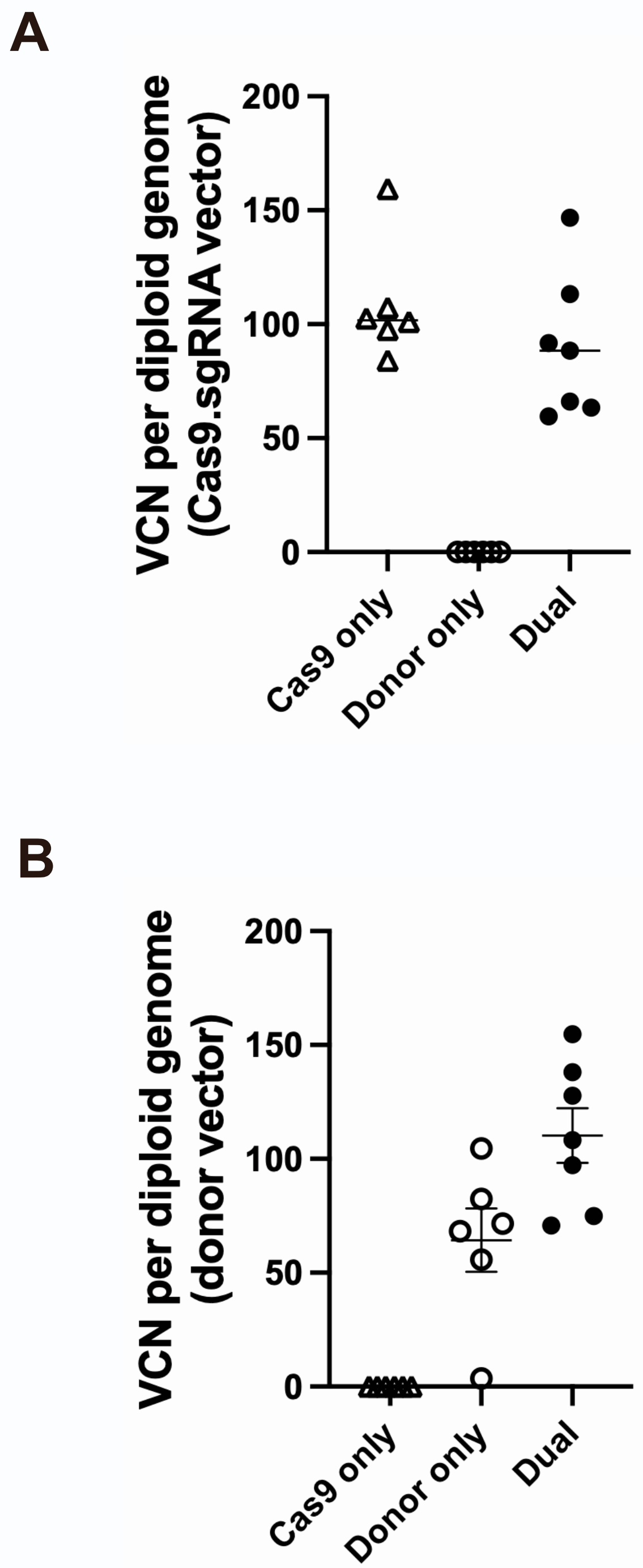

Figure S8.
Quantification of vector copy number (VCN) following dual AAV delivery in *Spf*^{ash} mice. Vector copy number of (A) the SaCas9 and (B) *OTC* transgenes per diploid genome in genomic DNA isolated from whole liver 8 weeks after vector delivery were determined by digital PCR (n=6-7 per treatment group, mean ± SEM are indicated). Animals receiving either the donor or editing reagents were included as controls.

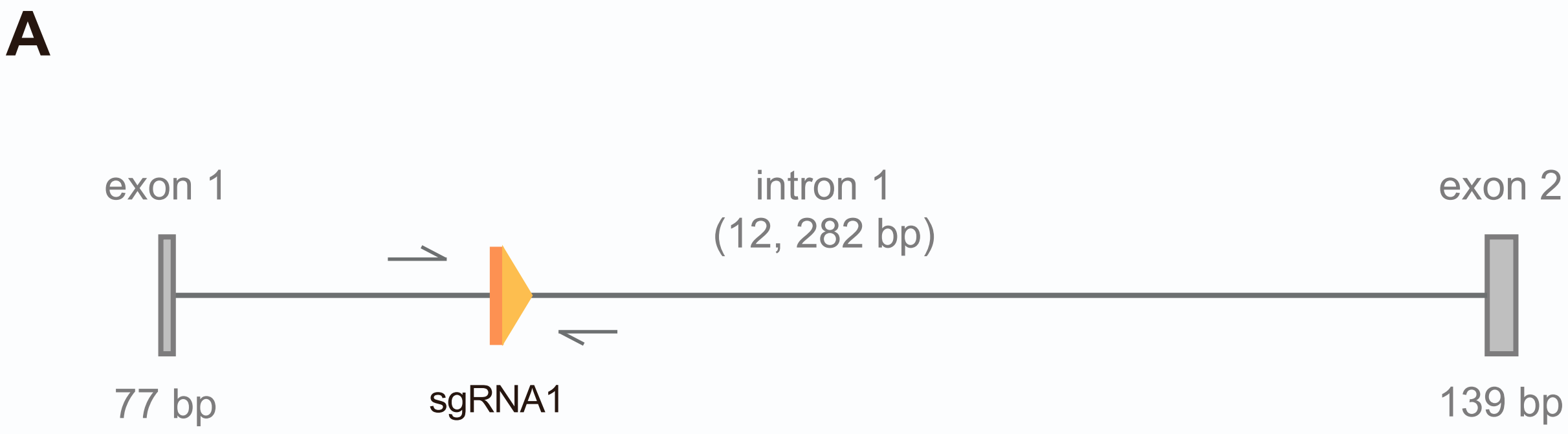

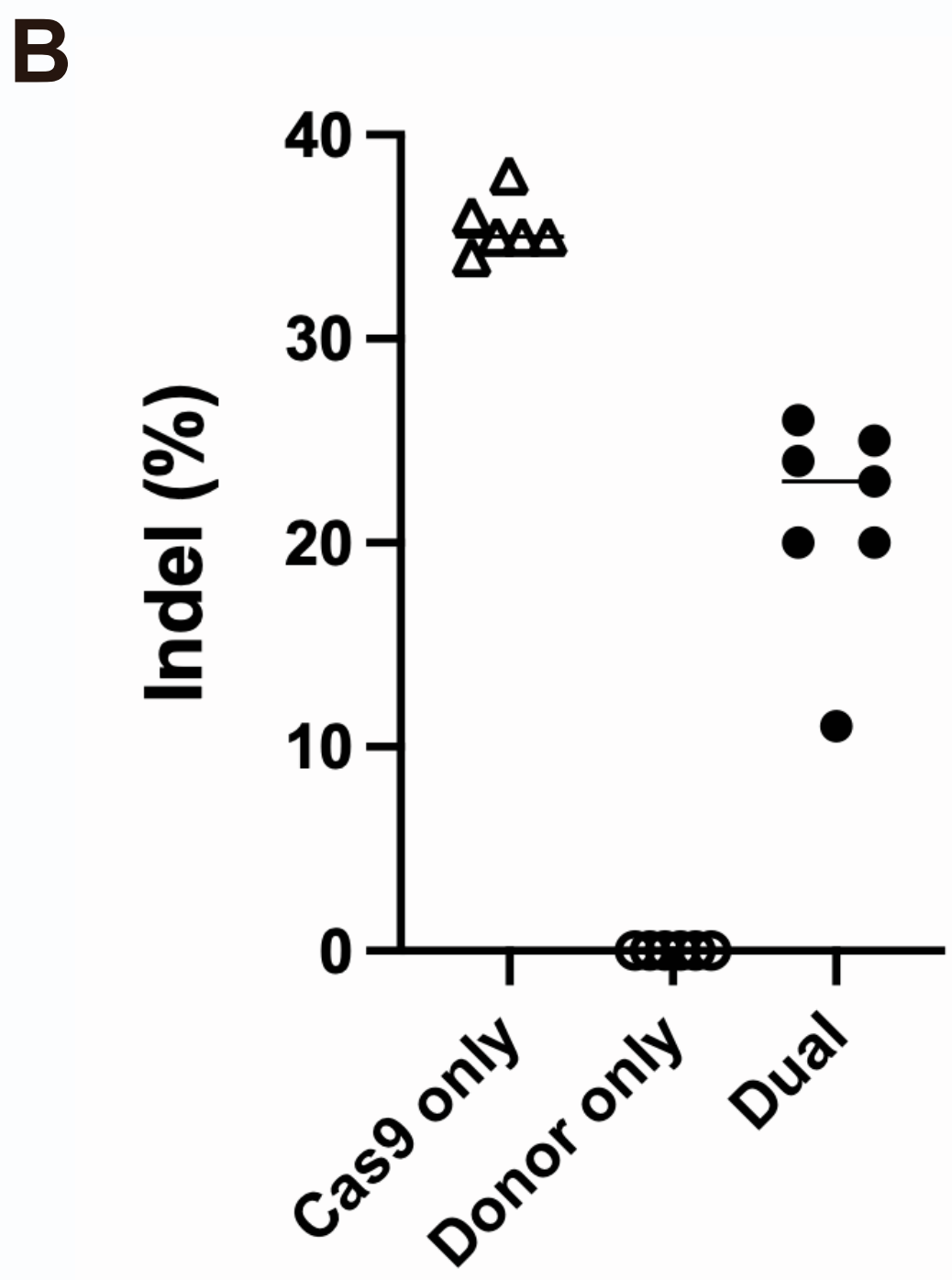

Figure S9.
Quantification of indel frequency following targeted integration. (A) Location of primers and sgRNA (rectangle) and PAM (triangle) sequences in the first intron of the murine Otc locus. (B) Genomic DNA was isolated from whole liver 8 weeks after dual vector delivery and the percentage of indels determined by PCR using primers that span the predicted SaCas9 cleavage site followed by Sanger sequencing of the amplicons (n=6-7 per treatment group, mean ± SEM are indicated). Animals receiving either the donor or editing reagents were included as controls.

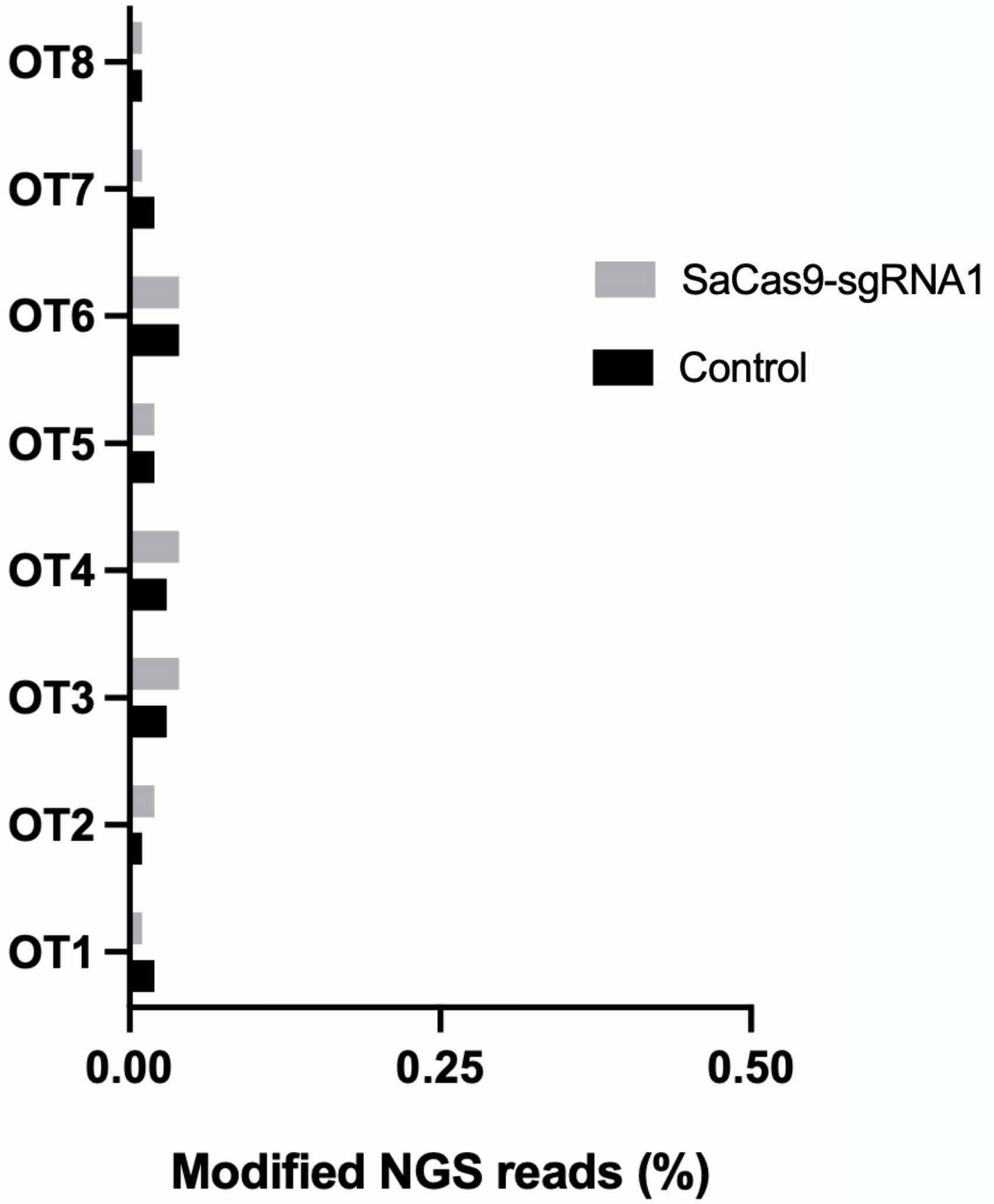

Figure S10.
Analysis of predicted off-target sites in the human genome. Amplicons from both a treated and control FRG mice, engrafted with primary human hepatocytes, were mixed in equimolar ratios and NGS sequencing using a 2 × 150 paired-end configuration (PE150) were performed. Modified reads in both the treated and control samples are expressed as a percentage to the total. Off-target sites were identified using GT-Scan (https://gt-scan.csiro.au/). All values were between 0.01 and 0.04% of the total reads.

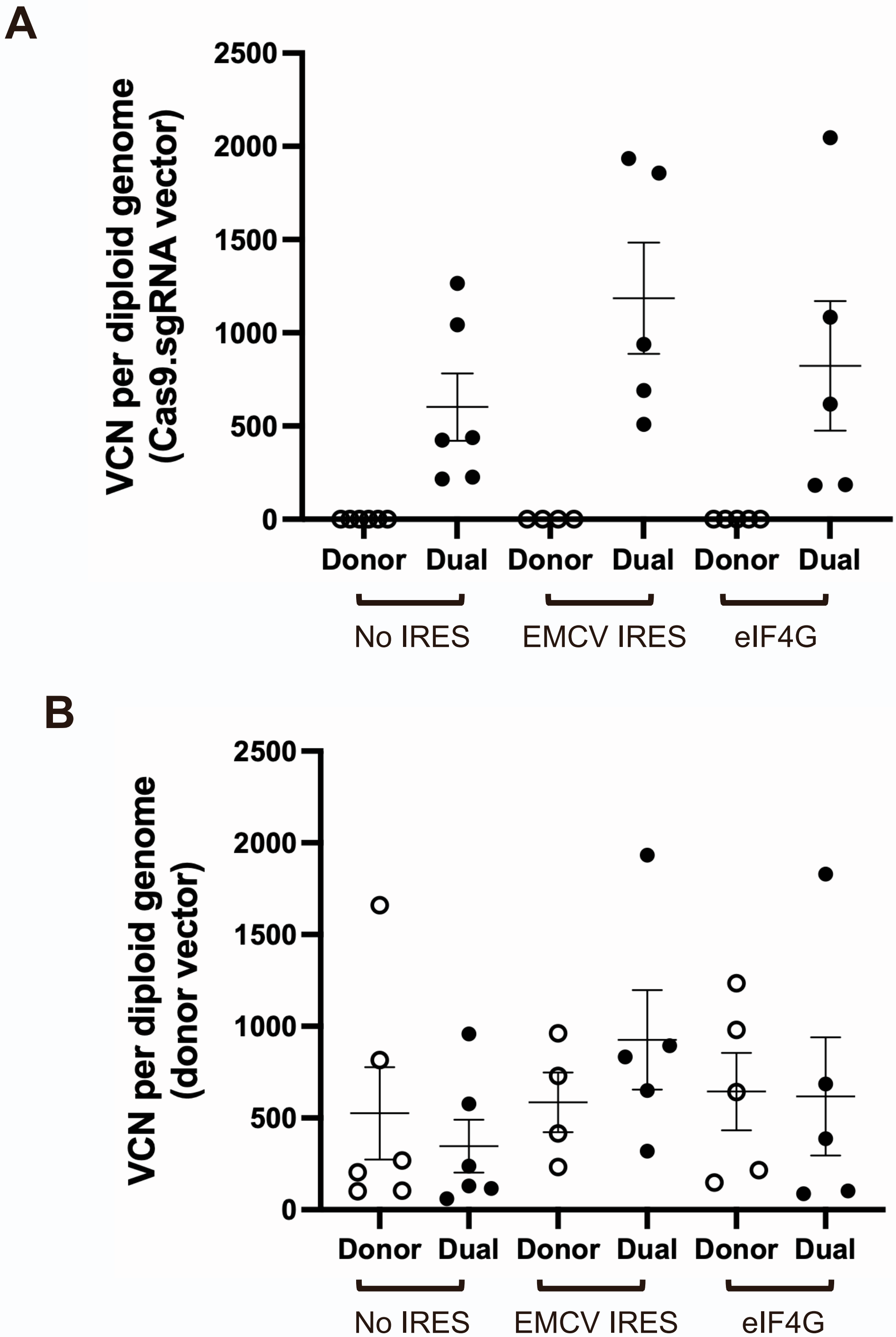

Figure S11.

Quantification of vector copy number (VCN) following dual AAV delivery in engrafted FRG mice. Vector copy number of (A) the SaCas9 and (B) EGFP transgenes per diploid genome in genomic DNA isolated from whole liver were determined by digital PCR (n=4-6 per treatment group). Mean ± SEM for each of the three donor configurations are indicated and animals receiving only the donor vectors were included as controls. All vectors were packaged in the NP59 capsid.

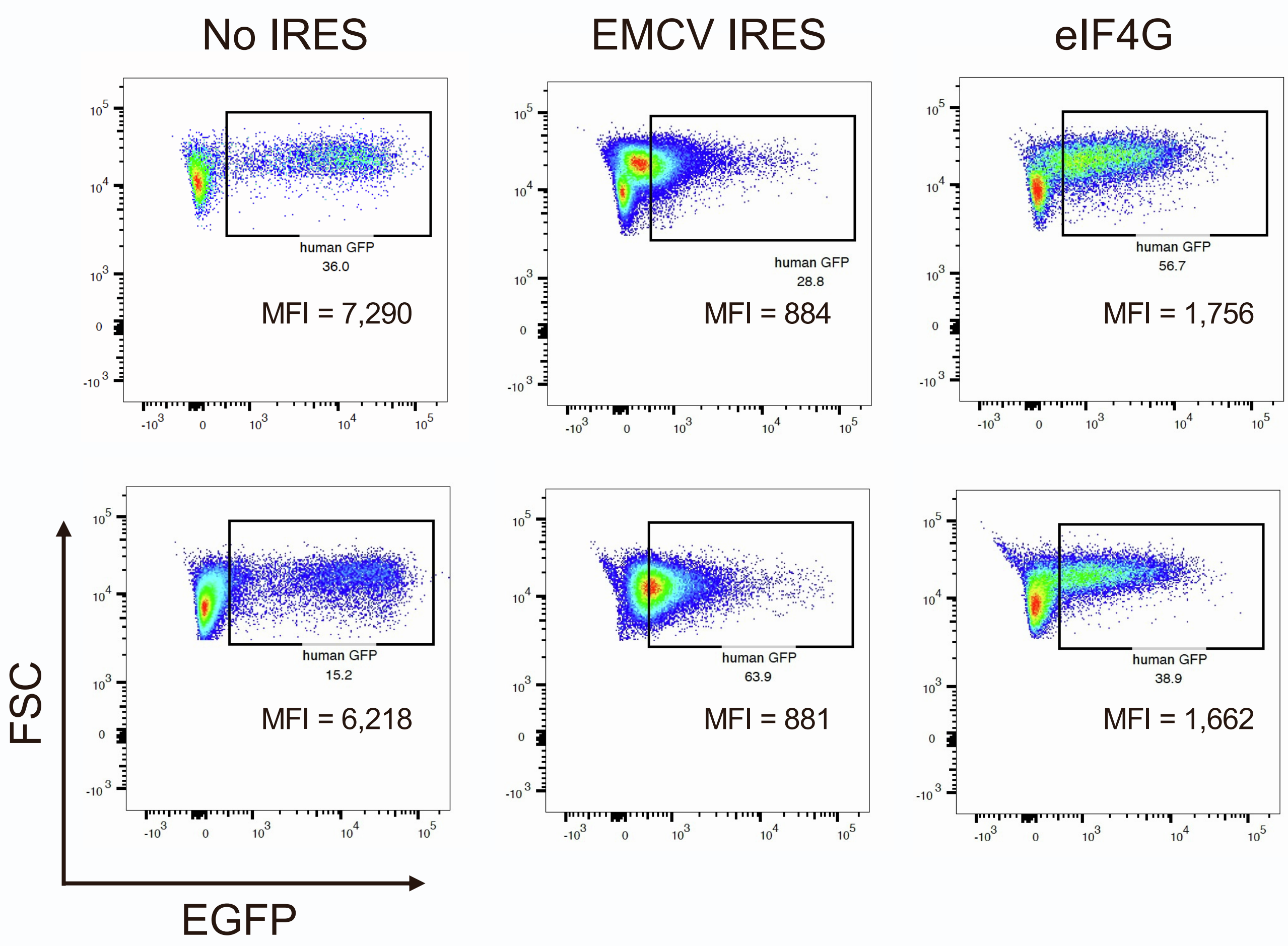

Figure S12.

Influence of the inclusion of an IRES element on gene expression levels. Fluorescent activated cell sorting (FACS) analysis of perfused chimeric mouse-human livers following dual AAV delivery to determine the percentage of EGFP positive human hepatocytes. The mean fluorescence intensity (MFI) for each of the three donor vector configurations are shown (n=2 animals per treatment group shown).

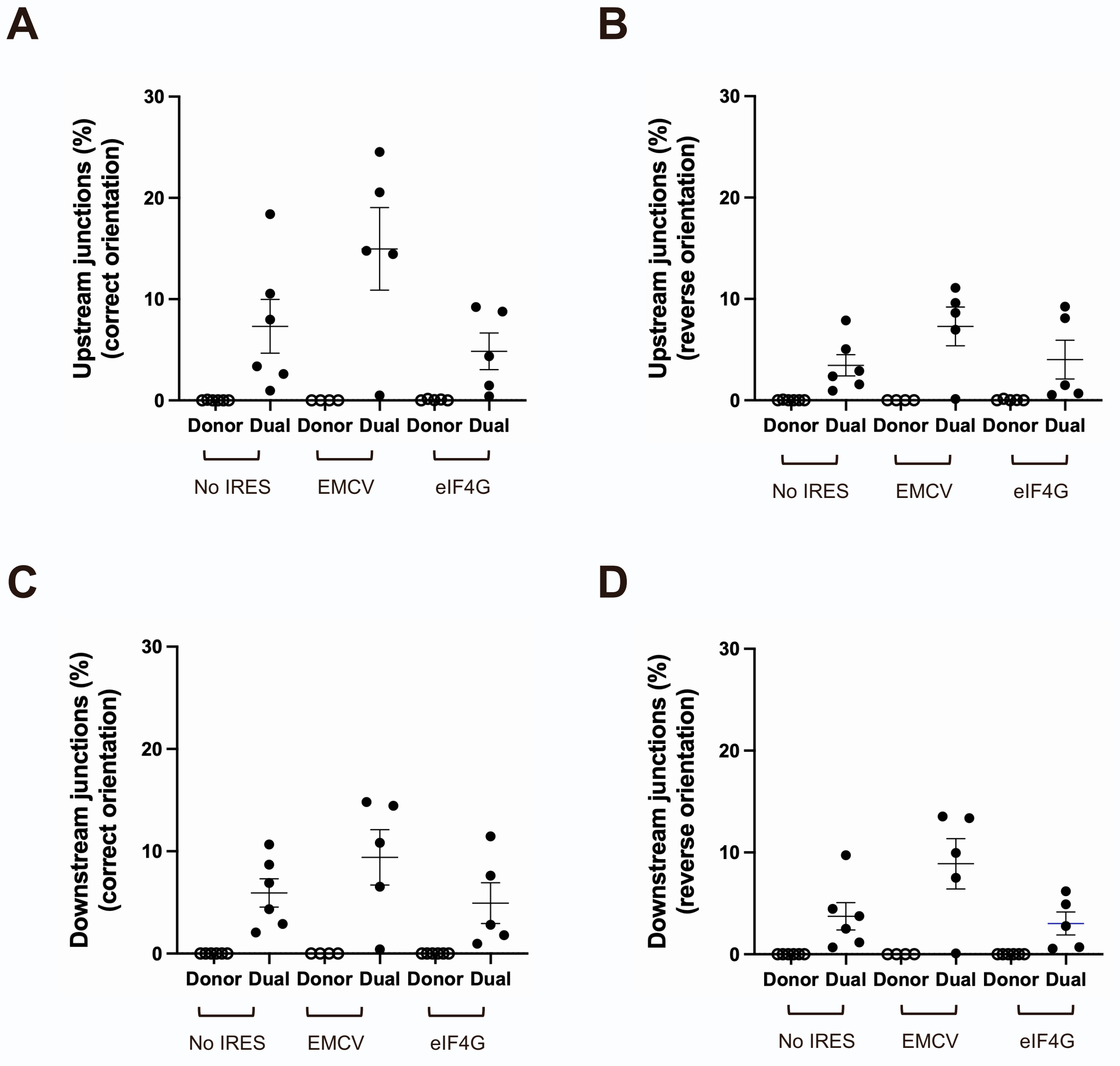

Figure S13.
Quantification of precise HITI junctions between the human genome and integrated EGFP-containing donor cassettes. Genomic DNA was isolated from whole liver 6 weeks after vector delivery and predicted junctions into the target OTC locus were quantified by digital PCR (A) upstream junctions in the correct orientation, (B) upstream junctions in the reverse orientation, (C) downstream junctions in the correct orientation, (D) downstream stream junctions in the reverse orientation (n=5 or 6 per treatment group, mean ± SEM are indicated).

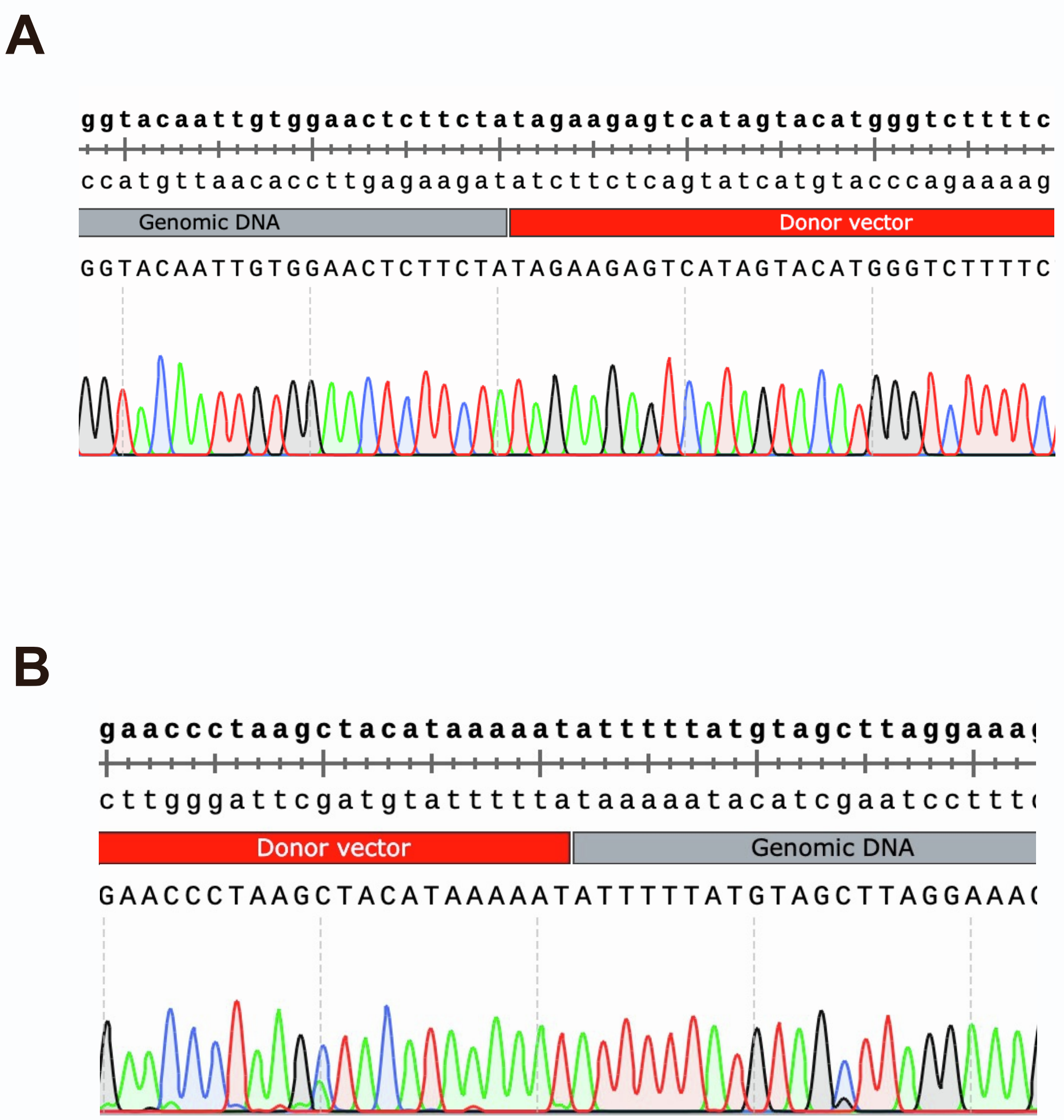

Figure S14.
Identification of predicted HITI junctions between the human genome and integrated EGFP donor. (A) Upstream and (B) downstream junctions were confirmed by PCR using primers that span the predicted junction followed by Sanger sequencing of the amplicons.

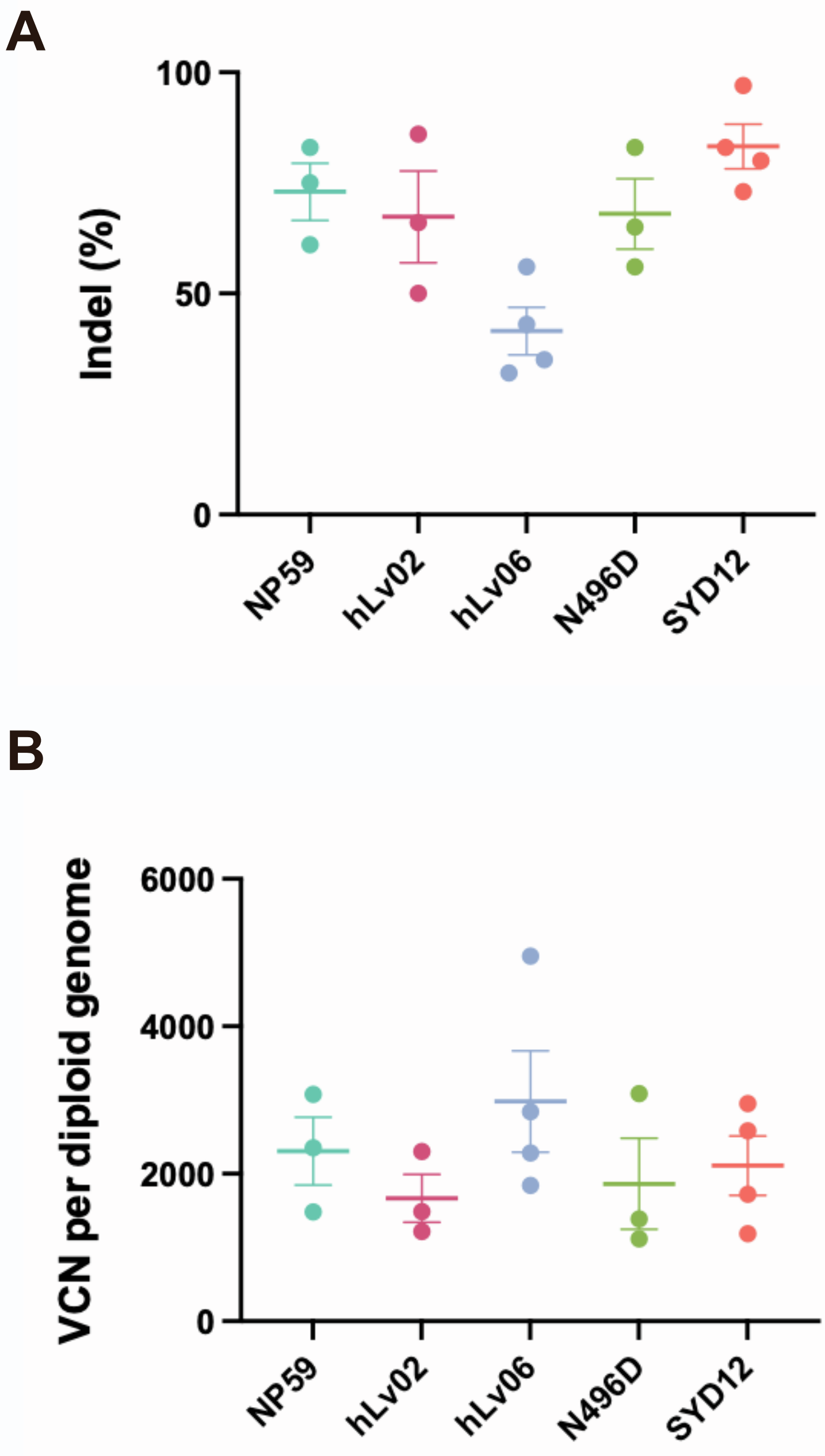

Figure S15.
Comparison of editing efficiency using selected human liver-tropic capsids. (A) The percentage of indels in genomic DNA from chimeric mouse-human livers 6 weeks after vector delivery was determined by PCR using primers that span the predicted SaCas9 cleavage site followed by Sanger sequencing of the amplicons. (B) Quantification of vector copy number (VCN) by digital PCR. (n=3-4 per capsid, mean ± SEM are shown).

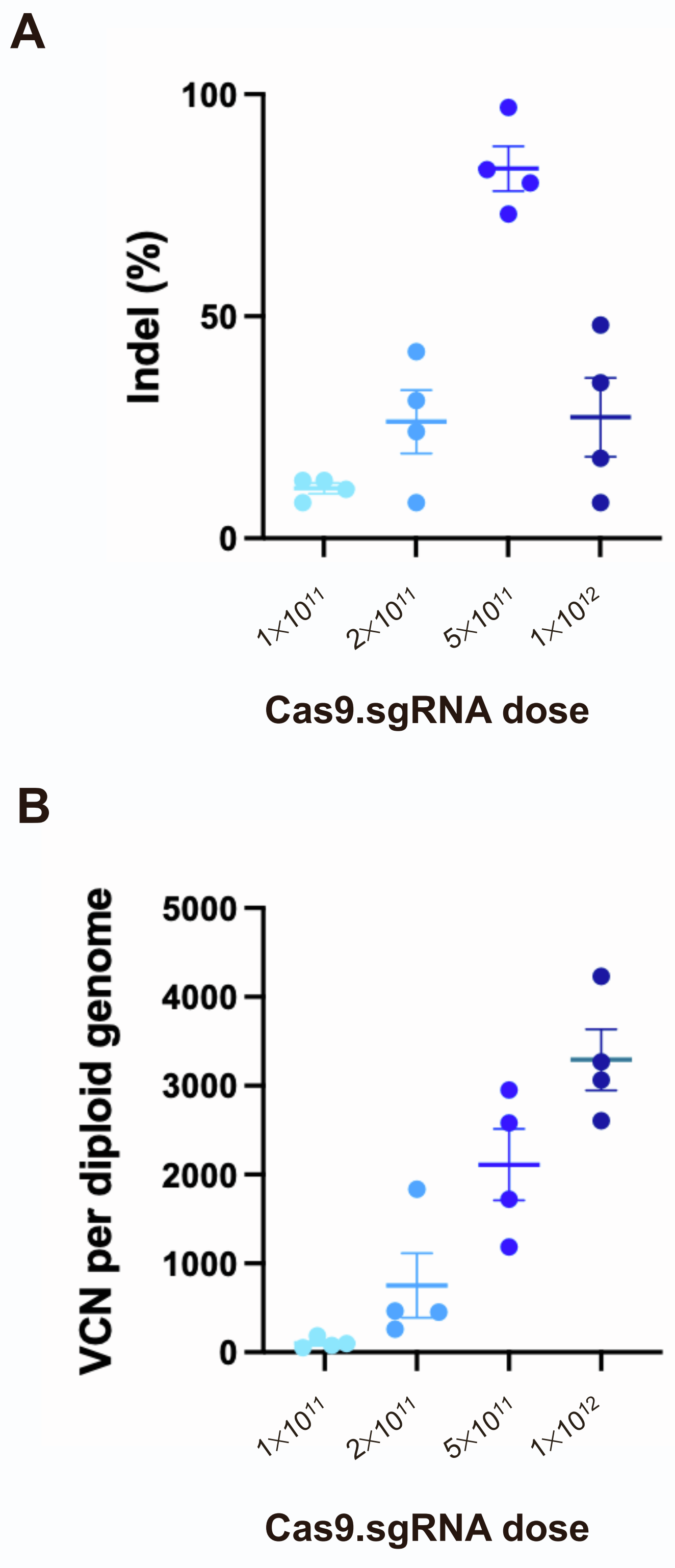

Figure S16. Determination of optimal vector dose for *in vivo* editing using the SYD12 capsid. (A) The percentage of indels in genomic DNA from chimeric mouse-human livers 6 weeks after vector delivery was determined by PCR using primers that span the predicted SaCas9 cleavage site followed by Sanger sequencing of the amplicons. (B) Quantification of vector copy number (VCN) by digital PCR. (n=4 per treatment group, mean ± SEM are shown).

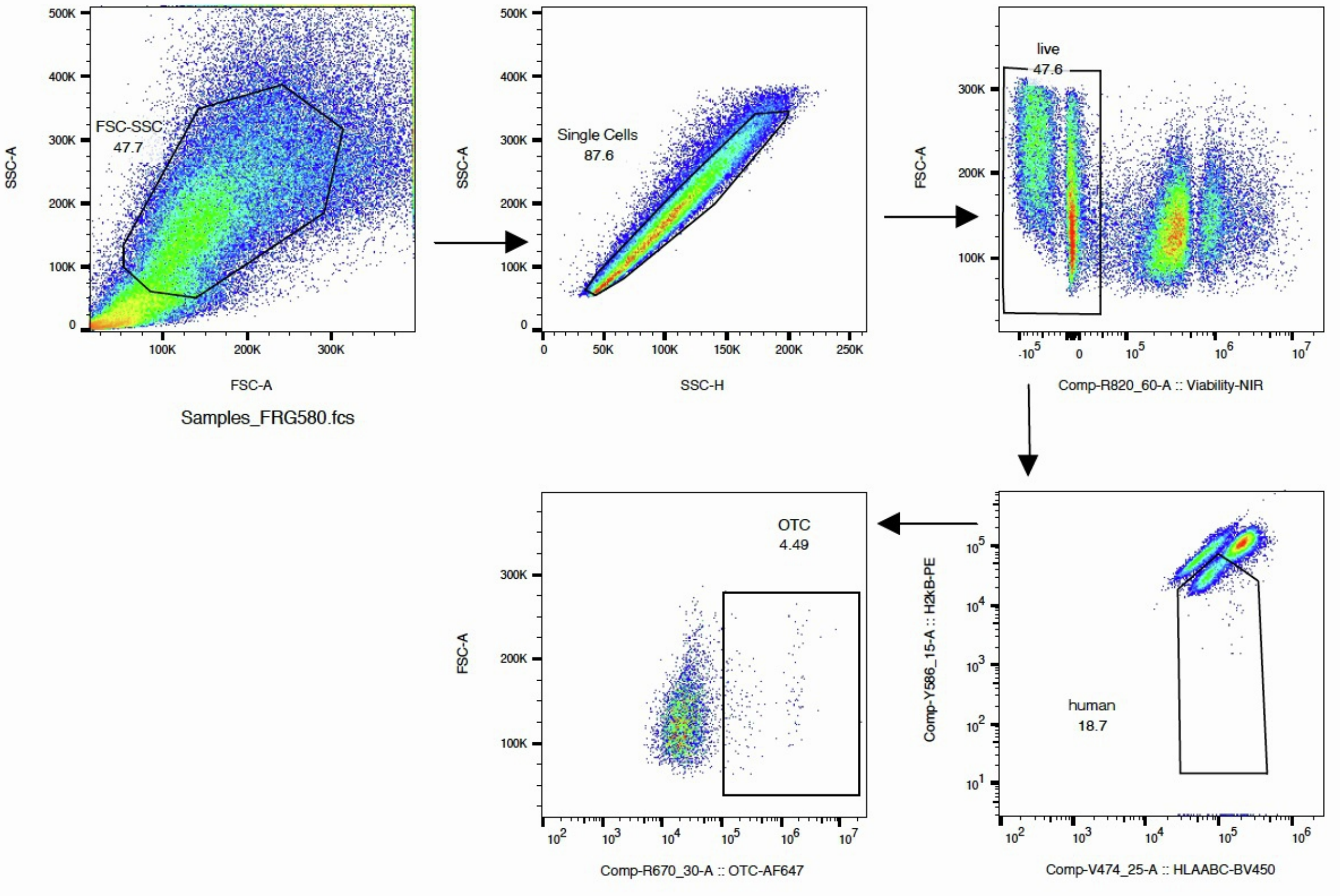

Figure S17.
Gating strategy used for to determine the percentage of OTC positive primary human hepatocytes in chimeric mouse-human livers in FRG mice using FACS analysis. Representative FACS profiles with gating strategy showing the percentage of human OTC positive hepatocytes 6 weeks after dual AAV delivery of 5×10^{11} vg of the editing and 1×10^{11} vg of the donor vector.

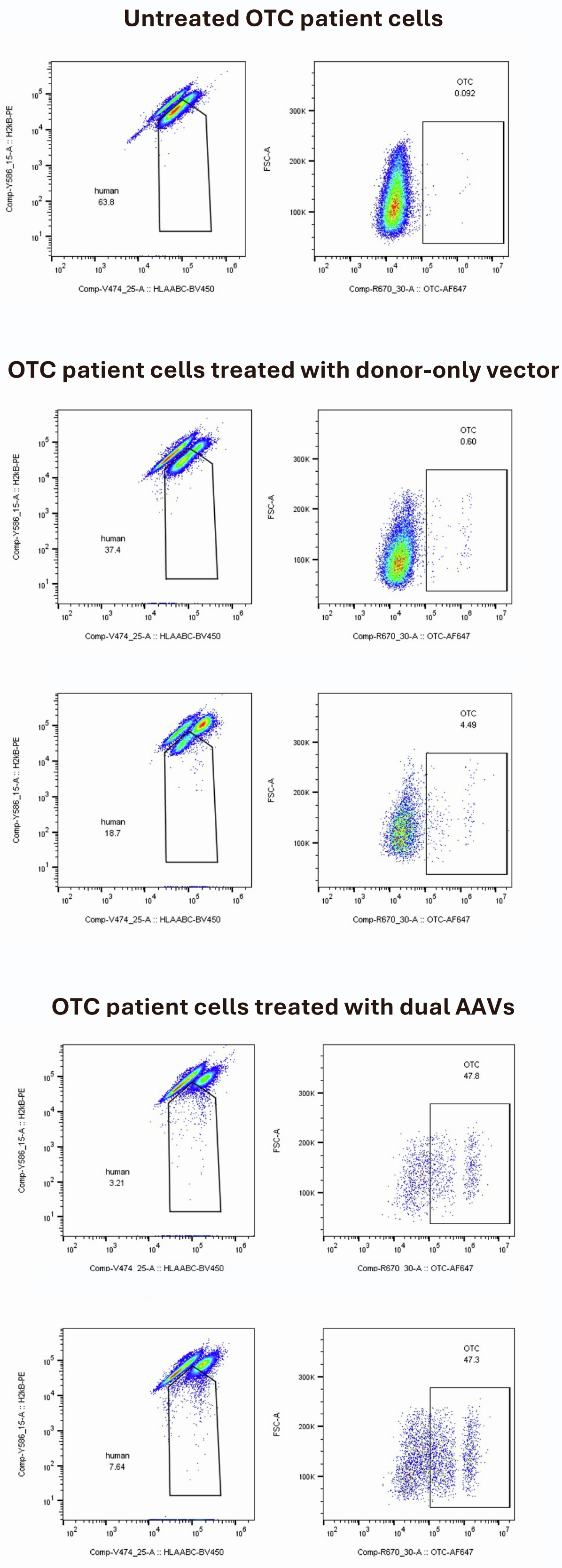

Figure S18.

Restoration of OTC expression in patient-derived primary human hepatocytes *in vivo* following dual vector delivery. The percentage of OTC positive hepatocytes from engrafted FRG animals receiving 5×10^{11} vg/mouse of the AAV vector containing the editing reagents and 1×10^{11} vg/mouse of the AAV donor packaged in the SYD12 capsid. Untreated and mice treated with only the donor vector are shown as controls.

A

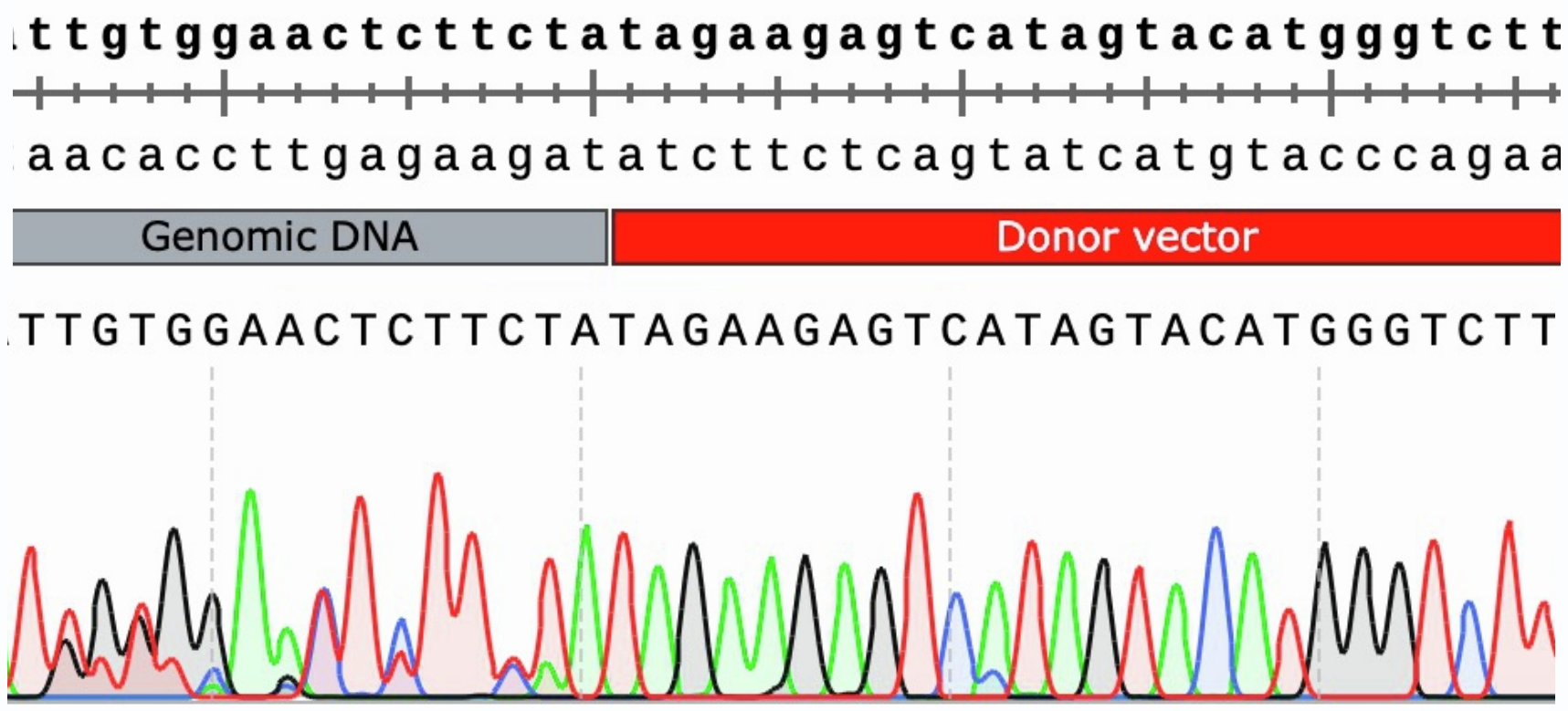

B

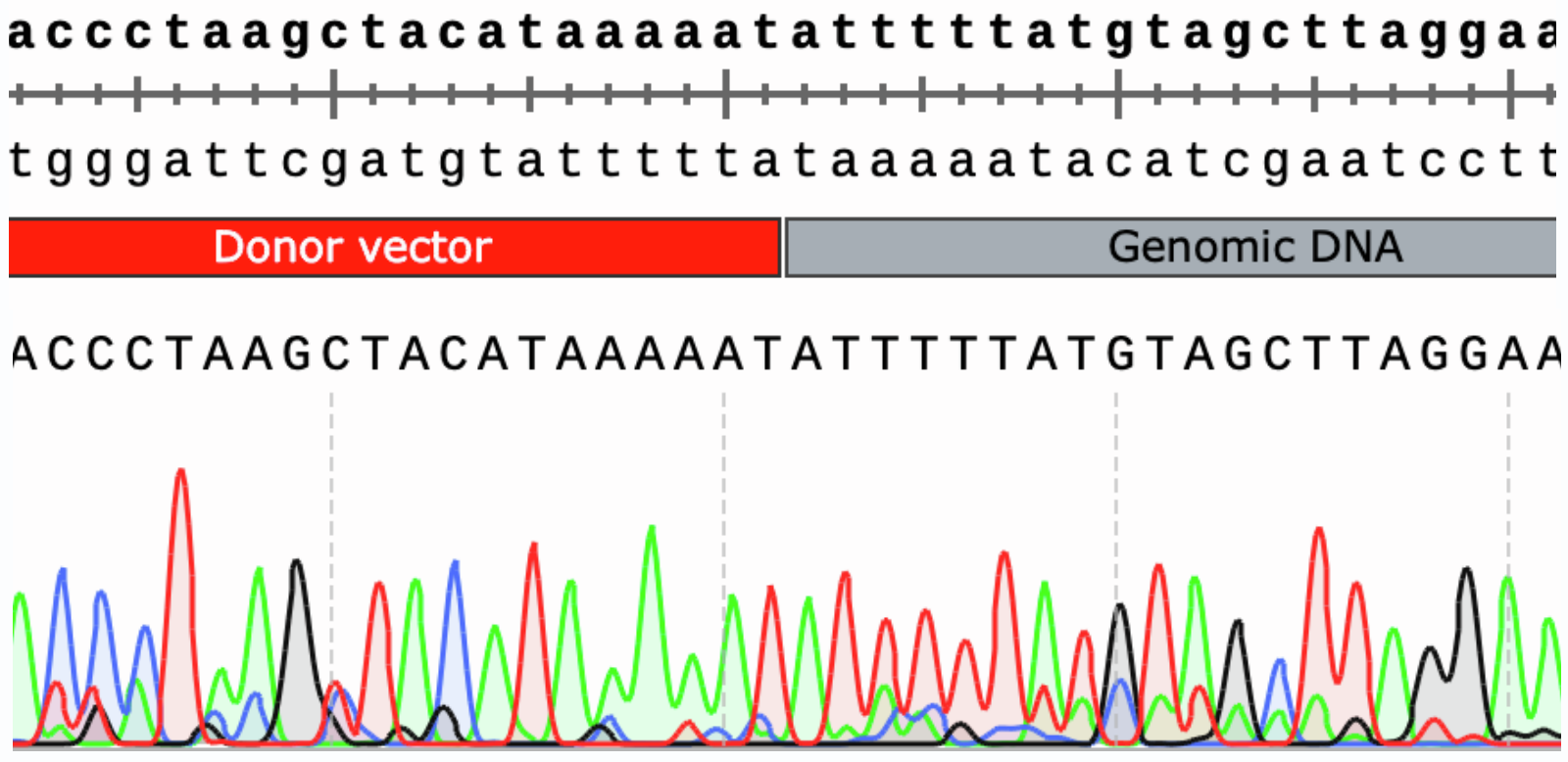

Figure S19.
Identification of predicted HITI junctions between the human genome and integrated OTC donor. (A) Upstream and (B) downstream junctions were confirmed by PCR using primers that span the predicted junction followed by Sanger sequencing of the amplicons.

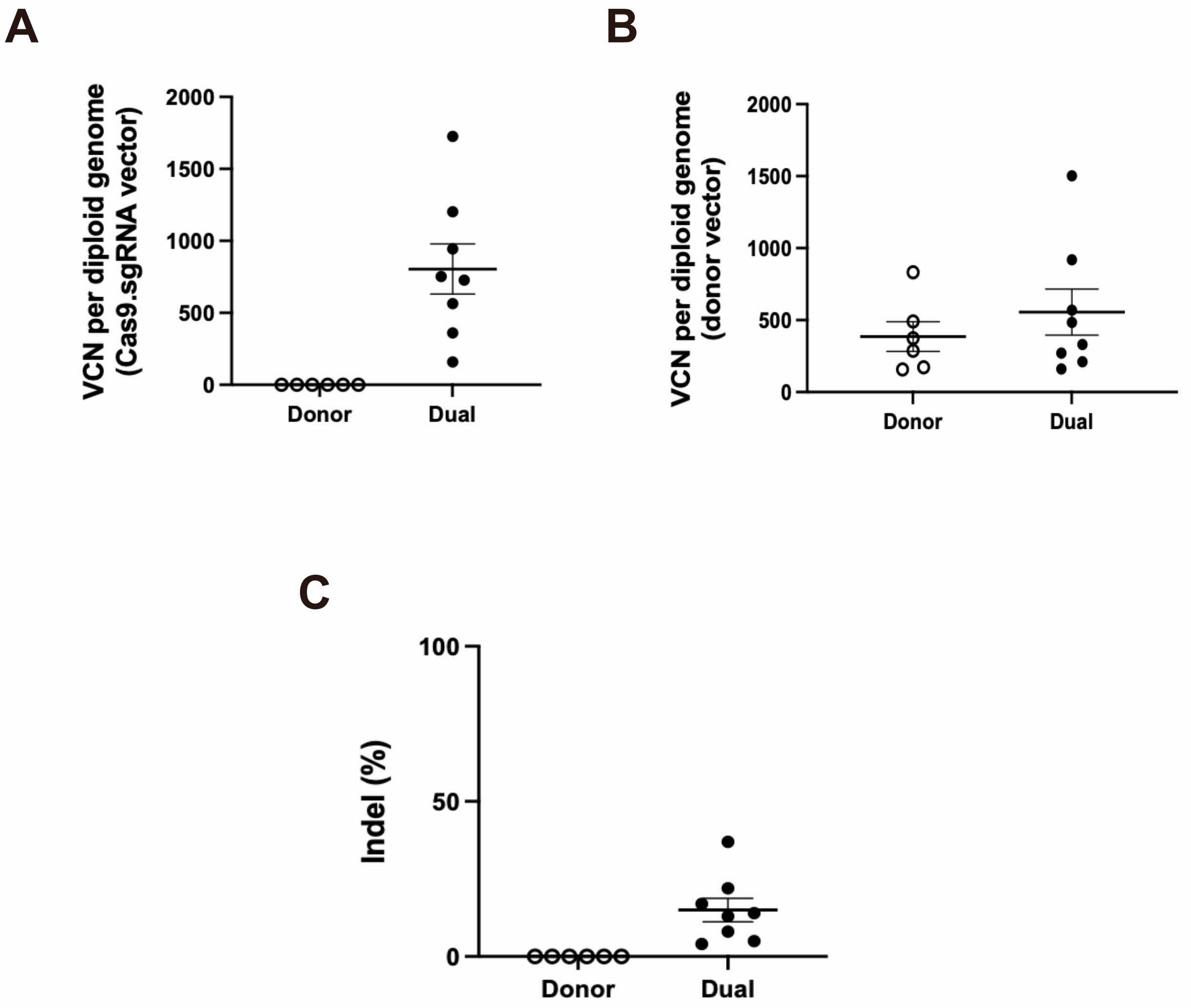

Figure S20.
Molecular analysis of FRG mouse livers engrafted with patient-derived primary human hepatocytes. Vector copy number of the (A) SaCas9 and (B) EGFP transgenes per diploid genome. (C) The percentage of indels identified in genomic DNA from chimeric mouse-human livers following delivery of dual AAV vectors (n=6 for the donor treatment group and n=8 for the dual treatment group, mean ± SEM are shown).

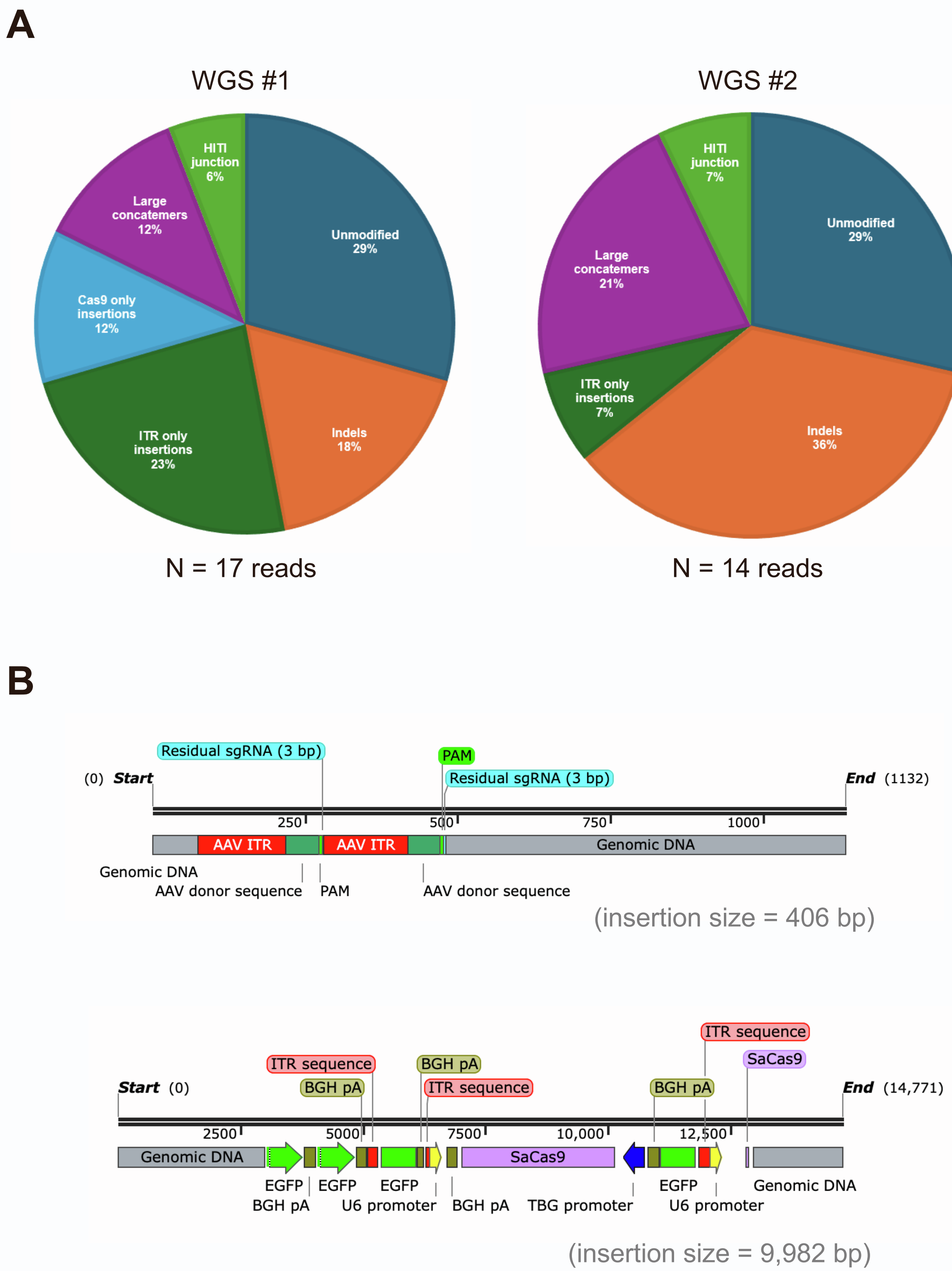

Figure S21.

Composition of integration events identified at the CRISPR-Cas9 cleavage site in murine intron 1. Genomic DNA was isolated from the liver of a C57Bl6 mouse treated with dual AAV vectors and subjected to long-read WGS to determine the types of integrations occurring at the CRISPR-Cas9 cleavage site. (A) Distribution of integration events and (B) Representative examples of integrations events observed at the target site.

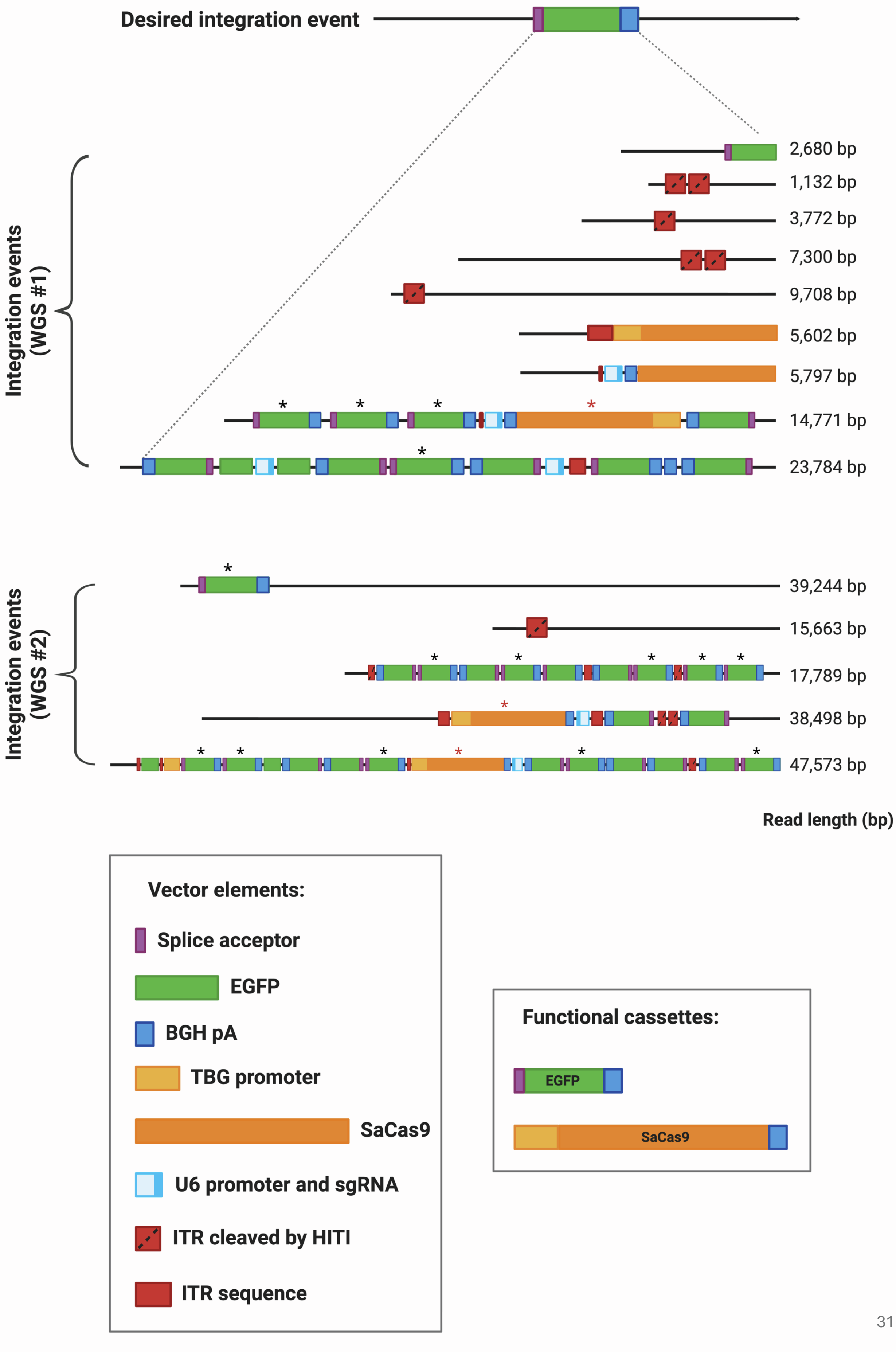
Desired integration event
Integration events (WGS #1)
2,680 bp
1,132 bp
3,772 bp
7,300 bp
9,708 bp
5,602 bp
5,797 bp
14,771 bp
23,784 bp
Integration events (WGS #2)
39,244 bp
15,663 bp
17,789 bp
38,498 bp
47,573 bp
Read length (bp)
Vector elements:
Splice acceptor
EGFP
BGH pA
TBG promoter
SaCas9
U6 promoter and sgRNA
ITR cleaved by HITI
ITR sequence
Functional cassettes:
EGFP
SaCas9

Figure S22.

Diagrammatic representation of the integration events identified at the target site in Otc intron 1. Genomic DNA was isolated from the liver of a C57Bl6 mouse treated with dual AAV vectors and subjected to long-read WGS. Total read lengths (bp) are indicated on the right. Integration events could be assigned to four main classes: the desired HITI event, insertion of ITR sequences cleaved from the donor vector, insertions containing the only the Cas9 vector or complex concatemeric sequences. For the two insertions containing only the Cas9 vector, the read did not contain the 3' junction so it was not possible to determine the full composition of the insertion or whether these events could lead to Cas9 expression. Donor cassettes that would be predicted to be functional, i.e. containing correctly orientated splice acceptor sequences (purple), full length EGFP transgene (green) and BGH pA (blue), are indicted by a black asterisk. Integrations of Cas9 vector that would be predicted to be functional are indicated by a red asterisk. Due to the complex nature of the concatemeric insertions, not all vector elements have been annotated. BGH pA, bovine growth hormone poly adenylation sequence; EGFP, enhanced green fluorescent protein, ITR, inverted terminal repeat; TBG, thyroxine-binding globulin promoter. Not to scale.
